# Genomic analysis of 11,555 probands identifies 60 dominant congenital heart disease genes

**DOI:** 10.1073/pnas.2420343122

**Published:** 2025-03-24

**Authors:** Michael C. Sierant, Sheng Chih Jin, Kaya Bilguvar, Sarah U. Morton, Weilai Dong, Wei Jiang, Ziyu Lu, Boyang Li, Francesc López-Giráldez, Irina Tikhonova, Xue Zeng, Qiongshi Lu, Jungmin Choi, Junhui Zhang, Carol Nelson-Williams, James R. Knight, Hongyu Zhao, Junyue Cao, Shrikant Mane, Stanley C. Sedore, Peter J. Gruber, Monkol Lek, Elizabeth Goldmuntz, John Deanfield, Alessandro Giardini, Seema Mital, Mark Russell, J. William Gaynor, Eileen King, Michael Wagner, Deepak Srivastava, Yufeng Shen, Daniel Bernstein, George A. Porter, Jane W. Newburger, Jonathan G. Seidman, Amy E. Roberts, Mark Yandell, H. Joseph Yost, Martin Tristani-Firouzi, Richard Kim, Wendy K. Chung, Bruce D. Gelb, Christine E. Seidman, Martina Brueckner, Richard P. Lifton

**Affiliations:** ^a^Department of Genetics, Yale School of Medicine, New Haven, CT 06510; ^b^Laboratory of Human Genetics and Genomics, The Rockefeller University, New York, NY 10065; ^c^Department of Genetics, Washington University School of Medicine, St. Louis, MO 63110; ^d^Department of Pediatrics, Washington University School of Medicine, St. Louis, MO 63110; ^e^Yale Center for Genome Analysis, Yale University, New Haven, CT 06516; ^f^Department of Neurosurgery, Yale School of Medicine, New Haven, CT 06510; ^g^Yale Program on Neurogenetics, Yale School of Medicine, New Haven, CT 06510; ^h^Department of Medical Genetics, School of Medicine, Acibadem University, Istanbul 34752, Türkiye; ^i^Department of Translational Medicine, Health Sciences Institute, Acibadem University, Istanbul 34752, Türkiye; ^j^Division of Newborn Medicine, Department of Pediatrics, Boston Children’s Hospital, Boston, MA 02115; ^k^Manton Center for Orphan Disease Research, Boston Children’s Hospital, Boston, MA 02115; ^l^Broad Institute of Massachusetts Institute of Technology and Harvard, Boston, MA 02142; ^m^Department of Biostatistics, Yale School of Public Health, New Haven, CT 06510; ^n^Laboratory of Single-Cell Genomics and Population Dynamics, The Rockefeller University, New York, NY 10065; ^o^Department of Biostatistics and Medical Informatics, University of Wisconsin, Madison, WI 53706; ^p^Department of Biomedical Sciences, Korea University College of Medicine, Seoul, South Korea; ^q^Department of Pediatrics, Section of Cardiology, Yale School of Medicine, New Haven, CT 06510; ^r^Department of Pediatrics, Michigan State University College of Human Medicine, Grand Rapids, MI 48824; ^s^Department of Surgery, Yale University School of Medicine, New Haven, CT 06510; ^t^Division of Cardiology, Children’s Hospital of Philadelphia, Department of Pediatrics, Perelman School of Medicine, University of Pennsylvania, Philadelphia, PA 19104; ^u^Institute of Cardiovascular Science, University College London, London WC1E 6BT, United Kingdom; ^v^Pediatric Cardiology, Great Ormond Street Hospital, London WC1N 3JH, United Kingdom; ^w^Division of Cardiology, Department of Pediatrics, The Hospital for Sick Children, University of Toronto, Toronto, ON M5G1X8, Canada; ^x^Department of Pediatrics and Communicable Diseases, University of Michigan, Ann Arbor, MI 48109; ^y^Division of Cardiothoracic Surgery, Children's Hospital of Philadelphia, Philadelphia, PA 19104; ^z^Department of Pediatrics, University of Cincinnati, Cincinnati, OH 45229; ^aa^Division of Biomedical Informatics, Cincinnati Children’s Hospital Medical Center, Cincinnati, OH 45229; ^bb^Division of Biostatistics and Epidemiology, Cincinnati Children’s Hospital Medical Center, Cincinnati, OH 45229; ^cc^Gladstone Institute of Cardiovascular Disease and University of California San Francisco, San Francisco, CA 94158; ^dd^Department of Systems Biology, Columbia University Irving Medical Center, New York, NY 10032; ^ee^Department of Biomedical Informatics, Columbia University Irving Medical Center, New York, NY 10032; ^ff^Department of Pediatrics, Cardiology, Stanford University, Stanford, CA 94304; ^gg^Department of Pediatrics, The School of Medicine and Dentistry, University of Rochester Medical Center, Rochester, NY 14642; ^hh^Department of Cardiology, Boston Children’s Hospital, Harvard Medical School, Boston, MA 02115; ^ii^Department of Pediatrics, Harvard Medical School, Boston, MA 02115; ^jj^Department of Genetics, Harvard Medical School, Boston, MA 02115; ^kk^Department of Human Genetics, University of Utah and School of Medicine, Salt Lake City, UT 84112; ^ll^The Catholic University of America, Washington, DC 20064; ^mm^Division of Pediatric Cardiology, University of Utah, Salt Lake City, UT 84112; ^nn^Pediatric Cardiac Surgery, Smidt Heart Institute, Cedars-Sinai Medical Center, Los Angeles, CA 90048; ^oo^Department of Pediatrics, Boston Children’s Hospital, Harvard Medical School, Boston, MA 02115; ^pp^Department of Pediatrics and Medicine, Columbia University Medical Center, New York, NY 10032; ^qq^Mindich Child Health and Development Institute, Icahn School of Medicine at Mount Sinai, New York, NY 10029; ^rr^Department of Pediatrics, Icahn School of Medicine at Mount Sinai, New York, NY 10029; ^ss^Cardiovascular Division, Brigham and Women’s Hospital, Boston, MA 02115; ^tt^HHMI, Chevy Chase, MD 20815

**Keywords:** congenital heart disease, molecular inversion probes, exome sequencing, human genetics, genomics

## Abstract

Congenital heart disease (CHD) is a leading cause of infant mortality. We analyzed de novo mutations (DNMs) and very rare transmitted/unphased damaging variants in 248 prespecified genes in 11,555 CHD probands. The results identified 60 genes with a significant burden of heterozygous damaging variants. Variants in these genes accounted for CHD in 10.1% of probands with similar contributions from de novo and transmitted variants in parent–offspring trios that showed incomplete penetrance. DNMs in these genes accounted for 58% of the signal from DNMs. Thirty-three genes were linked to a single CHD subtype while 12 genes were associated with 2 to 4 subtypes. Seven genes were only associated with isolated CHD, while 37 were associated with 1 or more extracardiac abnormalities. Genes selectively expressed in the cardiomyocyte lineage were associated with isolated CHD, while those widely expressed in the brain were also associated with neurodevelopmental delay (NDD). Missense variants introducing or removing cysteines in epidermal growth factor (EGF)-like domains of NOTCH1 were enriched in tetralogy of Fallot and conotruncal defects, unlike the broader CHD spectrum seen with loss of function variants. Transmitted damaging missense variants in *MYH6* were enriched in multiple CHD phenotypes and account for ~1% of all probands. Probands with characteristic mutations causing syndromic CHD were frequently not diagnosed clinically, often due to missing cardinal phenotypes. CHD genes that were positively or negatively associated with development of NDD suggest clinical value of genetic testing. These findings expand the understanding of CHD genetics and support the use of molecular diagnostics in CHD.

## Significance

We identified 60 genes with significant burden of monoallelic damaging variants, accounting for 10.1% of probands, with equal contributions from de novo and transmitted variants. Mutations often produced variable congenital heart disease (CHD) and extracardiac (EC) phenotypes. Mutation frequency was variable and not explained by mutability. Probands with mutations associated with syndromic CHD were frequently not clinically diagnosed, often due to the absence of characteristic phenotypes. Genes with predominantly transmitted variants were enriched for isolated CHD and depleted for extracardiac features including neurodevelopmental disorders (NDD). There was wide variation in risk of NDD (e.g., 4% for *MYH6* mutation vs. 95% for *CHD7*). Results support increased use of molecular diagnosis to assess risk and allow early intervention to potentially improve outcomes.

Congenital heart disease (CHD) is one of the most common congenital malformations, affecting 1.0 to 1.8% of all live births (1, 2). One-third of patients require intervention within the first year of life, and while ~90% will now survive to adulthood, many will have lifelong comorbidities including neurodevelopmental disabilities, arrhythmia, and heart failure (3, 4).

Recurrence of CHD in families (5, 6), Mendelian forms of CHD, and CHD with aneuploidy, recurring chromosomal deletions and duplications, established a genetic contribution to CHD (7). Whole exome sequencing (WES) in parent–offspring trios by the Pediatric Cardiac Genomics Consortium (PCGC) demonstrated that damaging de novo mutations (DNMs) contribute to CHD (8–11). DNMs were highly enriched for loss of function (LOF) mutations in genes intolerant to LOFs (high pLI), consistent with haploinsufficiency, with gain of function (GOF) mutations in a few genes such as *PTPN11* (12). The role of transmitted variants has been less well-studied (10, 12). Results suggest that DNMs in hundreds of genes contribute to CHD, with only seven genes implicated as having a significant burden of DNMs after the study of 2,645 trios (10). Genes involved in chromatin modification were highly enriched for DNMs (8–10).

The need for much larger cohorts is evident. Knowledge from prior results can help prioritize a subset of genes with increased likelihood of being CHD genes (13) that can be selectively sequenced using molecular inversion probe sequencing (MIPseq) at very low cost (14–16). A challenge of MIPseq has been achieving complete coverage of targeted bases (15).

We report herein analyses of a targeted panel of 248 genes in 11,555 CHD probands from the PCGC and Pediatric Heart Network (PHN) cohorts who have extensive cardiac and extracardiac (EC) clinical phenotyping and have undergone WES or MIPseq, increasing the number of CHD probands fourfold from our most recent study (10). We identify 60 genes with significant enrichment of damaging variants that contribute to CHD in 10.1% of probands, with nearly equal contributions from de novo and transmitted variants. We show striking variable expressivity of phenotypes resulting from variants in many of these genes.

## Results

### A MIPseq Panel for Candidate CHD Genes.

From earlier analysis of WES in 1,213 parent–offspring trios (9) and prior reports of genes associated with CHD, we selected 248 CHD genes to target for further study (*SI Appendix*, Fig. S1, Dataset S1, and *Materials and Methods*). We synthesized and optimized 10,154 barcoded molecular inversion probes to capture, sequence, and annotate the entire coding sequence of each gene and flanking splice regions (*SI Appendix*, Figs. S2–S4 and Dataset S2). Analysis of rare variants called using a reference sample (17) and CHD samples with prior WES data (9) demonstrated very high sensitivity and precision of variant calls (*SI Appendix*, Fig. S6), at an all-in production cost of $25.50 per sample compared to $170 per sample for WES at the time of processing, reducing the cost of sequencing of these samples by 85%.

### Assembly of a Cohort of 11,555 CHD Probands.

MIPseq was performed on 5,929 PCGC probands (*Materials and Methods* and Dataset S3); >95% of targeted bases had ≥10 independent reads (*SI Appendix*, Fig. S7); diverse very rare variants were validated by Sanger sequencing (*SI Appendix*, Table S1). Additional WES increased totals to 3,887 CHD trios and 1,739 singletons, which combined with MIPseq probands yielded 11,555 CHD probands (*SI Appendix*, Table S2 and Dataset S3). Cardiac, structural EC, and neurodevelopmental (NDD; neurodevelopmental delay, learning disability, intellectual disability, or autism, based on parental/adult proband report of physician diagnosis at age ≥1 y) phenotypes were obtained at the time of recruitment (*SI Appendix*, Table S3 and *Materials and Methods*). The presence of structural EC and neurodevelopmental disorders (NDD) across different CHD subtypes departed from the null distribution (chi-square *P* < 2.2 × 10^−16^). For example, 56% of probands with hypoplastic left heart syndrome (HLHS) had EC features, while ECs were found in only 28% of probands with left ventricular outflow tract abnormalities (LVO) (*SI Appendix*, Fig. S8).

### Pathogenic Variants in Known and New CHD Genes.

De novo and transmitted variants in trios and unphased variants in singleton probands were called and annotated for impact on encoded proteins and allele frequency (10) (*Materials and Methods* and Dataset S4). The primary analytic strategy was a meta-analysis that combined i) the significance of the burden of damaging DNMs in the 248 panel genes in probands compared to expectation; ii) the significance of the frequency of very rare damaging variants [MAF < 10^−5^ in both the BRAVO Freeze 3a WGS database (18) and Exome Sequencing Project v25 (ESP) (19) WES database] in these genes that were transmitted to probands in trios or found in singleton probands (referred to as transmitted and unphased variants, TUVs) compared to their frequency among up to 135,743 individuals in the gnomAD database (20) (*Materials and Methods*).

DNM’s showed no enrichment of synonymous or tolerated missense variants (“T-mis”), but highly significant enrichment for both LOF (1.49-fold enriched, *P* = 4.63 × 10^−19^) and damaging missense (D-mis) DNMs (1.35-fold enriched, *P* = 1.24 × 10^−13^) (*SI Appendix*, Table S4A). Across all ~19,000 genes, the combined excess LOF and D-mis DNMs implicated damaging DNMs in 9.1% of CHD probands (*SI Appendix*, Table S4A). This enrichment is dramatically increased when only the 248 panel genes are considered, with de novo LOFs enriched 14.3-fold, *P* < 10^−117^, and de novo D-mis variants enriched 6.1-fold, *P* < 10^−58^ (*SI Appendix*, Table S4B). These damaging DNMs can explain 6.6% of all probands, comprising 72% of the DNM signal. The strong enrichment in this 248 gene set is not the result of ascertainment bias, because exclusion of the 1,213 trios used to identify this gene set, leaving only the 2,686 additionally sequenced trios, yielded highly concordant estimates of enrichment of LOF and D-mis DNMs and percentage of probands explained in this panel (LOF enrichment 14.5-fold, D-mis 5.6; 6.3% of probands explained; *SI Appendix*, Table S5B).

Next, the prevalence of very rare TUVs in each panel gene was compared between probands and gnomAD WES & WGS controls, after adjustment for per-base sequence coverage (*Materials and Methods*). The frequencies of synonymous and T-mis variants showed no significant difference between CHD probands and gnomAD controls, while LOF and D-mis variants were highly significantly enriched in CHD probands (1.48-fold enriched, *P* < 10^−21^ and 1.11-fold enriched, *P* < 10^−10^, respectively) (*SI Appendix*, Table S6A). For comparison, we assessed LOF and D-mis variants in 28 MIPseq panel genes that were not LOF-intolerant (pLI < 0.9), lacked protein-altering DNMs in the initial 1,213 CHD probands (10), and were not previously implicated in CHD. These 28 genes showed no enrichment in any variant functional class (*SI Appendix*, Table S6B) (*Materials and Methods*). The remaining 220 MIPseq genes showed highly significant enrichment for damaging, but not synonymous or T-mis variants (*SI Appendix*, Table S6C).

**Fig. 1.**
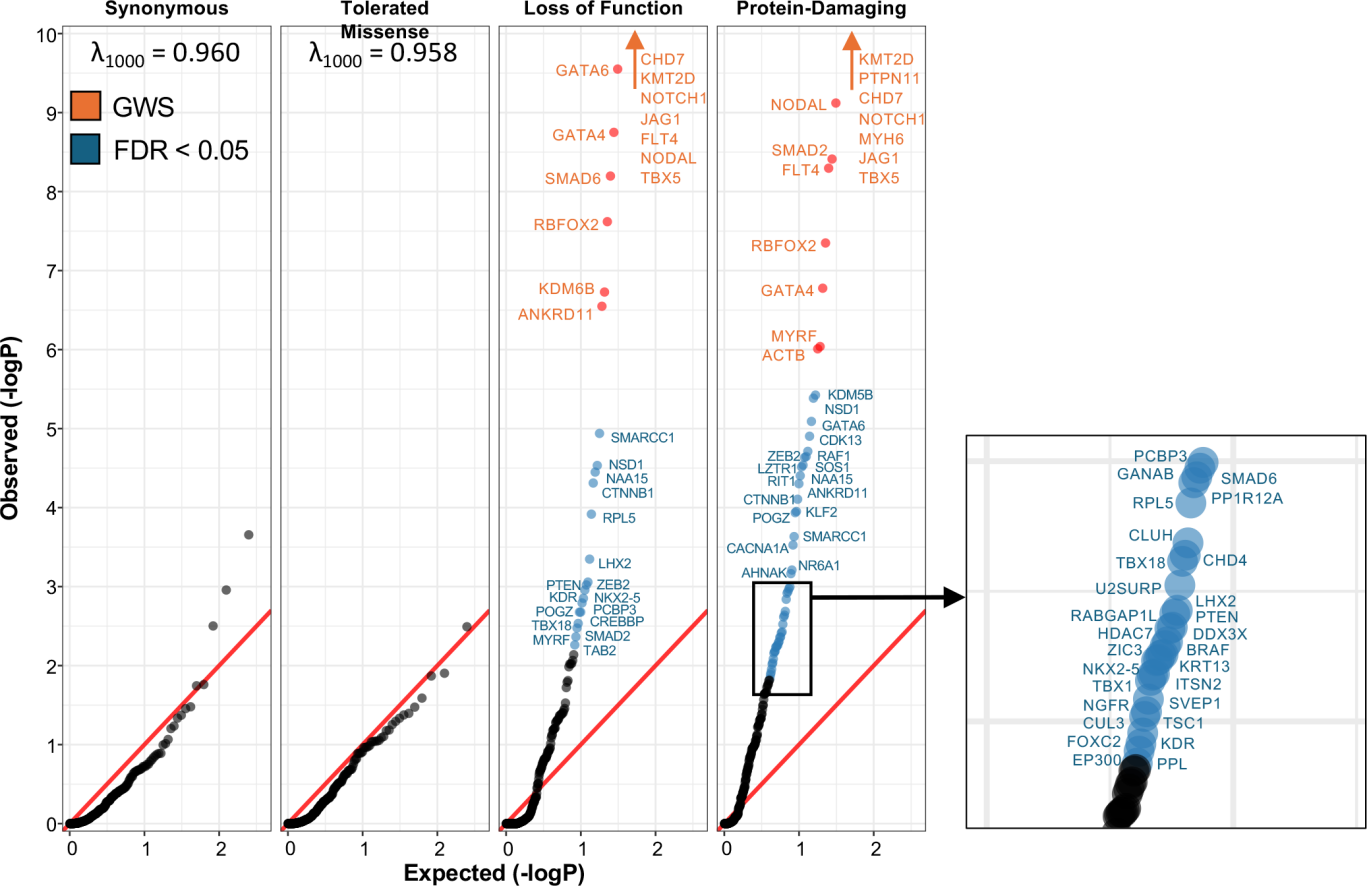
Quantile–quantile plots of meta-analysis using DNMs and TUVs in 248 genes in 11,555 probands. For indicated variant classes, Q–Q plot is shown. Genes surpassing genome-wide significance (*P*-value < 2.6 × 10^−6^) and those with FDR < 0.05 are shown in red and blue, respectively. The y-axis has been truncated at –logP = 10. Red arrows denote genes that have *P* < 10^−10^. The genomic inflation factor, λ, adjusted per 1,000 samples is shown for syn and T-Mis variants. In Damaging variant results, the boxed *Inset* shows genes with observed −logP from 1.5 to 3.0.

Results from the DNM and case–control tests were combined for each panel gene by meta-analysis and significance was adjusted for multiple-testing correction using the joint-local FDR (JL-FDR) method (*Materials and Methods*) (21). Quantile–quantile plots showed that synonymous and T-mis variants adhered to the expected distribution; no genes had JL-FDR < 0.05. In contrast, *P*-values for LOF and protein-damaging variants showed marked departure from expectation (Fig. 1).

Sixty genes had a significant excess of damaging variants. For 48 of these genes, the case–control data strengthened the significance from DNM alone, and for 14 of these genes, the case–control data were critical to the association. Thirteen of these 60 genes have not previously been implicated in CHD, three have only been previously reported in animal models or from biochemical studies in vitro, and 17 have been previously reported anecdotally in human CHD or without supporting statistical evidence (*SI Appendix*, Table S7). Significance of some genes was attributable to LOF variants (e.g., *JAG1, NODAL*), D-mis variants (e.g., *PTPN11, MYH6*), or to contributions from both (e.g., *NOTCH1, KMT2D*) (*SI Appendix*, Table S8). Variants in *GDF1* were predominantly biallelic with a founder effect (10) and were eliminated from analysis of these monoallelic variants. Data for the remaining 188 panel are shown in Dataset S6.

As another test of significance of these 60 genes, we performed the transmission disequilibrium test (TDT) (22) on very rare damaging variants in the 3,887 trios (*Materials and Methods*). Damaging mutations were overtransmitted in the entire 248 gene panel (*SI Appendix*, Table S9A), with nearly the entire signal coming from the 60 significant genes (*SI Appendix*, Table S9 B and C). 78% of LOF variants (118/152) and 59% of D-mis variants were transmitted to probands (chi-square *P* = 9.5 × 10^−12^ and 2.6 × 10^−6^, respectively). Significance remains after excluding the two most transmitted genes, *MYH6* and *NOTCH1* (*SI Appendix*, Table S9D). Results for genes with the greatest over transmission are shown in *SI Appendix*, Table S9E. TDT results for all 60 significant genes and the 248 targeted genes are shown in Dataset S7.

Damaging DNMs in the 60 significant genes can account for CHD in 5.2% of all probands (*SI Appendix*, Table S10A), comprising 58% of the total signal from damaging DNMs. After removal of these 60 genes, a highly significant residual signal for damaging DNMs persists among the remaining 188 genes in the targeted set (*P* = 3.8 × 10^−19^) accounting for another 1.4% of probands (*SI Appendix*, Table S10B). Overtransmission of damaging variants in these same genes can explain another 5.4% of probands (*SI Appendix*, Table S10C), with little evidence of significant residual signal in the remaining 188 genes (*SI Appendix*, Table S10D). By chance, 0.3% of probands are expected to have variants in two genes in the 60 gene set, and were observed in 0.4% of probands, not significantly different from expectation. In total, 10.1% of probands had one or more damaging variants in the 60 gene set.

### Association of Mutated Genes with Specific CHD Phenotypes.

The relatively large number of damaging variants in the 60 significant genes provides the opportunity to assess the variability of CHD phenotypes arising from variants in each. Adjusting for multiple comparisons (*Materials and Methods*), we found that damaging variants in 33 genes, including *GATA6, FLT4, SMAD2,* and *KLF2* were significantly associated with a single cardiac phenotype (Table 1). Twelve other genes showed striking variability in their associated CHDs. These included *NOTCH1*, which was associated with CTD, TOF, LVO, and HLHS; *KMT2D* with CTD, LVO, and HLHS; and *PTPN11* with CTD, ASD, and AVC (Table 1). The remaining 15 genes had smaller numbers of damaging variants that were not significantly associated with any phenotypic subset.

**Table 1.** Damaging variant meta-analysis identifies genes significantly enriched CHD probands with one or more cardiac, EC, or neurodevelopmental phenotypes

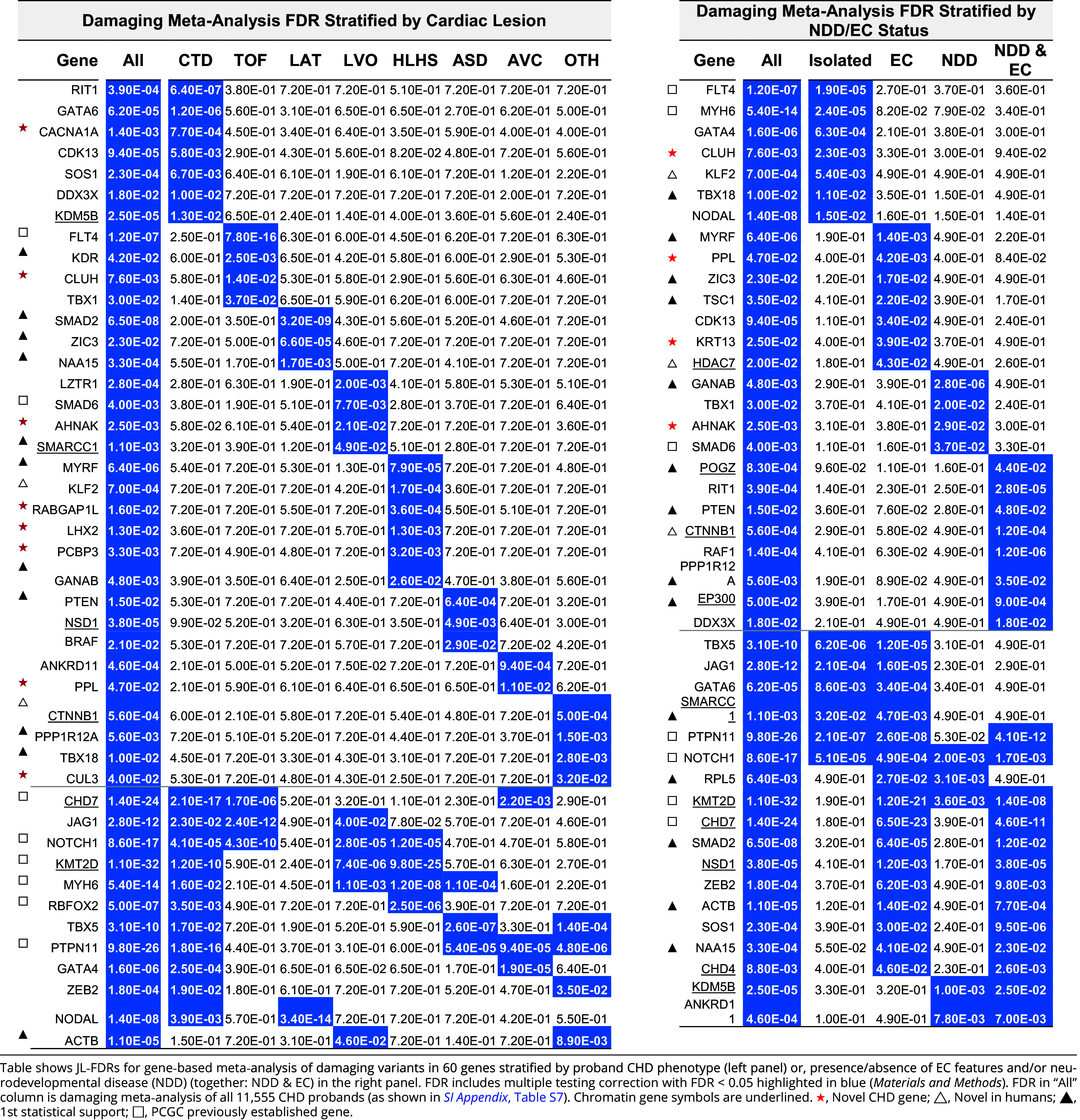

Damaging DNMs were found most frequently in patients with ASD (7.9% of probands) and HLHS (7.3%), and least frequently in those with laterality (LAT) defects (2.1%) and TOF (3.6%). Damaging transmitted monoallelic variants were most frequent in ASD (7.9%) and TOF (7.6%) and were not significantly overtransmitted in LAT and AVC (*SI Appendix*, Table S11).

### Association of CHD Genes with EC Phenotypes.

The distribution of EC phenotypes resulting from damaging variants was also not random (*P* < 10^−4^ by Monte Carlo simulation). Variants in each of the 60 genes were tested for association with isolated CHD, structural EC, and NDD (*SI Appendix*, Table S1B). Some genes were associated only with one of these groups (e.g., *MYH6* with isolated CHD; *MYRF* with CHD plus EC*; GANAB* with CHD plus NDD; *RAF1* with CHD plus EC and NDD). Others were associated with multiple phenotypes (e.g., *TBX5*, *NOTCH1*, *KMT2D*), demonstrating variable expressivity. 74% (56/76) of probands with damaging DNMs in NDD-associated genes and 9% (3/35) with damaging DNMs in non-NDD-associated genes had NDD.

Overall, probands with isolated CHD were least likely to have a damaging DNM (5.0%), and those with CHD plus NDD & EC were most likely (29.2%) (*SI Appendix*, Table S12). Conversely, transmitted damaging variants were most frequent in probands with isolated CHD (6.6%), and negligible in probands with either NDD alone (0.2%) or NDD plus EC (0.002%) (*SI Appendix*, Table S11), providing evidence of impaired reproductive fitness for variants causing NDD.

### Role of Developmental Expression Patterns in Manifestation of EC Phenotypes.

**Fig. 2.**
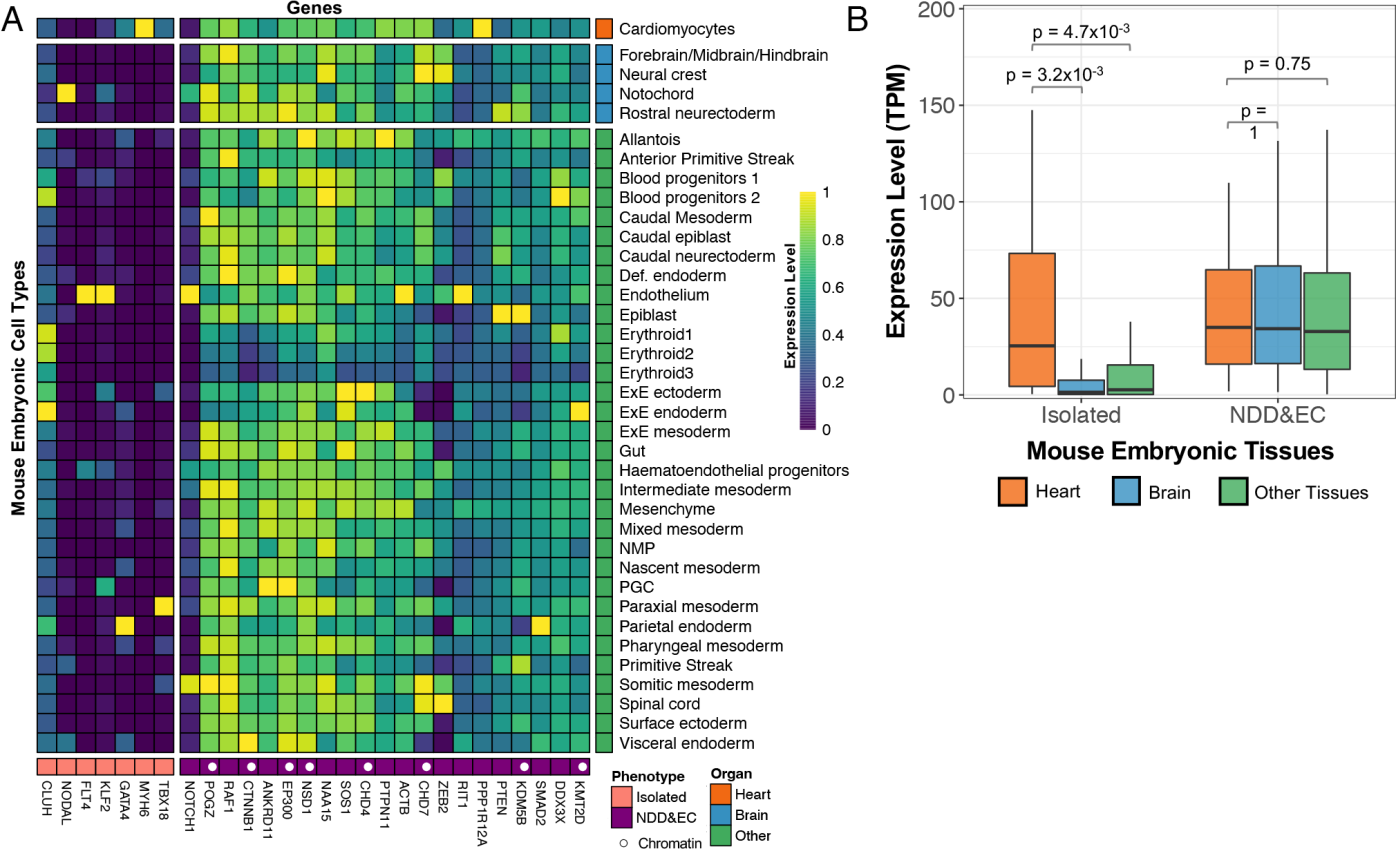
Single-cell expression during mouse gastrulation of genes enriched in probands with isolated CHD or NDD & EC. (*A*) Single-cell RNA-seq from mouse gastrulation (24) for 28 genes associated with isolated CHD probands (in pink) or NDD & EC (in purple) are shown. On the vertical axis, cell types are sorted for heart (red), brain (blue), and other tissues (green). Expression levels are scaled to the greatest TPM (Transcripts per Million Reads) for each gene across all tissues. Chromatin genes are annotated with a white dot in the purple bar. (*B*) Quantitation of expression of genes expressed in heart, brain, and other tissues comparing genes associated with isolated CHD and NDD & EC. Median values are shown on each boxplot. A Wilcoxon rank-sum *P*-value comparing expression levels in heart/brain/other tissues is shown for genes associated with isolated vs. NDD & EC probands.

We anticipated that genes associated with isolated CHD would show expression profiles restricted to cardiovascular cell types whereas genes associated with NDD plus EC would have broader expression, including the brain. Single-cell gene expression at mouse gastrulation (6.5 to 8.5 gestational days) (23) confirmed this expectation (Fig. 2). This finding was corroborated in an expression dataset of human fetal development from 72 to 129 d postconception (24) (*SI Appendix*, Fig. S9).

### Chromatin Modifier Genes.

Damaging de novo variants were sixfold enriched (*P* < 10^−52^) among chromatin-modifying genes studied (*SI Appendix*, Table S13A). Ten showed significant mutation burden (*SI Appendix*, Table S13B), predominantly due to de novo LOFs; nonetheless, all 13 LOFs seen in parents were transmitted to probands. Transmitted LOFs predominated for *SMARCC1* (six transmitted, zero DNMs), with six more in singletons. DNMs in these 10 genes comprise 69% of the total chromatin gene signal, 20% of the entire DNM signal and explain 1.8% of probands in trios (*SI Appendix*, Table S13C) and 8.3% of probands with EC and NDD (*SI Appendix*, Table S12). After exclusion of these 10 genes, significant signal from the remainder of this gene set remains (*SI Appendix*, Table S13C).

Several of these genes are associated with a single CHD subgroup (e.g., *KDM5B* with CTD, *SMARCC1*), while others (e.g., CHD7, KMT2D) are associated with multiple CHD subgroups (Table 1). All show association with EC phenotypes (Table 1) and are broadly expressed (Fig. 2).

### Transmitted Damaging Missense Variants in *MYH6* in Multiple CHD Subtypes.

*MYH6* encodes the embryonic cardiac α-myosin heavy chain, and has previously been associated with a small number of patients with dilated and hypertrophic cardiomyopathy (25), ASD (25), and HLHS (26). We previously linked biallelic *MYH6* variants to multilevel left ventricular outflow obstruction (10).

*MYH6* had a twofold excess of monoallelic D-mis variants (FDR < 10^−10^ *SI Appendix*, Table S8A). TDT showed that 68/97 D-mis variants were transmitted from parents to probands (70%, *P* = 7.5 × 10^−5^), while LOFs were much less frequent and not overtransmitted (*SI Appendix*, Table S9E). D-mis *MYH6* variants were significantly associated with and over transmitted in LVO, HLHS, and ASD (*SI Appendix*, Table S14 A and B). Transmitted variants contributed to 2 to 3% of probands in each of these subgroups and nearly 1% of all trios studied. The genotypic risk ratio of transmitted variants in these groups was 6.0, a large effect.

Family histories in families of probands with HLHS, LVO, or ASD and transmitted D-mis *MYH6* variants identified structural heart disease in 3 transmitting (two with ASD) and 0 nontransmitting parents, two siblings, and 7 aunts, uncles, or first cousins among 32 kindreds. 8 of 10 affected family members were on the transmitting lineage. The low frequency of clinical CHD among transmitting parents provides evidence of incomplete penetrance of these variants, though subclinical CHD cannot be excluded without echocardiography.

The distribution of transmitted, nontransmitted, and de novo very rare D-mis variants in *MYH6* is shown in *SI Appendix*, Fig. S10. Interestingly, two de novo D-mis variants in *MYH6* were recurrent; a both are absent in BRAVO, EVS, and gnomAD databases. Seven transmitted variants were recurrent and were transmitted to probands by 16 of 19 carrier parents (84% transmission, chi-square *P* = 2.8 × 10^−3^) (Dataset S8).

Consistent with selective expression in the cardiomyocyte lineage (Fig. 2 and *SI Appendix*, Fig. S9), probands with *MYH6* D-mis variants predominantly had isolated CHD (Table 1); only 1/24 probands with transmitted variants and LVO, HLHS, or ASD, had NDD.

### LOF and Cysteine Variants in *NOTCH1*.

Notch signaling plays a broad role in developmental cell fate decisions (27). NOTCH1 is a transmembrane cell surface receptor; its 36 extracellular EGF repeats, when bound by cell surface ligands such as JAGGED 1 on adjacent cells, trigger a signaling cascade. There were 37 LOF variants distributed throughout the *NOTCH1* coding region, highly enriched in DNM and case–control analysis (*SI Appendix*, Fig. S11 and Table S15A), and associated with CTD, TOF, LVO, and HLHS (Table 1 and *SI Appendix*, Table S15A). *NOTCH1* LOFs were associated with isolated CHD, EC, NDD, and NDD + EC phenotypes (Table 1 and *SI Appendix*, Table S15A).

Eleven parents had LOF *NOTCH1* variants; all were transmitted to CHD probands. Three parents, all mutation carriers, had CHDs including aortic coarctation, bicuspid aortic valve (BAV), and pulmonary atresia/ventricular septal defect. Other relatives on the transmitting lineages had TOF, HLHS, and VSD, consistent with the spectrum of phenotypes found in probands. The absence of clinical CHD among the other 8 transmitting parents implies incomplete penetrance or subclinical CHD; echocardiograms have not been performed.

**Fig. 3.**
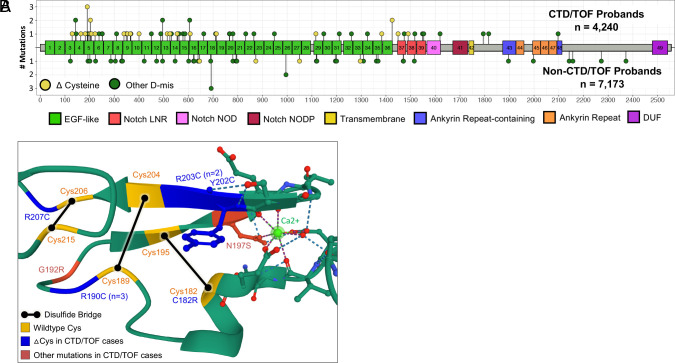
Frequent cysteine-altering mutations in *NOTCH1* EGF domain #5 in TOF/CTD probands. (*A*) NOTCH1 protein domain structure derived from Prosite and PFAM databases is shown, with EGF-like domains in green; amino acid positions in NOTCH1 are numbered on the x-axis. Lollipops show location of very rare D-mis variants in CHD probands; those in CTD/TOF probands are shown above the protein diagram and non-CTD-TOF probands are shown below. Variants that introduce or remove a cysteine are shown in yellow; all others are in green. Height of lollipops corresponds to number of independent occurrences of specific variants. “DUF” denotes “Domain of unknown function DUF3454.” (*B*) Crystal structure of *NOTCH1* EGF domain. Amino acids altered to or from cysteine in CTD/TOF probands (blue text), and other mutations in EGF #5 in CTD/TOF probands are in red; disulfide bridges in canonical protein are shown as black lines. Structure from ref. 30.

D-mis variants in *NOTCH1* were selectively enriched for two cardiac phenotypes, TOF (threefold enrichment; *P* = 2.3 × 10^−8^) and CTD (twofold enrichment; *P* = 4.3 × 10^−4^) (*SI Appendix*, Table S15B), with enrichment limited to NOTCH1’s 36 EGF domains (*P* < 10^−10^). Each EGF domain has six cysteines which form three intradomain disulfide bonds that are critical domain structure (28). D-mis variants introducing or removing one cysteine, producing an odd number of cysteines, were enriched 8.9-fold (*P* < 10^−13^) and accounted for 41% (24/58) of the D-mis mutations in EGF domains (Table 2). These variants all leave an unpaired cysteine available for electrophilic attack. Cysteine-altering variants were highly clustered, with eight occurring in EGF domain #5 including two recurrent variants (250-fold enrichment, *P* < 10^−11^; Table 2 and Fig. 3 *A* and *B*), and 20 (83%) among the first 18 EGF domains. In contrast, there is only one cysteine mutation in the first 18 EGF domains in non-CTD/TOF probands. All these cysteine-altering variants had allele frequencies of zero in the BRAVO, ESP, and gnomAD databases (*SI Appendix*, Table S16). An additional missense variant altered asparagine 197 in EGF #5; this residue helps coordinate Ca^2+^ in this EGF domain; Ca^2+^ binding is known to be critical to NOTCH1 signaling (29).

**Table 2.** Enrichment of cysteine-altering NOTCH1 D-mis mutations in CHD probands with TOF or CTD, but not other cardiac phenotypes

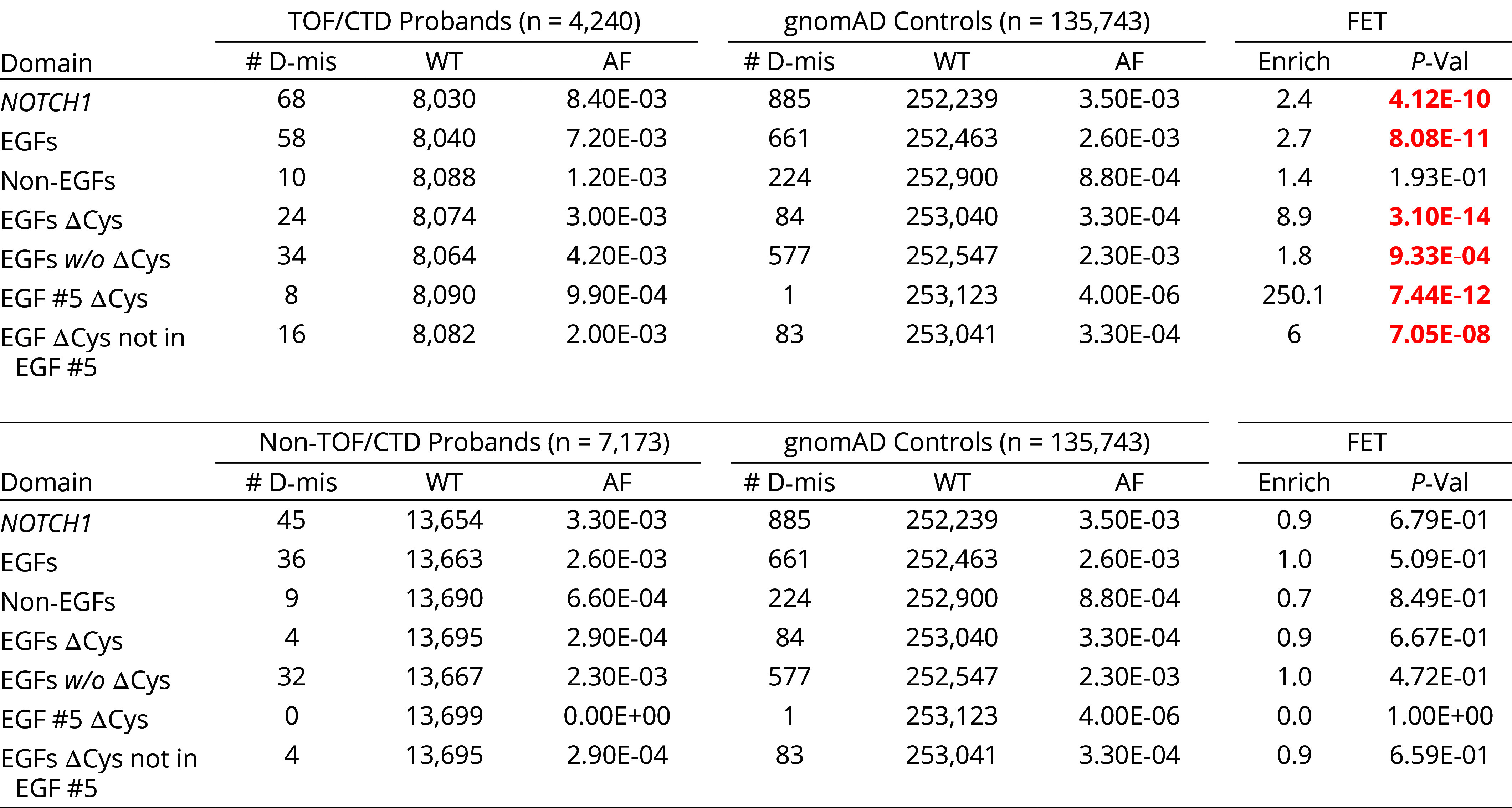

Case–control results are shown. Probands with CTD or TOF in the upper panel and probands with all other characterized cardiac phenotypes in the lower panel. ΔCys denotes cysteine-altering mutations. Domains from the PROSITE and PFAM databases. *P*-values significant in NOTCH1 subsets after Bonferroni multiple testing correction (*P* ≤ 0.05/6 = 8.3 × 10^−3^) are shown in red.

These clustered cysteine mutations are highly reminiscent of cysteine-altering variants in *NOTCH3* that cause cerebral autosomal dominant arteriopathy with subcortical infarcts and leukoencephalopathy (CADASIL). Notably, in trios, all 6 parental cysteine mutations in EGFs 1-18 were transmitted to probands with TOF or CTD. Among four kindreds with family history data, one transmitting parent had TOF while others had no CHD history.

### Clinical Characteristics of CHD Probands with Pathogenic Variants in Syndromic Genes.

Because clinically significant CHD was the sole requirement for study enrollment, and clinical phenotypes were recorded independently of mutation status, this cohort allowed unbiased estimates of the association of genotypes and specific phenotypes. We performed follow-up chart review of clinical features of probands with LOF variants in the most frequently mutated syndromic genes in our cohort—*CHD7* and *KMT2D,* and known GOF mutations in *BRAF*, *PTPN11*, *RAF1*, *RIT1*, *SHOC2*, and *SOS1* (and LOFs in *LZTR1*) causing Noonan syndrome and related RASopathies. Follow-up occurred 0 to 10 y after initial recruitment. Overall, among 133 probands with disease-causing variants in these genes, only 88 (66%) were clinically diagnosed with their respective syndromes, indicating incomplete sensitivity for the clinical diagnosis.

### *CHD7* and CHARGE Syndrome.

LOF variants in *CHD7* cause CHARGE syndrome, featuring coloboma, atresia choanae, slow growth and development, genital malformations, ear malformations, and congenital heart disease (31–33). Thirty-two probands with CHD7 LOFs had available medical records (Table 3). Twenty-three of these probands (72%) were clinically diagnosed with CHARGE.

**Table 3.** Phenotypes of probands with characteristic mutations causing syndromic CHD

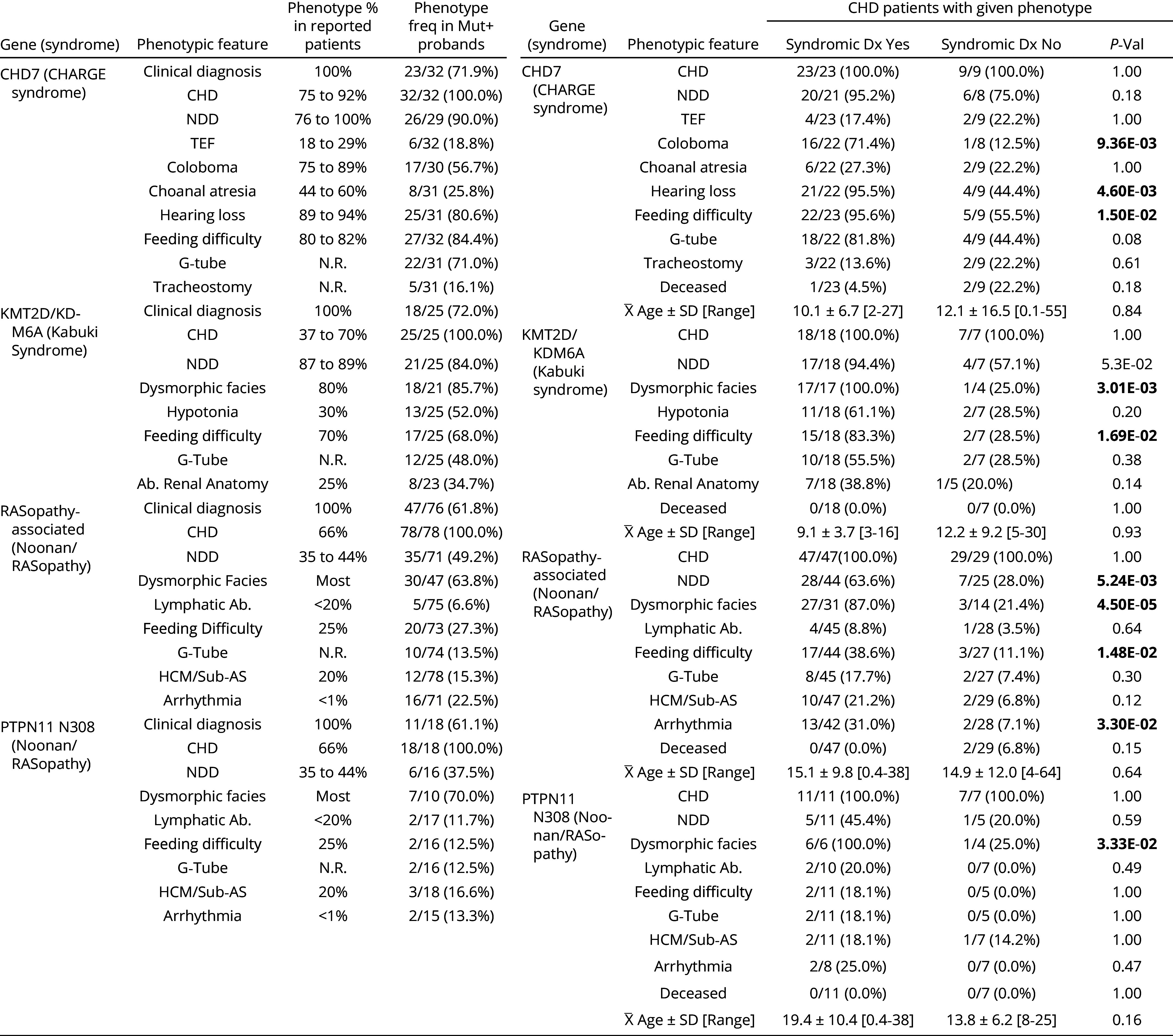

The left panel compares reported and observed frequencies of clinical phenotypes in probands with LOF mutations in *CHD7* and *KMT2D/KDM6A* and ClinVar+ mutations in *PTPN11*, *BRAF*, *RAF1*, *RIT1*, *SHOC2*, *SOS1 and LZTR1* genes together and N308 mutations in *PTPN11*. Citations for reported phenotype frequencies for CHARGE syndrome are (31–33); for, Kabuki syndrome (34–37); and Noonan/Rasopathy (10, 38, 39). HCM, hypertrophic cardiomyopathy. The right panel compares clinical phenotypes in CHD probands with pathogenic mutations that were or were not clinically diagnosed. *P*-values from two-tailed FET for binary traits, and Wilcoxon rank-sum test for continuous variables.

The frequency of characteristic CHARGE phenotypes in these probands and those reported in the literature showed that frequencies of coloboma (57% vs. 75 to 89% reported), choanal atresia (26% vs. 44 to 60% reported), and hearing loss (81% vs. 89 to 94% reported) were lower than previously estimated (Table 3) (31). Other frequencies were like those previously reported.

Overestimates of the frequency of syndromic features likely contributed to underdiagnosis of CHARGE. Thus, while 16 of 22 (71%) probands with coloboma were diagnosed with CHARGE, only 1 of 8 (13%) without coloboma was diagnosed. Similarly, 21 of 22 (96%) with hearing loss but only 4 of 9 (46%) without were diagnosed. The age at evaluation of those who were and were not diagnosed with CHARGE were similar (means of 10.1 and 12.1 y, respectively; *P* = 0.84), as was the distribution of LOF variants (*SI Appendix*, Fig. S12*A*).

### *KMT2D* and Kabuki Syndrome.

Kabuki syndrome features infantile hypotonia, NDD, palpebral fissures, and characteristic facial dysmorphism (34). Sixty to seventy-five percent of Kabuki probands have LOF DNMs in *KMT2D,* and 3 to 8% are due to LOF DNMs in *KDM6A* (40). Our cohort included 21 with LOF DNMs and 12 phase-unknown LOFs in *KMT2D* (*SI Appendix*, Table S19) and one LOF in *KDM6A.* CHD phenotypes were HLHS, CTD, and LVO, and all but two probands had EC, NDD, or both (*SI Appendix*, Table S19). Clinical details were available for 25 probands (*SI Appendix*, Table S20); 18 (72%) had a clinical diagnosis of Kabuki syndrome. The frequency of characteristic Kabuki phenotypes in our cohort was very similar to reported values, except increased hypotonia (52% vs. 30% reported) and abnormal renal anatomy (35% vs. 25% reported) (Table 3) (34–36). Only one of seven probands not diagnosed (Table 3), had dysmorphic facies reported, vs. 100% of those diagnosed with Kabuki (*P* = 3.0 × 10^−3^). Similarly, feeding difficulty was reported less frequently in those without a Kabuki diagnosis (29% vs. 83%; *P* = 1.7 × 10^−2^). There was no significant difference in age between diagnosed and undiagnosed probands (9.1 and 12.2 y, respectively; *P* = 0.93) and no difference in the distribution of LOF variants between these groups (*SI Appendix*, Fig. S12*B*).

There were also six D-mis DNMs in *KMT2D*, all clustered in its C-terminus, which includes domains found in lysine methyltransferases (*SI Appendix*, Fig. S13 and Table S21) (41); these DNMs were absent in BRAVO, ESP, and gnomAD databases. Two DNMs were identical R5351Q mutations. Four probands had HLHS (*SI Appendix*, Fig. S13). Of five probands with clinical data, only one was clinically diagnosed. One proband had NDD and thrombocytopenia, two were deceased, and three had feeding difficulty. One additional proband had a transmitted C-terminal variant, p.R5179H, identical to a DNM in a proband; this proband had HLHS and EC, consistent with a pathogenic variant. C-terminal missense variants in *KMT2D* have been reported in CHD and Kabuki syndrome, with variable expressivity of cardiac and EC features (37, 42, 43).

### RAS/MAPK Pathway and Noonan Syndrome.

Noonan syndrome and related RASopathies share distinctive facial features, NDD, short stature, lymphatic malformations, hematological abnormalities, and CHD (including congenital hypertrophic cardiomyopathy) (38, 39). They are caused by variants in the RAS/MAPK pathway, including GOF missense variants in activators of the pathway (*PTPN11, SOS1, RAF1, BRAF, SHOC2, RIT1*) and recessive LOF variants or recurrent dominant-negative missense variants in the 4th Kelch domain of *LTZR1*, an inhibitor of RAS signaling (44).

Ninety variants in these genes were identified as pathogenic in ClinVar (ClinVar+, *SI Appendix*, Table S22). Twenty-two were de novo, four were transmitted, and 64 were unphased (*SI Appendix*, Table S23). Seventy-eight probands had available medical records. Noonan syndrome/RASopathy-related phenotypes occurred near their reported frequencies (38, 45) (Table 3). Overall, 38% were not clinically diagnosed with Noonan/RASopathy (Table 3) (38). Only 21% of undiagnosed probands had dysmorphic facies reported, vs. 87% in those diagnosed (*P* = 4.5 × 10^−5^) and NDD was also less frequent in undiagnosed (28%) vs. diagnosed probands (64%) (*P* = 5.2 × 10^−3^) (Table 3). Notably, both deceased probands were from the undiagnosed group. Age at evaluation was similar (mean of 15.1 y in diagnosed vs. 14.9 y in undiagnosed probands; *P* = 0.64).

In contrast to the expected RASopathy-associated cardiac phenotypes (75 to 90% accounted for by PS, HCM, and ASD), probands had more ASD, less LVH, and more polyvalvular disease, BAV/aortic coarctation, and AVC (*SI Appendix*, Table S24A). Unexpectedly, arrhythmia requiring medical management or pacemaker/ICD was reported in 16 (23%) ClinVar+ probands (*SI Appendix*, Table S24B). Patients with arrhythmia were diagnosed with RASopathy more frequently than those without (87% vs. 52%, *P* = 1.9 × 10^−2^), were older (22.7 vs. 13.5 y, *P* = 0.046) and were more likely to have LVH (31% vs. 13%, *P* = 0.13).

A clinical diagnosis was made more frequently in probands with variants in *RAF1, RIT1,* and *SOS1* (collectively 15/18; 83% diagnosed) vs. those with variants in *LZTR1* (0/5 diagnosed) or *PTPN11* (29/49; 59% diagnosed) (*SI Appendix*, Table S25).

Variants in *PTPN11* included many at known mutational hotspots (*SI Appendix*, Fig. S12*C*). Among 18 probands with an N308 variant (46), 11 were diagnosed with Noonan/RASopathy and seven were not. Dysmorphic facies were present in all diagnosed probands but only 25% of undiagnosed (*P* = 0.03) (Table 3) and ages were similar (mean 19.4 and 13.8 y, respectively; *P* = 0.16).

The absence of a clinical diagnosis of these syndromes was correlated with the absence of characteristic clinical features that were variable among probands harboring disease-causing variants.

## Discussion

This study represents, to our knowledge, the largest investigation to date of the impact of rare variants on structural CHD. Use of MIPseq and WES has increased the number of probands with genomic data fourfold compared with our previous study (10) and enabled substantial progress in gene discovery. The robust MIPseq gene panel captured over 1.4 Mb of genomic DNA sequence from 248 genes with high completeness and precision. MIPseq can provide a low-cost platform for discovery or clinical genetic diagnosis for diseases featuring high locus heterogeneity, though continued reductions in sequencing costs will narrow the potential advantages of this method compared with whole exome or whole genome sequencing.

The results increase the number of genes statistically implicated in monoallelic CHD from this cohort to 60. Of the 13 genes not previously implicated in CHD, 10 had 2 or more damaging DNMs in the training set and 2 were nominated based on suggestion from prior biology. Of 3 genes not previously implicated in human CHD, 1 had >2 DNMs in the training set and 2 were high pLI chromatin genes. Of note, 9 genes in the gene panel that are not currently significant have 2 damaging DNMs, including chromatin genes KDM5A, KMT2A, KMT2C, NSD2, SETD5, and CREBBP, suggesting some will likely become significant with further study.

Damaging DNMs in these 60 genes are found in 5.1% of probands, accounting for 57% of the DNM signal (*SI Appendix*, Table S10A). TDT implicated transmitted variants in 5.4% of probands, a marked increase from previous estimates (*SI Appendix*, Table S10C). Collectively, 10.1% of probands had at least one disease-related variant.

Monte Carlo simulation provided a maximum likelihood estimate that 297 genes (95% CI: 169.4 to 424.6) contribute to CHD by DNM demonstrating that much remains to be discovered. From the 248 gene panel, the current results predict that 111 will ultimately prove to be significant (*Materials and Methods*).

The DNM frequency is not distributed normally across these CHD genes, even after adjusting for gene mutability. Damaging DNMs in just four genes (*KMT2D*, *CHD7*, *PTPN11,* and *NOTCH1*) account for a third of these DNMs (69/208) (vs. expected 8%; the Shapiro–Wilk test rejected a normal distribution, *P* < 10^−11^). The high frequency of GOF DNMs in *PTPN11* and some other RASopathy genes can be explained by positive selection of these variants in the paternal germ cell lineage (47, 48); however, germline selection has not been reported for *KMT2D, CHD7,* and *NOTCH1* (49). Another explanation for the high prevalence of DNM in these genes could be much greater survival to birth among embryos with mutations in these genes. This could explain the very long tail on the curve relating sample size to the discovery of new disease genes.

Some of the newly implicated genes link to known biology. *CUL3* (pLI = 1) is a ubiquitin ligase component that partners with many proteins that target specific proteins for ubiquitylation and degradation (50). One of these is *LZTR1*, a known RASopathy gene that targets proteins in the RAS pathway for degradation, Similarly, *LHX2* (pLI = 0.99), encodes a transcriptional activator; mouse knockouts produce CHD (51). Both probands with damaging DNMs had aortic and mitral atresia. *AHNAK* encodes a heart scaffolding protein that regulates L-type calcium channels (52). *SVEP1* encodes an extracellular adhesion protein that, in zebrafish, was responsive to blood flow and interacted with the VEGF pathway (53). *NR6A1*, encoding a nuclear receptor, is implicated in vascular smooth muscle cell migration (54) and was upregulated in mice with CHD born of diabetic mothers (55). *CLUH* (pLI = 0.99), encodes an mRNA-binding protein involved in intracellular distribution and biogenesis of mitochondria, linked to cardiac hypertrophy (56). Further work will be required to establish the role of these and other genes newly implicated in human CHD.

Consistent with their shared role in cardiac development, the 60 core CHD genes are significantly more connected than expected by STRING analysis (*P* < 1.0 × 10^−16^), and many of the genes newly implicated in human CHD are connected within the network (*SI Appendix*, Fig. S14) (57).

Transmitted D-mis mutations in MYH6 proved to play a surprisingly large role, contributing to 2 to 3% of ASD, HLHS, and LVO, and nearly 1% of the entire cohort studied. The estimated effect sizes of these variants from TDT were large. Nonetheless, only 3 of 32 transmitting parents and two siblings of probands had a clinical diagnosis of CHD.

Similarly, all 11 parental LOFs in *NOTCH1* were transmitted to probands, as were all six parental cysteine-altering mutations among the first 18 EGF domains of *NOTCH1*, but only 4/15 transmitting parents from these trios with available medical histories had clinical CHD.

The significant variability in CHD phenotypes resulting from LOF mutations in 12 of these genes is striking. Ten had pLI > 0.9; nine of these showed variable EC and NDD phenotypes, consistent with dosage sensitivity for multiple developmental processes. Explanations for the variable expressivity could be explained by common variant modifiers, environmental influences, stochastic events, or subclinical CHD, as has been described for LOF variants in *NOTCH1* (58). Genome-wide association studies in this cohort would be useful in assessing whether variable expressivity can be explained by common modifier alleles.

The results increased the number of significantly mutated chromatin genes to 10. These are predominantly de novo LOFs, with few transmitted variants. These probands are enriched for EC phenotypes, reflecting the role of chromatin genes in many cell types. Maximum likelihood estimates that 28 chromatin genes contribute to CHD, with some identified in other cohorts (11).

Cysteine-altering variants clustered in *NOTCH1* n-terminal EGF domains, were highly enriched in probands with CTD and TOF. These variants are analogous to mutations in *NOTCH3* that cause CADASIL (59). Mutant NOTCH3 extracellular domains accumulate in the basement membrane of small arteries, arterioles, and capillaries of the brain and skin, consistent with protein misfolding and/or electrophile attack of the unpaired cysteines (60).

Pairing genetic data with systematically collected cardiac and EC phenotypes enabled estimates of the frequency of phenotypes associated with each gene. For example, NDD was uncommon in probands with damaging variants in *MYH6* variants but extremely common in those with LOF variants in *CHD7* and *KMT2D*. The ability to assess future risk of NDD at birth provides the potential for early intervention.

Limitations to the study include the challenge of accurately assessing from whether rare missense variants are deleterious or benign. Also, assessment of NDD phenotypes by questionnaire in very young probands may underestimate the ultimate frequency of NDD. Nonetheless, genes that were/were not statistically associated with NDD or EC showed significant differences in EC gene brain expression, a result not expected by chance (Fig. 2*B* and *SI Appendix*, Fig. S9*B*). Also, for *CHD7*, *KMT2D,* and Noonan/RASopathy genes (Table 3), systematic chart review up to 10 y after initial ascertainment gave similar estimates of NDD frequency as obtained by questionnaire at ascertainment. The age distributions of probands at enrollment are shown in *SI Appendix*, Fig. S15. The percentage of probands with NDD was consistently 15 to 20% of probands through age 25. The percentage subsequently declined with increasing age, potentially due to selection for survival and/or ascertainment bias.

With variable expressivity, ascertainment will influence the frequency of specific phenotypes observed. For example, a prior study ascertaining for congenital hydrocephalus identified 5 probands with damaging DNMs or transmitted LOFs in *SMARCC1* (61). In contrast, the PCGC cohort had 10 CHD probands with *SMARCC1* LOFs, none with NDD. While a few genes (CHD7, KMT2D, PTPN11, NOTCH1) have enough probands with pathogenic mutations to have reasonable estimates of the frequency of NDD, there are insufficient data to exclude NDD or accurately estimate its frequency for many genes. Much larger cohorts will be needed to both extend CHD gene discovery and define clinical variability in phenotypes arising from mutation of each CHD gene.

Collectively, rare monoallelic variants account for at least 40% of CHD: 25% from large CNVs and aneuploidy (7), 9% via damaging DNMs, at least 5% from transmitted variants as reported herein, and 2% from recessive inheritance (62). Studies have also implicated rare noncoding variants (63, 64) and common variants with small effects (65, 66), but these have been limited by small sample size and inability to link specific variants to CHD. Nongenetic factors including pregestational maternal diabetes, hypertension, alcohol use, and others account for another 10 to 14% of CHD (6, 67); the mechanisms of these effects are unknown. Given the large role of haploinsufficiency for genes with high pLI, stochastic effects that reduce expression of these genes at critical times in particular cell types could explain phenocopies in the absence of mutation.

The results herein demonstrate the significantly greater precision of molecular diagnosis of CHD to establish an early diagnosis for the syndromic genes frequently mutated in CHD. These patients are at variable risk for NDD, hearing loss, arrhythmias, and other disorders that can impact clinical management and outcome. The clinical implications for prevention and mitigation provided by early molecular diagnosis are increasingly apparent and support the routine use of genomic analysis in evaluation of CHD.

## Materials and Methods

### Ascertainment and Clinical Characteristics of Study Population.

11,555 probands and 7,774 parents, comprising 3,887 trios and 7,668 singleton probands recruited to the Congenital Heart Disease Network Study of the Pediatric Cardiac Genomics Consortium were investigated (CHD GENES: ClinicalTrials.gov identifier NCT01196182) (68). All participants or their parents provided informed consent using protocols that were reviewed and approved by institutional review boards of Boston’s Children’s Hospital, Brigham and Women’s Hospital, Children’s Hospital of Los Angeles, Children’s Hospital of Philadelphia, Columbia University Medical Center, Great Ormond Street Hospital, Icahn School of Medicine at Mount Sinai, Rochester School of Medicine and Dentistry, Steven and Alexandra Cohen Children’s Medical Center of New York, University of California-San Francisco School of Medicine, University of Utah School of Medicine, Stanford University School of Medicine and Yale School of Medicine. All data are stripped of identifiers and labeled with a study number. Classification of CHD and EC features were as previously described (68) (*SI Appendix*, Table S3). Further evaluation of probands found to have mutations associated with CHARGE syndrome, Kabuki syndrome, or Noonan syndrome/RASopathies was done through focused chart review.

### Molecular Inversion Probe Design, Sequencing, and Processing.

A MIPseq panel of 248 genes were selected using data from our earlier study of 1,213 CHD trios (9) (*SI Appendix*). Probes were designed using the MIPGen algorithm applying default parameters (69) with modifications, including pooling of sets of probes of similar G-C content, iterative rounds of development to replace ineffective probes, and balancing of pool inputs into sequencing reaction to produce similar levels of sequence coverage. Gap filling reactions spanned ~250 bp of genomic sequence and adjacent probes overlapped by at least 30 bp. Design included a random 6 bp unique molecular identifier barcode added to the extension arm of each probe, permitting recognition and exclusion of PCR duplicates. Probe targets covered all coding regions plus at least 12 intronic bases flanking each exon (70). Probes were synthesized by Integrated DNA Technologies at a scale sufficient to genotype 50,000 samples. The probe design file in Dataset S2. Library preparation and sequencing of MIPseq samples was modified from previously published methods (71) and fully described in *SI Appendix*. MIPseq and WES was performed on the Illumina Platform at the Yale Center for Genome Analysis (YCGA). The total production cost of research MIPseq for each sample was ~$25.50 per sample.

### Variant Calling.

Variants were called using GATK v3.7 (72) using default parameters, disabling of variant quality score recalibration due to lack of training SNPs in the MIPseq panel; variants were also called with Freebayes v1.3.2 (https://github.com/ekg/freebayes) using default parameters. The union of these variant calls was annotated using ANNOVAR (73), multiallelic sites were split with BCFtools (74), and insertion-deletion variants were left-aligned with BCFtools (*SI Appendix*). Sensitivity and specificity for calling rare variants from the MIPseq pipeline was established by comparing MIPseq calls from Genome in a Bottle sample NA12878 compared to gold-standard variants for this sample (*SI Appendix*, Fig. S6*A*) (17). We also compared MIPseq and WES of 170 PCGC CHD samples (*SI Appendix*, Fig. S6*B*). Variants from WES of PCGC samples were called as described previously (10).

To validate candidate variants called by the MIPseq pipeline in new CHD probands, we performed PCR amplification using custom primers followed by Sanger sequencing of 59 very rare variants comprising a wide range of mutation types (*SI Appendix*, Table S1).

### Cohort Sequencing.

All MIPseq and WES was performed on the Illumina Platform at YCGA. The study cohort contains 11,555 CHD probands, 5,929 with MIPseq, and 5,626 with WES, including 1,739 singletons and 3,887 probands with both parents (7774).

The control cohort comprises 133,743 samples with WES or WGS from the gnomAD database after exclusion of samples that are also present in the TopMed database, which is included in the BRAVO variant database (20). Parental and sample-level data for gnomAD samples were not available.

### Annotation of Variants.

Variants were annotated for the most severe functional consequence among all potential isoforms using ANNOVAR and Meta-SVM from gnomAD v2.1.1 (20) in both cases and controls as previously described (10).

### Analysis of DNMs in Trios.

DNMs in trios were identified using TrioDeNovo (75) and filtered as previously described (10). All candidate variants were visually inspected in silico in the proband and both parents.

Enrichment of DNMs in probands was calculated from the observed frequency compared to the mutability-based expectation as previously described (10). The genomic inflation per thousand samples (λ_1000_) was calculated (76). To determine the enrichment of DNMs in sets of multiple genes, the observed and expected counts were summed across all genes included in a given set. We estimated the number of genes expected to have >1 DNM in a cohort of 3,887 trios as described previously (10).

### Identification and Analysis of Very Rare TUVs in Probands and Controls.

We filtered for variants with a MAF ≤ 10^−5^ in both BRAVO (18) and EVS (77) databases. We also removed variants that had a within-cohort MAF ≤ 1.3 × 10^−4^ independently for CHD cases and gnomAD controls. BRAVO variants were lifted over from hg38 to hg19 using LiftOver (78). In gnomAD controls, the 100 most frequent very rare variants were visualized in silico using at least three representative variants from the online gnomAD browser (20); those found to be false positives were removed. We further harmonized cases and controls by removing exons or flanking splice regions that were not targeted for capture by all platforms.

To calculate enrichment of TUVs in cases, we compared the frequency of alleles of each class (i.e., synonymous, T-mis, D-mis, LOF) in cases and controls and calculated *P*-values using a one-tailed Fisher’s exact test (FET).

### Meta-Analysis of DNMs and Very Rare TUVs.

Utilizing all CHD probands, we performed a gene-level meta-analysis on all 248 panel genes using the Poisson *P*-values from DNMs in CHD trios and the FET *P*-values from the case–control. We used the Joint-Local False Discovery Rate (“JL-FDR”) method (21) to generate meta-analysis *P*-values and FDR statistics that account for the number of genes tested. To perform the meta-analysis of DNMs and very rare TUVs at a phenotype-level on the 60 genes identified as significantly enriched in all probands, we repeated the meta-analysis procedure using the JL-FDR method to generate *P*-values and FDR statistics that accounted for the eight cardiac and four NDD/EC phenotypes tested.

When performing meta-analysis of DNMs and TUVs on individual genes, we used Fisher’s Method to calculate the meta-analysis *P*-value and applied a Bonferroni correction to determine the threshold for significance. When performing multiple testing correction on the *P*-values from the Poisson test or FET independently, the Benjamini–Hochberg procedure was used to generate FDR statistics.

### Transmission Disequilibrium Test in Rare Variants from WES Trios.

For the 60 significant genes in the MIPseq panel, transmission disequilibrium test (TDT) was performed (79), for rare damaging variants in the 3,887 WES trios. The significance of transmission disequilibrium was calculated by chi-square tests and the genotypic risk ratio was calculated (22).

### Estimating the Total Number of Risk Genes in MIPseq Panel.

We estimated the total number of CHD risk genes in the 248 gene MIPseq panel using the JL-FDR framework (21), fitting a two-component bivariate normal mixture distribution with an EM-algorithm (80) (*SI Appendix*).

### Calculation of Proportion of Cases Attributable to DNMs and Very Rare TUVs.

We calculated the fraction of probands having a likely damaging de novo variant of a tested class from the fraction of probands with such a variant minus the expected fraction from mutability (10), and similarly calculated this from the difference in the observed frequency of TUVs in probands and controls.

## Supplementary Material

Appendix 01 (PDF)

Dataset S01 (XLSX)

Dataset S02 (XLSX)

Dataset S03 (XLSX)

Dataset S04 (XLSX)

Dataset S05 (XLSX)

Dataset S06 (XLSX)

Dataset S07 (XLSX)

Dataset S08 (XLSX)

## Data Availability

Phenotype and DNA sequence data have been deposited in the NIH National Heart Lung and Blood BioData Catalyst https://biodatacatalyst.nhlbi.nih.gov under “congenital heart disease” phs001194 (81) and the main PCGC substudy phs000571 (82).
