# Supplementary material for: Genomic analysis of 11,555 probands identifies 60 dominant congenital heart disease genes
Source: Proc Natl Acad Sci U S A. 2025 Mar 24;122(13):e2420343122. doi: 10.1073/pnas.2420343122 (PMC12002227; doi:10.1073/pnas.2420343122)
Supplement: Supplementary file 1 — Appendix 01 (PDF) [file pnas.2420343122.sapp.pdf]

Supporting Information for

Genomic analysis of 11,555 probands identifies 60 dominant congenital heart disease genes

Michael C. Sierant[1,2], Sheng Chih Jin[2,3], Kaya Bilguvar[1,4,5], Sarah U. Morton[6], Weilai Dong[1,2], Wei Jiang[7], Ziyu Lu[8], Boyang Li[7], Francesc López-Giráldez[4], Irina Tikhonova[4], Xue Zeng[1,2], Qiongshi Lu[9], Jungmin Choi[1,2,10], Junhui Zhang[1], Carol Nelson-Williams[1], James R. Knight[4], Hongyu Zhao[1,7], Junyue Cao[8], Shrikant Mane[4], Stanley C. Sedore[11,12], Peter J. Gruber[13], Monkol Lek[1], Elizabeth Goldmuntz[14], John Deanfield[15], Alessandro Giardini[16], Seema Mital[17], Mark Russell[18], J. William Gaynor[19], Eileen King[20], Michael Wagner[21], Deepak Srivastava[22], Yufeng Shen[23], Daniel Bernstein[24], George A. Porter Jr[25], Jane W. Newburger[26], Jonathan G. Seidman[27], Amy E. Roberts[26], Mark Yandell[28], H Joseph Yost[28,29], Martin Tristani-Firouzi[30], Richard Kim[31], Wendy K. Chung[32], Bruce D. Gelb[33], Christine E. Seidman[34], Martina Brueckner[1,11*], Richard P. Lifton[2*]

1. Department of Genetics, Yale School of Medicine, New Haven, CT, 06510, USA.
2. Laboratory of Human Genetics and Genomics, The Rockefeller University, New York, NY, 10065, USA.
3. Department of Genetics, Washington University School of Medicine, St. Louis, MO, 63110, USA.; Department of Pediatrics, Washington University School of Medicine, St. Louis, MO, 63110, USA.
4. Yale Center for Genome Analysis, Yale University, New Haven, CT, 06516, USA.
5. Department of Neurosurgery, Yale School of Medicine, New Haven, CT, 06510, USA.; Yale Program on Neurogenetics, Yale School of Medicine, New Haven, CT, 06510, USA.; Department of Medical Genetics, School of Medicine, Acibadem University, Istanbul, Turkey.; Department of Translational Medicine, Health Sciences Institute, Acibadem University, Istanbul, Turkey.
6. Division of Newborn Medicine, Department of Pediatrics, Boston Children's Hospital, Boston, MA 02115, USA, Manton Center for Orphan Disease Research, Boston Children's Hospital, Boston, MA 02115, USA, Broad Institute of Massachusetts Institute of Technology and Harvard, Boston, MA 02142, USA.
7. Department of Biostatistics, Yale School of Public Health, New Haven, CT, 06510, USA.
8. Laboratory of Single-cell Genomics and Population Dynamics, The Rockefeller University, New York, NY, 10065, USA.

9. Department of Biostatistics & Medical Informatics, University of Wisconsin, Madison, WI, 53706, USA.
10. Department of Biomedical Sciences, Korea University College of Medicine, Seoul, South Korea.
11. Department of Pediatrics, Yale School of Medicine, New Haven, CT, 06510, USA.
12. Department of Pediatrics, Michigan State University College of Human Medicine, Grand Rapids, MI, 48824, USA.
13. Department of Surgery, Yale University School of Medicine, New Haven, CT, 06510, USA.
14. Division of Cardiology, Children's Hospital of Philadelphia, Department of Pediatrics, Perelman School of Medicine, University of Pennsylvania, PA, 19104, USA.
15. Institute of Cardiovascular Science, University College London, London WC1E 6BT, United Kingdom
16. Pediatric Cardiology, Great Ormond Street Hospital, London WC1N 3JH, United Kingdom.
17. Division of Cardiology, Department of Pediatrics, The Hospital for Sick Children, University of Toronto, Toronto, ON M5G1X8, Canada.
18. Department of Pediatrics and Communicable Diseases, University of Michigan, Ann Arbor, MI, 48109, USA.
19. Division of Cardiothoracic Surgery, Children's Hospital of Philadelphia, Philadelphia, PA, 19104, USA.
20. Department of Pediatrics, University of Cincinnati, Cincinnati, OH, 45229, USA.
21. Division of Biomedical Informatics, Cincinnati Children's Hospital Medical Center, Cincinnati, OH 45229, USA, Division of Biostatistics and Epidemiology, Cincinnati Children's Hospital Medical Center, Cincinnati, OH 45229, USA.
22. Gladstone Institute of Cardiovascular Disease and University of California San Francisco, San Francisco, CA, 94158, USA.
23. Department of Systems Biology and Department of Biomedical Informatics, Columbia University Irving Medical Center, New York, NY, 10032, USA.
24. Department of Pediatrics, Cardiology, Stanford University, Stanford, CA, 94304, USA.
25. Department of Pediatrics, University of Rochester Medical Center, The School of Medicine and Dentistry, Rochester, NY, 14642, USA.
26. Department of Cardiology, Boston Children's Hospital, Department of Pediatrics, Harvard Medical School, Boston, MA, 02115, USA.
27. Department of Genetics, Harvard Medical School, Boston, MA, 02115, USA.
28. Department of Human Genetics, University of Utah and School of Medicine, Salt Lake City, UT, 84112, USA.

29. The Catholic University of America, Washington, DC 20064, USA.
30. Division of Pediatric Cardiology, University of Utah, Salt Lake City, UT, 84112, USA.
31. Pediatric Cardiac Surgery, Smidt Heart Institute, Cedars-Sinai Medical Center, Los Angeles, CA, 90048, USA.
32. Department of Pediatrics, Boston Children's Hospital, Harvard Medical School, Boston, MA 02115, USA, Departments of Pediatrics and Medicine, Columbia University Medical Center, New York, NY, 10032, USA.
33. Mindich Child Health and Development Institute and Department of Pediatrics, Icahn School of Medicine at Mount Sinai, New York, NY, 10029, USA.
34. Cardiovascular Division, Brigham and Women's Hospital, Boston, MA, 02115, USA, Howard Hughes Medical Institute, Chevy Chase, MD, 20815, USA.
* Corresponding Author
Email: rickl@rockefeller.edu or martina.brueckner@yale.edu

This PDF file includes:
Supporting text
Figures S1 to S15
Tables S1 to S25
Legends for Datasets S1 to S8
SI References
Other supporting materials for this manuscript include the following:
Datasets S1 to S8

Supporting Information Text

Material and Methods

Ascertainment and clinical characteristics of study population

11,555 probands and 7,774 parents were recruited to the Congenital Heart Disease Network Study of the Pediatric Cardiac Genomics Consortium (CHD GENES: ClinicalTrials.gov identifier NCT01196182)(1). Written informed consent for genetic studies was obtained from all participants. Only probands with structural CHD (excluding PDA associated with prematurity and pulmonary stenosis associated with twin-twin transfusion) were included. The cardiac diagnoses were based on the review of imaging and operative reports and were entered as Fyler codes based on the International Pediatric and Congenital Cardiac Codes (http://www.ipccc.net/). Samples were excluded if from probands with one of the following genetic syndromes: Cat Eye, DiGeorge/Velo-Cardio-Facial/Cayler cardiofacial, Jacobsen, trisomy 21, Turner, and Williams syndrome. Further, probands with an identified variant in a known syndromic gene outside the Molecular Inversion Probe Sequencing (MIPseq) gene panel were excluded from recruitment. We thank the following people for outstanding contributions to patient recruitment: P. Agrawal, A. Julian, M. Mac Neal, Y. Mendez, T. Mendiz-Ramdeen and C. Mintz (Icahn School of Medicine at Mount Sinai); N. Cross (Yale School of Medicine); J. Ellashek and N. Tran (Children's Hospital of Los Angeles); B. McDonough, J. Geva and M. Borensztein (Harvard Medical School), K. Flack, L. Panesar and N. Taylor (University College London); E. Taillie (University of Rochester School of Medicine and Dentistry); S. Edman, J. Garbarini, J. Tusi and S. Woyciechowski (Children's Hospital of Philadelphia); D. Awad, C. Breton, K. Celia, C. Duarte, D. Etwaru, N. Fishman, E. Griffith, M. Kaspakoval, J. Kline, R. Korsin, A. Lanz, E. Marquez, D. Queen, A. Rodriguez, J. Rose, J.K. Sond, D. Warburton, A. Wilpers and R. Yee (Columbia University Irving Medical Center).

Cardiac lesions were classified into eight major groups (2). '**Conotruncal Defects' (CTD**) included double-outlet right ventricle (DORV), truncus arteriosus, membranous ventricular septal defects (VSD), aortic arch abnormalities (except isolated coarctation of the aorta or hypoplastic aorta without VSD), pulmonary stenosis or atresia, or other related abnormalities; **Tetralogy of Fallot (TOF)** was categorized separately. **'Laterality defects' (LAT)** included situs abnormalities (*e.g.*, dextrocardia, situs inversus), left or right isomerism (LAI or RAI, respectively), L-Transposition of the great arteries (L-TGA) or D-Transposition of the great arteries (D-TGA); isomerism of other organs was not considered a separate extracardiac malformation. **'Left ventricular outflow tract obstruction' (LVO)** included aortic

stenosis/bicuspid aortic valve (AS/BAV), coarctation of the aorta (CoA), or other related abnormalities (without associated membranous VSD or DORV). '**Hypoplastic left heart syndrome' (HLHS)** was a separate category based on the primary cardiac lesion being underdeveloped left-sided cardiac structures and only a single functioning ventricle. **'Atrial septal defects' (ASD)** include only isolated ASD. **'Atrioventricular canal defects' (AVC)** included all atrioventricular-canal type defects except those associated with Laterality Defects. **'Other abnormalities' (OTH)** was assigned to cases with additional categories of cardiac lesions; including double inlet left ventricle (DILV), anomalous pulmonary veins, coronary artery abnormalities, tricuspid valve atresia (TA), or Ebstein's Anomaly; complex compound cardiac lesions that did not fall into any of the previously mentioned definitions were also included in this group. 'Unknown' (UNK) was assigned to cases with inadequate data on specific cardiac lesions.

Neurodevelopmental status in all PCGC probands who were at least one year of age at the time of enrollment was based on parental responses to a questionnaire obtained at the time of enrollment. Probands over 18 years of age filled out the questionnaire themselves. Probands were categorized as having NDD if their parents (or older probands) answered "Yes" to having been told by a physician that the proband had one or more of the following diagnoses: developmental delay, learning disability, intellectual disability, or autism, and as not having NDD if none of these diagnoses had been made. Probands younger than 1 year at the time of recruitment and those without a clinical assessment were classified "unknown" for NDD status. Among 9,952 PCGC probands included in the study, 3,232 were under 1 year of age at enrollment; 6,720 probands were one year or older were given the questionnaire; 6,613 (98%) completed the questionnaire. Extracardiac Anomaly (EC) status was obtained by chart review at the time of enrollment for the presence of any congenital structural defect outside of the heart; probands without clinical evaluation/assessment were defined as "unknown" for EC status. Subsequently, probands were grouped into four categories based on their NDD and EC status: (1) "isolated CHD" status was assigned to probands without EC or NDD and that were not assigned 'unknown' for either; (2) "EC" status was assigned to probands with presence of EC and without NDD; (3) "NDD" status was assigned to probands with presence of NDD without NDD; and (4) "unknown" status was assigned to probands that had an 'unknown' assessment for either EC or NDD.

Further evaluation of phenotypes of probands with mutations linked to CHARGE, Kabuki, and RASopathy-associated syndromes was done through focused chart review that included updated cardiac, neurodevelopmental, growth and syndrome-specific traits as well as whether or not the probands had been clinically diagnosed with appropriate syndrome. For probands with characteristic mutations for CHARGE syndrome, the presence of NDD was scored as the presence of one or more of the following:

developmental delay and intellectual disability. For probands with characteristic mutations for Kabuki Syndrome, NDD was scored as the presence of one or more of developmental delay, intellectual disability and neurobehavioral diagnosis. For probands with characteristic RASopathy mutations, NDD was scored as the presence or absence of one or more of developmental delay, Intellectual disability, attention deficit disorder and learning disability.

Additionally, proband and family ancestry was determined based on PCA analysis when possible and self-report when necessary. To assess the reliability of self-reported ancestry, we compared the result of ancestry determined by PCA versus self-report on 2,246 samples and found 97.8% were concordant for European ancestry. Sex was determined through PLINK (3) or by self-report.

Specific gene selection criteria

The MIPseq panel includes 20 genes with two or more DNMs from previous analyses, nine chromatin modifying genes with previously identified DNMs and 132 others. Chromatin modifying genes were defined using the GO terms 'chromatin organization' (GO:0016568), 'covalent chromatin modification' (GO:0016569), and 'Histone Modification' (GO:0016570) (2, 4). Among these genes, we selected those with a probability for loss of function variant intolerance (pLI) > 0.9 based on annotation from gnomADv2 (5). Additionally, we curated a list of 46 'known' or candidate CHD genes from the literature. Genes were included if they had associated CHD through a monoallelic mechanism in OMIM, or there was literature evidence of association between a candidate gene and CHD beyond isolated case reports. Among genes primarily included for chromatin modifying activity, 20 genes were identified as known or candidate CHD genes: *ARID1A*, *ASXL1*, *BCOR*, *CHD4*, *CHD7*, *CREBBP*, *EHMT1*, *EP300*, *KANSL1*, *KAT6B*, *KDM5B*, *KDM6A*, *NSD1*, *PRDM6*, *SALL1*, SMAD4, *SMARCA4*, and *SMARCB1*. 41 other genes were included based on earlier data. A full list of panel genes annotated with GO term and intolerance scores is provided in **Extended Dataset DS1**.

Molecular Inversion Probe design

Probes were designed using the MIPGen algorithm applying default parameters as described previously (6), except for those that follow. For probe quality filtering, the "mixed" method was used to select probes with an SVR (support vector regression) score greater than 1.4 and a logistic score greater than 0.7 unless otherwise necessary to capture a targeted region. A minimum capture (gap) size of 120bp and maximum of 250 was selected. Minimum probe overlap was set to 30bp. For single nucleotide polymorphism (SNP) avoidance, we used dbSNP b149 on genome build GRCh37p13. Design included a 6bp UMI barcode added to the extension arm. This UMI allows for the identification of 6^4 (=1,269) unique target capture

events for each probe, permitting identification and computational removal of PCR duplicates produced during target amplification. The input target genomic regions used were coding exons from the merged set of all 'NCBI RefSeq Curated' isoforms for the 248 panel genes on hg38, extended to include at least 12 intronic bases 5' and 3' to each exon in order to capture variants in splice donor and acceptor sites (7). Probes were synthesized by Integrated DNA Technologies (IDT). The probe design file for 10,154 probes from these 248 genes is available as **Extended Dataset DS2.**

Molecular Inversion Probe library preparation, sequencing protocol, and processing pipeline

We generated a library preparation and sequencing protocol for MIPseq, modified from previously published methods (8). The protocol is briefly summarized as follows with an unabridged version below. Prior to library capture, probes were stratified into quartiles of G-C content and each pool was independently phosphorylated using T4 polynucleotide kinase. Genomic DNA from whole peripheral blood or buccal swab was purified and quantified by PicoGreen assay. For each sample, 300ng of genomic DNA at a concentration of 15ng/ul was used as template with phosphorylated probes from sets SP1 or SP2 added to a concentration of 80uM. Gap-filling was performed with Hemo KlenTaq DNA polymerase (Taq DNA Polymerase I with N-terminal 280 amino acid truncation and additional amino acid changes to increase resistance to inhibitors present in whole blood) and the product was made into a closed circle by DNA ligase. Template DNA and open probes were digested with exonuclease I and III to isolate circularized probes which were subsequently amplified via PCR using primers complementary to invariable sequences in the probe backbone that include Illumina sequencing adapters.

To isolate probes that successfully captured DNA target, amplified DNA was quantified by PicoGreen assay, subjected to agarose gel electrophoresis, and the resulting 330bp band (approximate size of PCR product that includes hybridization arms plus captured target DNA) was extracted for gel purification. Purified DNA was quantified on a DNA 1000 Bioanalyzer chip. Sequencing was performed by Illumina Next-Generation sequencing where samples are multiplexed, loaded onto the flow cell in bulk, and sequenced to a target number of paired-end reads per lane on an Illumina HiSeq 2500, Illumina HiSeq 4000, or Illumina NovaSeq platforms. Sequencing was performed at the Yale Center for Genome Analysis (YCGA), without Clinical Laboratory Improvement Amendments (CLIA) certification.

Raw sequencing reads, following Illumina adapter demultiplexing, were first processed using an in-house pipeline to trim probe extension and ligation arm sequences and to facilitate UMI-mediated PCR duplicate removal. This is accomplished by first matching each paired-end read to a probe from the probe library generated during initial probe design using the extension and ligation arm sequence; we allowed

up to 1bp of mismatch anywhere in the extension or ligation sequences since we had determined that all extension and ligation arm sequences in this panel differ at 3 or more positions. Next, the 6bp UMIs were then removed from the extension arm of each paired read. Lastly, all paired reads that are matched to the same probe with identical UMIs represent PCR duplicates and, except for the paired-read with the greatest median base quality score, were subsequently removed from further analysis. Paired-reads which contain an UMI with any no-call ("N") bases or those which do not match probes in the design database were discarded. This read processing ultimately produces paired reads which represent unique target captures.

Four pools of MIPseq probes were assembled using equimolar amounts of each probe from the 1st, 2nd, 3rd and 4th quartile of probe G-C content. Probes comprising each pool were tested on genomic DNA as described above and the number of independent reads from each probe was determined. Probes yielding no reads (n = 12) were redesigned and those yielding low read counts (n = 71) had additional probe spiked into the pool, and the sequencing/analysis was repeated. After four rounds of optimization, final pools were reconstructed using the optimized amounts of each probe with reduced input of probes that produced very high read counts (≥ 2,000x) or represented a disproportionate fraction (≥ 5%) of the total reads from the sequenced sample. When all four pools of probes were reasonably balanced, we optimized combinations of pools for analysis. Multiple iterations resulted in a final protocol in which probes from pools 1, 2 and 3 (the lower G-C content pools) produced reasonably balanced sequence depth as an ensemble pool (SP1) with an input probe ratio from each pool of 1:1:2, respectively, which was used for ensemble probe capture and amplification. Probes from pool 4, with the highest G-C quartile (SP2) were separately used for capture and amplification. After amplification we found that making a final sequencing pool composed of 70% SP1 (pools 1, 2 and 3) and 30% SP2 (pool 4). Collectively, this optimization increased the coverage across all targeted sequence from an initial result of 33% of probes with at least 8 independent reads to 97% in the final protocol (**Figure S5**). Analysis showed high sensitivity and precision for detecting known rare variants (**Figure S6; Table S2**). In full production, 96.5% of all bases had at least 8 independent reads, with a median read coverage of 122 (**Figure S7**).

To improve genomic alignment, forward and reverse sequencing reads from each paired read were merged using PEAR v0.9.11 (9). Merged reads are then aligned to both genome build hg38 for quality control and b37 for variant calling using BWA-MEM v7.17 (10). Next, quality control is performed by reporting the number of paired-reads matched to probes, the number of paired-reads discarded for low quality UMIs, the number of the number of PCR duplicates removed, the number of forward and reverse reads that were merged, and the number of reads aligned to the genome, and within MIPseq target regions

using in-house scripts and BEDTools v2.27.1 (11). The number of unique target captures per probe and coverage per targeted base using Sambamba v0.7.1 (12) in conjunction with a custom script. Next, variants were called using GATK v3.7 (13) using default parameters, except for disabling of variant quality score recalibration due to lack of training SNPs in the panel, and Freebayes v1.3.2 (14) using default parameters. The union of these variant calls were annotated using ANNOVAR (15), multiallelic sites were split with BCFtools (16), and insertion-deletion variants were left-aligned with BCFtools.

In production, samples with an extremely high PCR duplicate rate (≥ 95%), indicative of very low DNA quality, were removed. Samples that had fewer than 90% of targeted bases covered by 8 or more independent reads with high-quality bases (Phred-scaled base quality score ≥ 20) were re-sequenced. Specifically, we identified which sequencing pool (SP1, SP2, or both) contributed to the low target sequence. Library preparation was repeated and re-sequencing was performed on the selected pools. The sample was then processed through the MIPseq computational pipeline using both original and supplemental reads from re-sequencing. Base coverage was subsequently reassessed and samples with fewer than 85% of targeted bases with 8 or more independent reads were removed.

Calculation of Sensitivity and Precision in MIPseq versus benchmarking samples

To optimize both target capture and computational processing pipelines, we employed two datasets for benchmarking: (A) Genome in a Bottle (GIAB) sample NA12878 with gold-standard variants validated by multiple sequencing platforms (17); (B) 170 PCGC CHD samples that were previously subjected to WES. Variants from NA12878 (High Confidence v3.3) were provided by the GIAB consortium. Variants from WES of PCGC samples were called using an in-house script, as described previously (2), that corresponds to the MIPseq variant filtering criteria. Computational down-sampling of paired-reads during testing of validation samples was performed using an in-house script that randomly selected paired-reads without bias for position in source FASTQ file which contained raw paired-end sequencing reads.

To quantitatively assess the variant calling performance of the MIPseq pipeline, we determined the sensitivity (recall) and precision (positive predictive value) by comparing MIPseq variant calls with true positive variants in NA12878. Sensitivity and specificity were calculated as follows:

Sensitivity = (MIPseq TPs) / (MIPseq TPs + MIPseq FNs)

Precision = (MIPseq TPs) / (MIPseq TPs + MIPseq FPs)

Here, “MIPseq TPs” are variants called by the MIPseq pipeline that intersect with true positives from GIAB, “MIPseq FPs” are variants called by the MIPseq pipeline that do not intersect with true positives from GiaB, and “MIPseq FNs” are variants called by GIAB, but not the MIPseq pipeline. Additionally,

we limited this analysis to variants that fall within the intersection of MIPseq target regions and the "high confidence" regions as recommended by GIAB; this encompasses a total region of 720kb (66% of the coding + 2bp canonical splice region of the panel).

To more quantitatively compare the concordance of called variants between MIPseq and WES, we extended the definition of sensitivity and precision to be calculated as follows:

$Sensitivity_{MIPseq}$ = (# MIPseq variants) / (# MIPseq ∪ WES variants)

$Sensitivity_{WES}$ = (# WES variants) / (# MIPseq ∪ WES variants)

$Precision_{MIPseq}$ = (# MIPseq ∩ WES variants) / (# MIPseq variants)

$Precision_{WES}$ = (# MIPseq ∩ WES variants) / (# WES variants)

Here, the intersection is calculated by removing any variant from the analysis that was either not well-covered in both datasets (fewer than 8 independent reads) or had evidence of the variant, but failed variant quality criteria (VAF, MQ, GQ) in either dataset. The results showed high sensitivity and precision of calling (**Figure S6**).

To orthogonally validate candidate variants called by the MIPseq pipeline in CHD experimental samples, we performed PCR amplification followed by Sanger sequencing on 59 variants of diverse types using custom primers at the Yale Keck DNA Sequencing Facility. All called variants tested were validated (**Table S2**).

Cohort Sequencing

MIPseq using the 248 CHD candidate gene panel was performed as described above on saliva or peripheral blood from 5,929 cases that had not yet been studied by prior WES and were absent of reported clinical diagnoses of large chromosome abnormalities or syndromic disease caused by genes not in the panel. We further filtered for cases that had sufficient available DNA for MIPseq (≥ 1mg), suitable DNA concentration to perform MIPseq (≥ 25 ng/ul), and had passed all quality checks from the biobank that stored these samples. Samples with fewer than 85% of panel-targeted bases with 8 or more independent reads consisting of high-quality bases (Phred-scaled genotype quality ≥ 20) were excluded from further study.

For the 5,626 cases, including 3,752 trios, that were previously reported were subjected to WES using described methods (2). Briefly: saliva or peripheral blood samples are collected from cases and, if available, parents. DNA was isolated and WES was performed using Roche SeqCap EZ MedExome Target Enrichment kit or IDT xGen target capture kit followed by 99 base paired-end sequencing on the

Illumina HiSeq 4000 or NovaSeq platforms. Cases with fewer than 85% of exome-targeted bases with 8 or more independent reads were excluded from further study.

The control cohort is composed of 133,743 samples with WES or WGS from the gnomADv2 database after exclusion of samples that are also present in the TopMed database (5). TOPMed samples were excluded because TOPMed's BRAVO variant database was used as a minor allele frequency (MAF) filtering criteria. Data regarding sample coverage, ethnicity, and identified variants was downloaded from the gnomAD repository (5). Parental and sample-level data for gnomAD samples was not available, therefore, variant phasing could not be determined.

Annotation of variants from MIPseq and WES datasets

Variants were annotated for most severe functional consequence among all potential isoforms using ANNOVAR and Meta-SVM from gnomAD v2.1.1 in both cases and controls. Loss of function variants (LOFs) are defined as variants that lead to frameshift insertion or deletion, stopgain, stoploss, or disruption of a canonical splice-site. Damaging missense variants ("D-Mis") are defined as missense variants annotated as "deleterious" by Meta-SVM. All remaining missense variants that are either annotated as "tolerant" by Meta-SVM or do not have Meta-SVM annotation are considered as tolerated missense variants ("T-Mis") in this analysis. Missense variants ("Mis") is a combination of all D-Mis and T-Mis variants. Protein-damaging variants ("Damaging") are defined as LOF or D-Mis variants. Protein-altering variants ("Altering") are defined as LOF, D-Mis, or T-Mis variants. Non-frameshift insertion or deletion variants were not included in the analysis, nor were non-exonic variants that were not predicted to disrupt a canonical splice-site by ANNOVAR (15).

Identification and analysis of DNMs in trios

In parent-proband trios, DNMs were identified using TrioDeNovo (18) and subsequently filtered as previously described (2). In detail: we filtered variants using the following criteria: we require variants to have ≥ 8 independent reads in the proband, ≥ 5 variant-supporting independent reads in the proband, minimum variant allele fraction (VAF) $\geq 20\%$ for variants with proband depth ≥ 10 and $\geq 28\%$ otherwise. Additionally, variants should have mapping quality (MQ) ≥ 40 and genotype quality (GQ) ≥ 20. Further, variants with parental depth < 10 or parental VAF $\geq 3.5\%$ were removed. All candidate variants were visually inspected *in silico* in the proband and both parents.

To calculate enrichment of DNMs in CHD patients, we performed a gene-based Poisson test of the observed variants versus a mutability-based expectation as previously described (2). This was performed

for each variant class, including synonymous, T-Mis, D-Mis, all missense, LOF, Damaging, and Altering. Per gene aggregate mutability was calculated as previously described (2). The lambda genomic inflation per thousand (λ_{1000}) was calculated as previously described (19). To determine the enrichment of DNMs in sets of multiple genes, the observed and expected counts were summated across all genes included in a given set.

We estimated the number of expected genes with multiple DNMs as described previously.(2) Briefly, we take the number of DNMs found and redistribute these based on mutability exome-wide or within the MIPseq panel genes. Enrichment is calculated by comparing the observed to the mean of all simulations.

Identification and analysis of very rare TUVs in patients and controls

To assess the association of rare variants, variant-aggregating gene-burden case-control tests have been classically utilized (20, 21). Although rare-variant association methods that utilize linear regression methods to adjust for covariates, such as SKAT-O (22) and VAAST (23), have been developed, these require exome-wide data and variant-level data for controls and therefore cannot be utilized in this study design (24). Instead, we generated and optimized filtering criteria to yield a gene-based burden case-control test that is robustly well-controlled.

A principal concern when performing a case-control test is to ensure cases and controls are well-matched to avoid spurious associations. To that end, we optimized filtering criteria. Specifically, we filtered for variants with MAF ≤ 10^{-5} in both BRAVO (25) and EVS (26) databases, MAF ≤ 1.3×10^{-4} within the cohort, ≥ 10 independent reads, ≥ 3 variant-supporting independent reads, VAF ≥ 20%, MQ ≥ 40, and GQ ≥ 20. BRAVO variants were lifted over from hg38 to hg19 using LiftOver (27). In cases, all variants were visualized *in silico*. In gnomAD controls, the 100 most frequent very rare variants were visualized *in silico* using at least three representative variants from the online gnomAD browser (5) and those found to be false positives were removed. The in-cohort filter was incorporated to remove likely false positive variants that were the result of systematic differences between pipelines and platforms used in cases, controls, and the BRAVO and EVS variant databases. We calculated that this in-cohort MAF permits 3 recurrent variants in cases and 35 in gnomAD controls.

These variant filtering criteria were rigorously optimized to match or exceed the stringent filtering criteria applied to the gnomAD variants prior to download availability. We further harmonized cases with controls by limiting variants to regions that were well captured by all platforms (*i.e.* WES in cases, MIPseq in cases, WES in gnomAD, and WGS in gnomAD); this removed ~2,000 coding or exon-flanking

bases in total from MIPseq and WGS samples, primarily from alternative transcripts not captured by some WES capture kits. The lambda genomic inflation per thousand (λ_{1000}) was calculated as described above.

To calculate enrichment of TUVs in cases, we tabulated a 2x2 contingency table by counting the number of cases or controls that have a mutant allele in the tested burden region (*i.e.* gene or set of genes) and those that did not. We calculated p-values using a one-tailed Fisher's Exact Test (FET) using the fraction of mutant alleles in CHD cases versus the fraction of mutant alleles in gnomAD controls. Given that sample-level data was not available for gnomAD controls, we used number of mutant alleles, rather than number of samples with or without a mutant allele for this analysis. Although it is possible CHD cases may have a higher rate of samples with multiple deleterious variants per gene versus gnomAD controls, we identified only none of our CHD cases had multiple ultra-rare LOF TUVs per gene and 46/4,598 (1%) with multiple ultra-rare Damaging TUVs per gene. This suggests testing at an allele-level, rather than sample level, has not biased the analysis. Additionally, given that in our panel design we sought to only identify pathogenic, rather than protective, variants, we used a used a FET. To determine total allele number (AN) for cases and controls in the FET, we corrected for platform differences by using a base-pair resolution coverage normalization method to independently adjust for total allele number per gene in cases and controls. Specifically, this was calculated as follows:

$$AN_A = \text{mean}(\%\ \text{bases} \geq 10x)_{\text{samples}}\ x\ (n_{\text{males}}\ x\ 2 + n_{\text{females}}\ x\ 2)$$

$$AN_X = \text{mean}(\%\ \text{bases} \geq 10x)_{\text{samples}}\ x\ (n_{\text{males}} + n_{\text{females}}\ x\ 2)$$

$$AN_Y = \text{mean}(\%\ \text{bases} \geq 10x)_{\text{samples}}\ x\ (n_{\text{males}})$$

Where AN_A denotes the adjusted allele number for autosomal genes, AN_X for x-linked genes, and AN_Y for Y-linked genes. "Samples" is the total number of male and female samples. Additionally, sample number includes only samples from cases or controls on platforms that capture a given gene (*e.g.* a sample captured on MIPseqv1 will not be included in the count of a MIPseqv3-specific gene). AN was independently calculated on each gene and for each variant class.

To determine the enrichment of TUVs when testing more than one gene, the observed allele counts for cases or controls were summated and total allele number was determined using the maximum allele number across any included gene in the set. The set of 'negative control genes' were selected based on damaging constraint metrics (tolerant to LOFs with pLI < 0.9 and permissive of missense variants with Mis Z-Score < 2) and a lack of *a priori* evidence for a pathogenic role in CHD.

Transmission Disequilibrium Test in rare variants from WES Trios

For the 60 significant genes in the MIPseq panel, transmission disequilibrium testing (TDT) was performed, as described previously (28), for rare damaging variants in the 3,887 WES trios. The significance of transmission disequilibrium was calculated by chi-square tests and the genotypic risk ratio was calculated as previously described (29).

Meta-analysis of DNMs and very rare TUVs using JL-FDR method

In parallel for each variant class, we performed a gene-level meta-analysis of the DNM Poisson p-values and the Fisher's test p-values from TUV case-control using the Joint-Local False Discovery Rate ("JL-FDR") method (30). Briefly, the JL-FDR method plots test statistic pairs from independent tests onto a coordinate plane and optimizes a rejection region using a maximum likelihood expectation algorithm that controls the FDR to a specified value; for each pair, the method produces a p-value and FDR value [**Figure 3.2**]. In the JL-FDR method, the FDR produced accounts for multiple-testing regardless of the number of datasets included (*e.g.* p-values from burden testing each of the six cardiac lesion groups). The lambda genomic inflation per thousand ($\boldsymbol{\lambda}_{1000}$) was calculated as described above.

Unless otherwise specified, we used two thresholds for statistical significance. Our baseline threshold was a false discovery rate (FDR) < 0.05 based on either Benjamini-Hochberg adjustment or via the J-L FDR method. Although at most only 248 genes were tested during analysis of the MIPseq panel, we also applied a more stringent cutoff that accounts for burden testing of all ~19,000 protein-coding genes typically present in WES; using a Bonferroni correction for 19,000 genes on a p-value cutoff of 0.05, we calculated this genome-wide significant (GWS) cutoff to be 2.62×10^{-6}, as we had applied previously (2).

Estimating the total number of risk genes in MIPseq panel

To estimate the total number of CHD risk genes in the 248 gene MIPseq panel using the JL-FDR framework (30), we calculate the proportion of genes that are expected to be associated with CHD based on the contribution of Damaging DNMs and TUVs. Here π_0 is the proportion of genes having no association with CHD, which can be estimated by fitting the following two-component bivariate normal mixture distribution with an EM-algorithm (31):

$$\binom{z_1}{z_2} \sim \pi_0 N(0, I) + (1 - \pi_0) N(0, I + \Sigma),$$

In the model, z_1 and z_2 are z-scores of the mutability-based gene burden test for DNMs and the case/control test for transmitted/unphased rare variants, which can be calculated by transforming corresponding p-values with the inverse cumulative density function of N(0,1), i.e., $z = -\Phi^{-1}(p/2)$. Under the null hypothesis in which the gene has no association with CHD, we have $z \sim N(0,1)$. In

addition, I is an identity matrix, and Σ is a 2×2 covariance matrix indicating the general dependency of effect sizes among the two tests when the gene indeed has an association with CHD.

Calculation of proportion of cases attributable to DNMs and ultra-rare TUVs

To calculate the contribution of DNMs to disease in CHD cases, we applied the method previously established (2):

% WES Trios Explained = (Observed DNM rate in cases – Expected DNM rate from mutability)

Extending the same methodology to ultra-rare TUVs by substituting *de novo* expectation with mutant allele rate in gnomAD controls, we can similarly calculate the proportion of cases attributable to ultra-rare Damaging TUVs:

% Cases Explained = (Observed Damaging rate in cases – Observed Damaging rate in controls)

Molecular Inversion Probe (MIP)-Based Targeted Sequencing Protocol

Based on protocol designed by *I.R. Tikhonova* at the Yale Center for Genomic Analysis

I. Probe Pooling and Phosphorylation

Reagents

- T4 polynucleotide kinase (PNK) – NEB, Part #M0201L
- 10X T4 DNA ligase buffer – NEB, Part #B0202S

Protocol

1. Pool 2 ul of each probe into a 1.5 ml microcentrifuge tube.
2. Place 85 ul of the pooled probe mix (Pool-1) in a 0.2 ml PCR tube. Add 10 ul of 10X T4 DNA ligase buffer (with 10 nM dATP) and 5 ul of (50 units) of T4 Polynucleotide Kinase. The total volume should be 100 ul.
3. Pipette up-and-down 10 times.
4. Incubate at 37°C for 45 minutes to phosphorylate the probes and then 20 minutes at 80°C to inactivate the kinase.
5. Phosphorylated probes can be stored at -20°C.

II. Probe Hybridization

Reagents

- Phosphorylated Targeting Probes
- 10X Ampligase DNA ligase buffer – Epicentre, Part #A1905B
- xGen Hybridization Buffer Enhancer – IDT, Part #1072278

Protocol

1. Serially dilute stock probes eight times from 1:50 – 1:800 with water in a 0.2 ml PCR strip. Use a 1:800 dilution for initial test and further proceed with the best reaction (clear band, efficient reaction, etc.); we generally end up using 1:800.
2. For probes with low target GC content (< 60%), add 2 ul of 10X Ampligase buffer, 2 ul of diluted probes, and an appropriate amount of accurately quantified genomic DNA (we use 300ng by PicoGreen for samples from Coriell) in each well of a 0.2 ml PCR strip. Bring the total volume to 20 ul with water.

   OR

   For probes with high target GC content (≥ 60%), add 2 ul of 10X Ampligase buffer, 2 ul of diluted probes, 3.2 ul IDT xGen Hybridization Enhancer, and an appropriate amount of accurately quantified genomic DNA (we use 300ng by PicoGreen for samples from Coriell) in each well of a 0.2 ml PCR strip. Bring the total volume to 20 ul with water.
3. Pipette up-and-down 10 times and briefly centrifuge.
4. Incubate in PCR block with heated lid at 98°C for 3 minutes, 85°C for 30 minutes, 60°C for 1 hour, 56°C for 2 hours, and hold at 25°C.

III. Gap-Fill and Ligation

Reagents

- 10X Ampligase DNA ligase buffer – Epicentre, Part# A1905B
- Ampligase DNA ligase – Epicentre, Part #A3210K
- 10X PCR_x Enhancer – Invitrogen, Part #11495-017
- 25 mM dNTP mix – NEB, Part #N0447L
- NAD+ - NEB, Part #B9007S
- Hemo KlenTaq DNA polymerase – NEB, Part #M0332L

Protocol

1. Add the master mix below to each well and pipette up-and-down 10 times to mix.

Reagents	ul
10X PCR_x Enhancer	2
Ampligase DNA ligase	2
10X Ampligase DNA ligase buffer	2
25 mM dNTP mix	2
NAD+	0.4
Hemo KlenTaq DNA polymerase	2
Water	9.6
Total	20

2. Incubate in PCR block with heated lid at 56°C for 60 minutes, 72°C for 20 minutes, and hold at 37°C.

IV. gDNA Digestion

Reagents

- Exonuclease I – NEB, Part# M0293L
- Exonuclease III – NEB, Part# M0206L

Protocol

1. Add 2 ul of Exonuclease I and 2 ul of Exonuclease III to each well and pipette up-and-down 10 times to mix.
2. Incubate in PCR block with heated lid at 37°C for 45 minutes, 80°C for 20 minutes, and hold at 4°C.

V. PCR Amplification

Reagents

- MIP Forward Barcoded Primer (25 uM)
- MIP Reverse Barcoded Primer (25 uM)
- 2X HiFi HotStart PCR Master Mix – KAPA, Part #KM2612

Protocol

1. Make a master mix of 25 ul of HiFi Master Mix, 2 ul of the forward primer, 2 ul of the reverse primer and 1 ul of water for each sample. Add 30 ul of PCR amplification master mix to 20 ul of sample and pipette up-and-down 10 times to mix.

2. Incubate in PCR block with heated lid with the following protocol:

   98°C for 45 seconds

3. 22 cycles of:

   98°C for 15 seconds

   55°C for 30 seconds

   72°C for 30 seconds

   72°C for 1 minute

   Hold at 4°C

4. Clean-up PCR product using 1.0X SPRI beads and elute in 24 ul of Nuclease free water.

VI. Quantitation by PicoGreen assay.

Reagents

- Quant-iT dsDNA Assay Kit, Broad Range – Invitrogen, Part# Q33130

Protocol

1. Remove the Quant-iT dsDNA Broad-Range Assay kit from refrigerator and allow the components to equilibrate to room temperature. During all steps, protect the Quant-iT dsDNA Broad-Range Assay reagent concentrate and working solution from light as much as possible.

2. Make a working solution by diluting Quant-iT dsDNA BR reagent 1:200 in Quant-iT dsDNA BR buffer. For example, for 40 assays put 100 μL of Quant-iT dsDNA BR reagent (Component A) and 20 mL of Quant-iT dsDNA BR buffer (Component B) in a 50-mL tube, mix well and cover tube with foil to protect from light.

3. Load 200 μL of the working solution into each well of a microplate (Greiner 96-well Black Microplate).
4. Add 10 μL of each DNA standard (Component C) to separate wells. Each standard loaded into two wells.
5. Add purified PCR product sample into two wells, 2 uL each. Seal plate and vortex 2 min. Briefly spin plate, remove the seal and immediately transfer to microplate reader (Molecular Devices SpectraMax M2).

6. Measure the fluorescence with excitation/emission maxima of 510/527 nm.
7. Determine standard curve by plotting standard’s fluorescence values.

8. Calculate DNA samples concentrations by using standards curve Equation.
9. Pool same amount of DNA from each well of purified PCR product into single Eppendorf tube.

VII. Agarose Gel DNA Extraction

Reagents

- E-Gel EX 2% Agarose – Invitrogen, Part #G402002
- 50 bp DNA Ladder – Invitrogen, Part #10416-014
- QIAquick Gel Extraction Kit – Qiagen, Part #28706

Protocol

1. Load one well with 50 bp ladder, three wells with pooled PCR product, and the remaining wells with water.
2. Run gel for 12 min using Invitrogen e-gel iBase system.
3. Cut gel slice at ~330 bp mark
4. Purify gel slice with QIAquick Gel Extraction Kit.
5. Elute with 35 ul EB.

Quality Control

1. Run 1 ul of sample on DNA 1000 Bioanalyzer chip.
2. Quantify sample by qPCR

Known or Candidate CHD Genes (n = 85)				Chromatin Modifier Genes (n = 163)						
Known CHD (n = 46)		≥ 2 dDNMs (n = 20)	Selected (n = 19)	Intolerant to LOFs (n = 132)					≥ 1 dDNM (n = 9)	Selected (n = 22)
ACTB	NODAL	AHNAK	ACE2	APBB1	CREBBP	KAT6A	NSD2	SMARCA1	BRMS1L	BRD9
ADNP	NOTCH1	CACNA1A	CSTF3	ARID1A	CTBP1	KAT6B	NSD3	SMARCA2	HLTF	CABIN1
ANKRD11	NOTCH2	CAD	CUL3	ARID1B	CTCF	KAT7	OGT	SMARCA4	KDM5B	CXXC1
ASXL1	PACS1	CLUH	DDX3X	ARID2	CTNNB1	KDM1A	PBRM1	SMARCA5	PRKAA2	DAPK3
BRAF	PRDM6	GANAB	EGFL7	ARID4A	CTR9	KDM2A	PHF2	SMARCB1	RNF20	DNMT3A
C1orf127	PTEN	ITSN2	EXTL3	ARID4B	CUL4B	KDM3A	PHF20	SMARCC1	TP53	EHMT2
CDK13	PTPN11	KLF2	HRNR	ASH1L	DDB1	KDM3B	PHF21A	SMARCC2	TRIM37	ELP3
DYRK1A	RAF1	KRT13	KLF4	ATRX	DNMT1	KDM4A	POGZ	SMARCD1	UBE2B	HDAC1
FBN1	RBFOX2	LHX2	PCBP3	ATXN7L3	DOT1L	KDM4B	PPP5C	SRCAP	USP16	HDAC3
FLT4	RIT1	MINK1	SNX1	BAG6	EHMT1	KDM5A	PRDM2	SUPT5H		KAT2A
FOXC2	RPL5	MYO7B	SSRP1	BAP1	EP300	KDM5C	PRMT1	SUPT6H		KAT5
GATA4	SHOC2	NAA15	SV2A	BAZ1B	EP400	KDM6A	PRMT5	TET3		LOXL2
GATA6	SMAD2	NGFR	SVEP1	BAZ2A	EPC1	KDM6B	PSME4	TLK2		MCRS1
GDF1	SMAD6	NR6A1	TBX18	BCOR	EYA3	KMT2A	RBBP4	TP63		MECP2
GJA1	SMC3	PPL	UBTF	BPTF	EZH2	KMT2C	RBM14	TRRAP		MED24
JAG1	SOS1	PPP1R12A	UMODL1	BRD1	HCFC1	KMT2D	RERE	UBN1		MTA2
KDR	SOS2	RABGAP1L	UNCX	BRD3	HDAC2	KMT2E	RNF40	UBR2		NCOA3
LRP1	TAB2	SCN10A	USP7	BRD4	HDAC5	KMT5B	RTF1	UHRF2		PER1
LZTR1	TBX1	SETD5	WDR26	BRPF3	HDAC7	MAP3K12	RUVBL2	USP15		SCMH1
MYH6	TBX5	U2SURP		CARM1	HDAC9	MBD3	SALL1	UTY		SETD3
MYH7	TSC1			CHD1	HIRA	MTA1	SAP130	WAC		SKP1
MYRF	ZEB2			CHD2	HUWE1	MTF2	SETD2	WDR5		SMARCD3
NKX2-5	ZIC3			CHD3	JARID2	MYC	SETDB1	YEATS2		
				CHD4	JMJD1C	NCOA1	SFPQ	ZMYND11		
				CHD6	JMJD6	NCOA2	SIN3A			
				CHD7	KANSL1	NCOR1	SIN3B			
				CHD8	KAT2B	NSD1	SMAD4			

Figure S1 | 248 known or putative CHD and chromatin modifier genes included in MIPseq gene panel

Genes subjected to MIPseq are listed and include 85 known or putative CHD genes and 163 chromatin modifier genes selected for based on primary selection criteria. Abbreviations: dDNMs, "Damaging *de novo* mutations"; LOF, "loss of function mutations"; and Intolerance to LOFs denotes pLI ≥ 0.9 in gnomAD database. Eighteen genes in the "chromatin modifier" group have also been identified as candidate CHD genes: *ARID1A*, *ASXL1*, *BCOR*, *CHD4*, *CHD7*, *CREBBP*, *EHMT1*, *EP300*, *KANSL1*, *KAT6B*, *KDM5B*, *KDM6A*, *NSD1*, *PRDM6*, *SALL1*, *SMAD4*, *SMARCA4*, and *SMARCB1*.

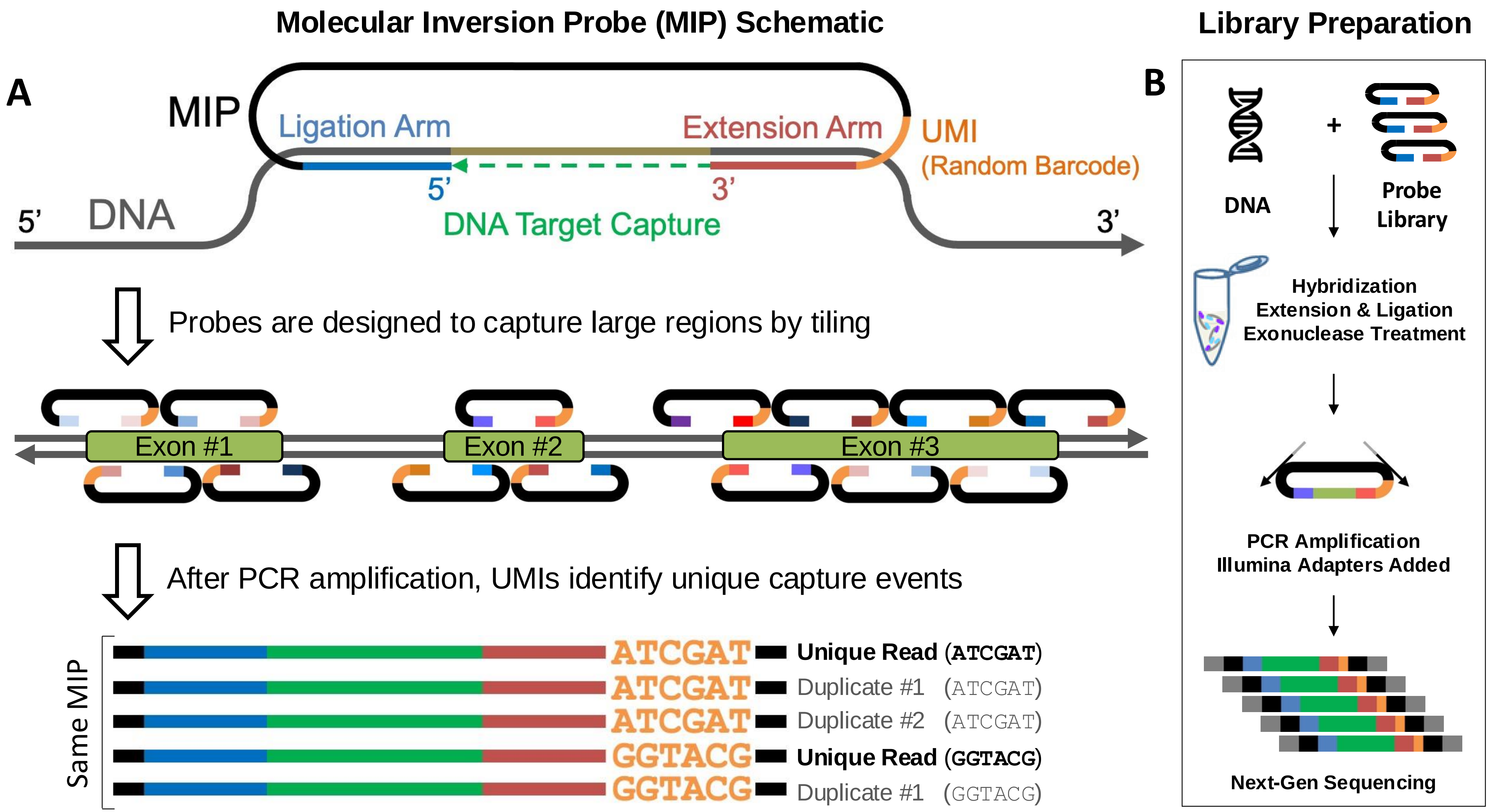

Figure S2 | Single-molecule Molecular Inversion Probe Sequencing (MIPseq) Method

[A] A schematic of a molecular inversion probe fill-in. The ligation primer arm is in blue, the extension primer arm is in red, the unique molecular barcode (UMI) is in orange, and backbone sequence is in black. The single-stranded probe is shown binding to complementary sequence on denatured, single-stranded target DNA. The DNA target is synthesized by gap-filling using DNA polymerase and ligated to produce a closed circle. *Middle*: The opposite-strand tiling design strategy to reliably capture overlapping genomic targets is depicted. *Bottom, example of* probes with captured target DNA following PCR amplification. Here, two probes with the same target produced six total PCR products. The two unique 6bp UMI sequences each correspond to one of the probes and thereby enable computational identification of PCR duplicates from each probe. **[B]** General workflow for MIPseq library preparation. A single-stranded MIP library pool is hybridized to sample DNA, probes are circularized through extension and ligation, isolated via exonuclease treatment, amplified through PCR using universal primers that add Illumina adapters (shown in dark gray), and then sequenced using 150bp paired-reads.

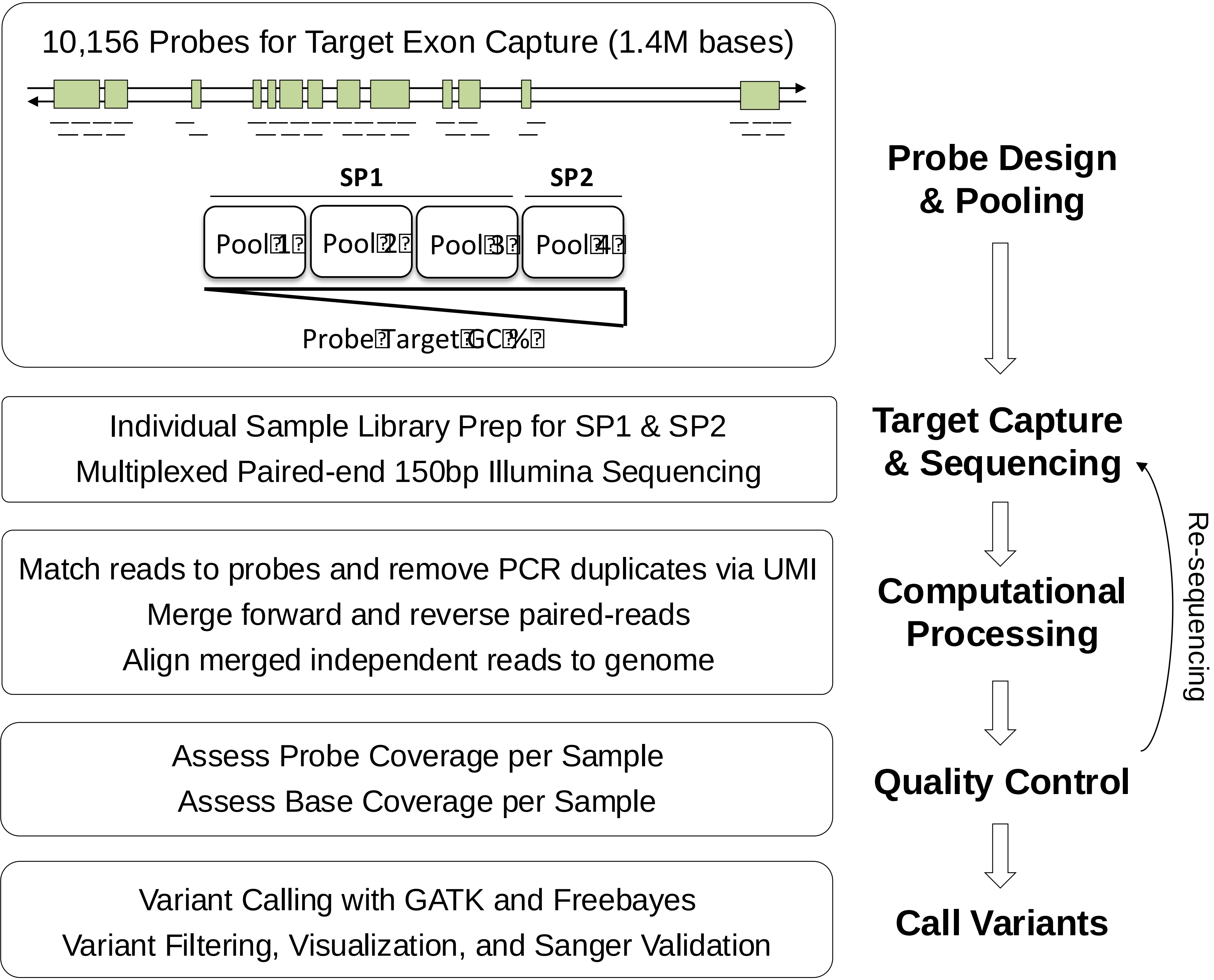

Figure S3 | Overview of MIPseq computational design, processing, and quality-control pipeline Probes were designed to capture overlapping exonic targets plus flanking canonical splice sites using MIPgen. Following synthesis, probes were pooled by quartile of % G-C content and phosphorylate at their 5'ends. Pools 1, 2 and 3 were combined at a ratio of 1:1:2 to constitute pool SP1, and gap filling and ligation was performed. Poll 4 was processed alone as SP2, Library preparation was performed independently on each sequencing pool (SP) and resulting capture products were multiplexed for high-throughput 150bp paired-end Illumina sequencing. Reads were processed, with those having duplicate Unique Molecular Identifiers (UMIs) removed. Variant calling was performed as described in Methods.

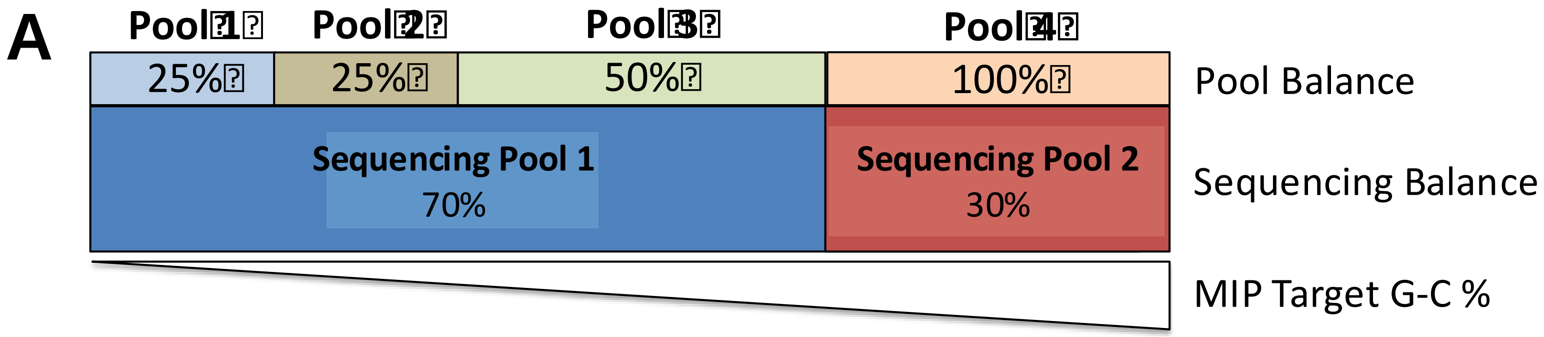

B

Sequencing Pool	Pool	Probes	% G-C Min	Max	Median	Mean
SP1	1	2,730	21	42	37	36
	2	2,579	42	51	46	47
	3	2,372	51	60	55	55
SP2	4	2,473	60	91	64	65

Figure S4 | Probe pooling strategy based on G-C content of probe target region

[A] Grouping of probes by probe target region G-C quartile into four 'Pools'. These four Pools are further arranged into two 'Sequencing Pools' (SP) in which SP1 incorporates Pools 1 to 3 and SP2 consists of Pool 4 alone. For each SP, the ratio of Pools is annotated as 'Pool Balance'. SPs are independently amplified and subsequently combined prior to high-throughout sequencing at the ratio annotated as 'Sequencing Balance'. A G-C gradient is provided to illustrate transition from lowest target G-C content in Pool 1 to greatest target G-C content in Pool 4. **[B]** Table shows the G-C target content percentage minimum (inclusive) and maximum (exclusive) that demarcate each of four Pools alongside median and mean values.

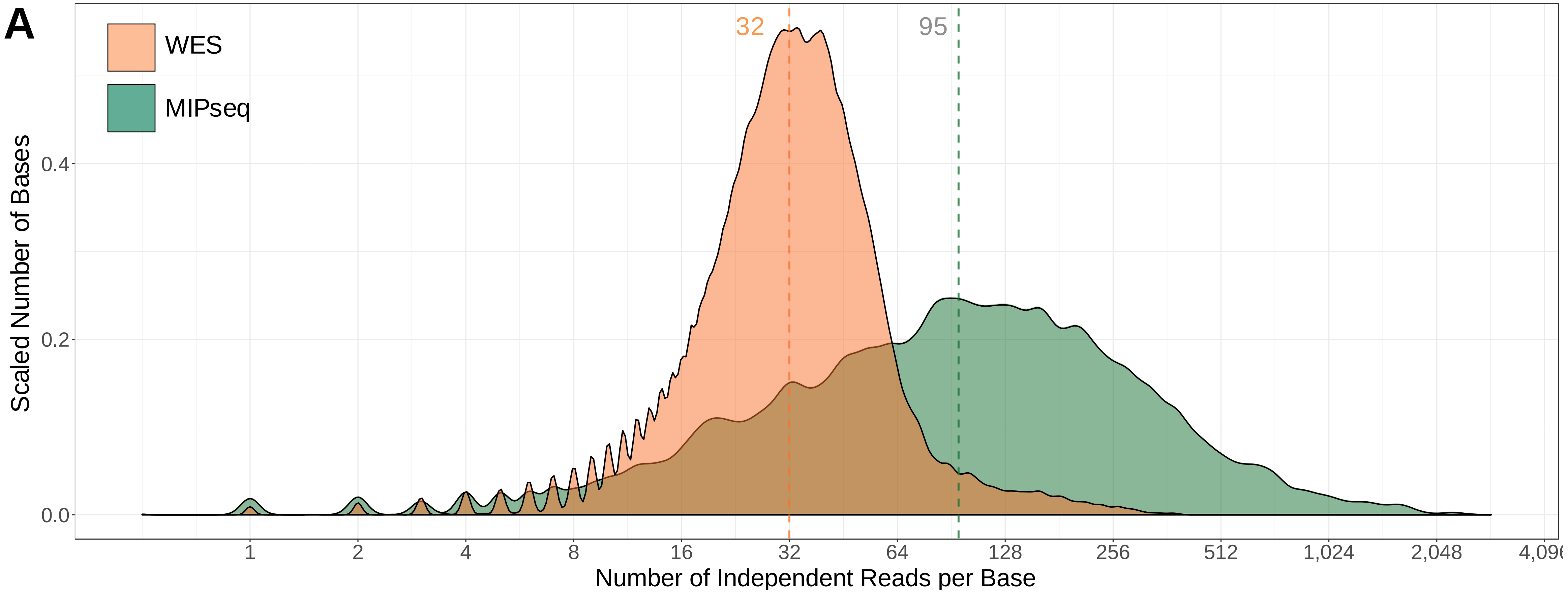

B

Platform	# Probands	Independent Reads				% Targeted Bases that Exceed				
		5th	Median	Mean	95th	0x	8x	10x	20x	50x
MIPseq	170	7	95	170	582	98.7%	94.9%	93.6%	83.3%	68.7%
WES	170	10	32	39	88	99.9%	97.0%	95.3%	67.9%	21.9%

Figure S5 | MIPseq pipeline demonstrates similar per-base coverage compared to WES on 170 matched PCGC probands
Distribution of number of independent reads per base in MIPseq and WES on matched cohort of 170 PCGC validation probands using mature MIPseq pipeline. **[A]** Density plot of base coverage distribution in 170 PCGC probands from MIPseq (green) versus WES (orange). The number of independent reads per base (coverage) is shown on a log2 scale on the x-axis and the number of bases with a given coverage is shown as a density on the y-axis. Dashed lines demarcate the median coverage in MIPseq (green) and WES (orange). Plot was generated using coverage data from all exons targeted by the MIPseq gene panel plus 18bp of flanking splice bases. **[B]** Corresponding table of base coverage statistics and percentage of targeted bases with more than the indicated number of independent reads across all samples from MIPseq or WES.

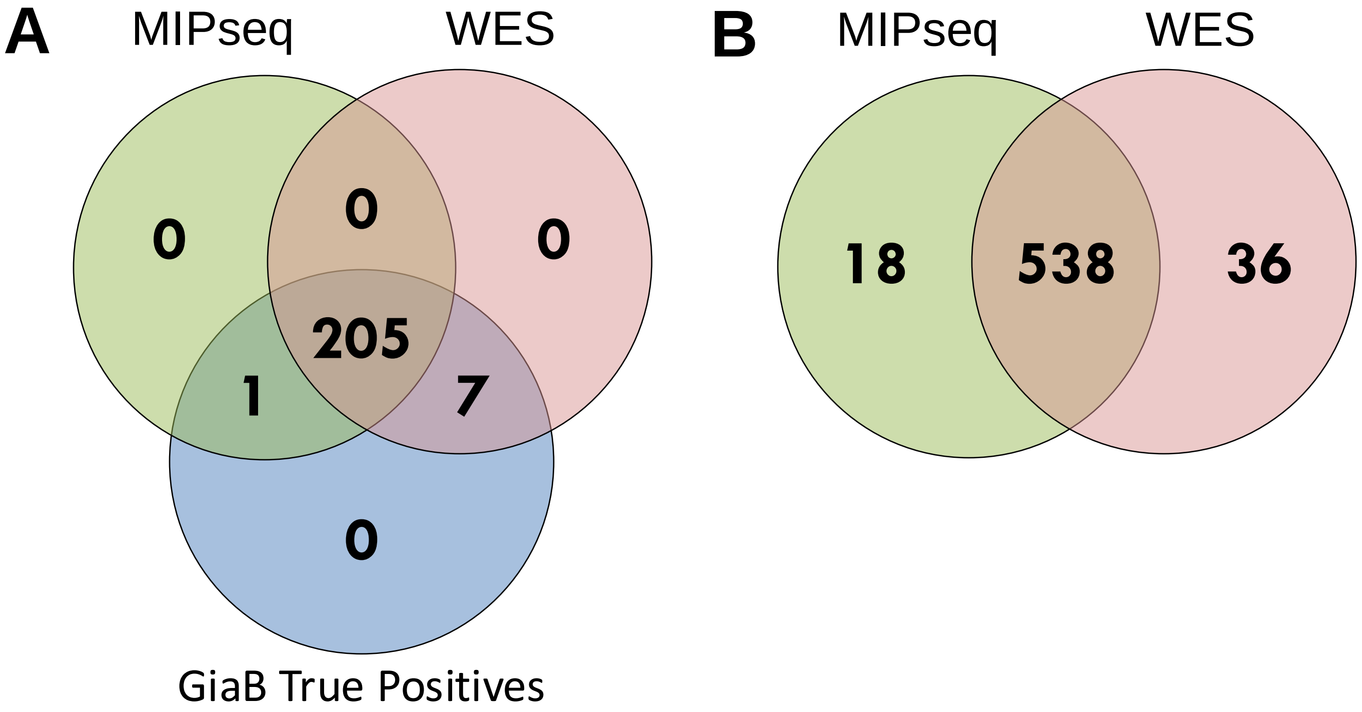

Platform	Sensitivity	Precision
MIPseq	96.2%	100%
WES	99.5%	100%

Platform	Sensitivity	Precision
MIPseq	93.6%	99.8%
WES	99.5%	100%

Figure S6 | High sensitivity and precision of MIPseq variant calls on training samples
Results of variant detection in training samples using WES and MIPseq. **[A]** Venn diagram of variant calling in GIAB sample NA12878. Consensus calls by GIAB consortium (blue), MIPseq (green), and WES (pink) are shown. Gold-standard calls from GIAB consortium totaled 213 variants at well-covered, high-quality positions, with no MAF filter applied. MIPseq had 7 false negatives due to low read depth depth or low variant allele fraction. In WES, there was 1 false negative due to low read depth. *Below, t*able shows sensitivity and precision for MIPseq and WES versus true positives. **[B]** Variant calling comparison in 170 PCGC CHD probands on which we had previously performed WES in-house. 592 rare (MAF ≤ 5e-5 in ExAC, EVS, and 1000 genomes databases) mutations were called at well-covered, high-quality mutations across all probands. *Upper*: A venn-diagram showing concordance between MIPseq and WES mutations. There were 36 ES-specific mutations not called in MIPseq due to low depth (n=26), low variant allele fraction (n=7), low mapping quality (n=2), or too few mutation-supporting reads (n=1). There were 18 MIPseq-specific variants not called in WES due to low depth (n=9) and VAF (n=8). Additionally, there was no evidence of 1 MIPseq-specific mutation in WES, despite the locus being well-covered in WES. *Lower*: Sensitivity and precision was calculated using a modified method since there are no true positives available for reference.

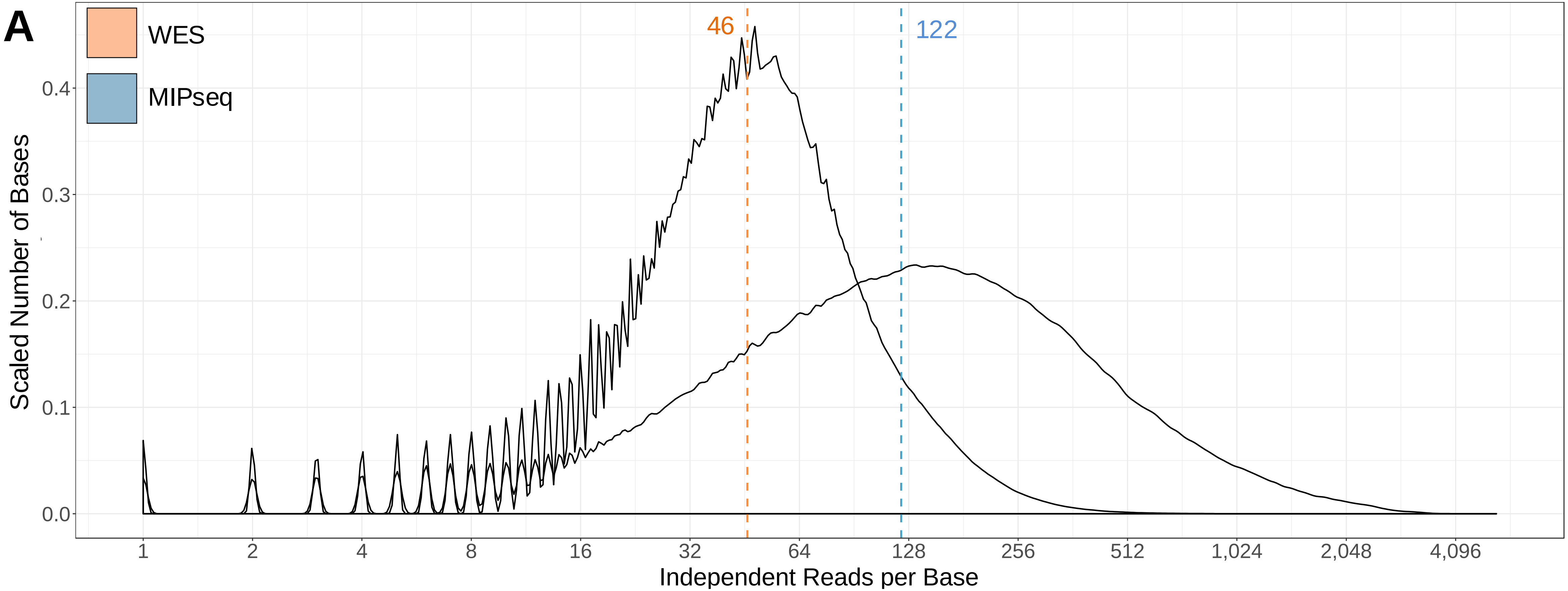

B

Platform	# Probands	Independent Reads				% Targeted Bases that Exceed					
		5th	Median	Mean	95th	0x	8x	10x	20x	50x	100x
MIPseq	5,929	8	122	222	779	99.0%	96.5%	95.7%	90.8%	75.7%	56.6%
WES	5,626	13	46	57	145	99.0%	96.3%	95.2%	86.7%	45.2%	12.3%

Figure S7 | MIPseq pipeline on experimental PCGC samples generates 10x base coverage comparable to WES probands
Distribution of number of independent reads per base in MIPseq and WES samples sequenced in this study. **[A]** Density plot of base coverage distribution in 5,929 MIPseq experimental PCGC probands (blue) versus an independent set of 5,626 WES experimental PCGC probands (orange). The number of independent reads per base (coverage) is shown on a log2 scale on the x-axis and the number of bases with a given coverage is shown as a density on the y-axis. Dashed lines demarcate the median coverage in MIPseq (blue) and WES (orange). Plot was generated using coverage data from all exons targeted by the MIPseq panel plus 2bp of flanking splice bases. **[B]** Corresponding table of base coverage statistics and percentage of targeted bases with more than the indicated number of independent reads across all samples from MIPseq or WES.

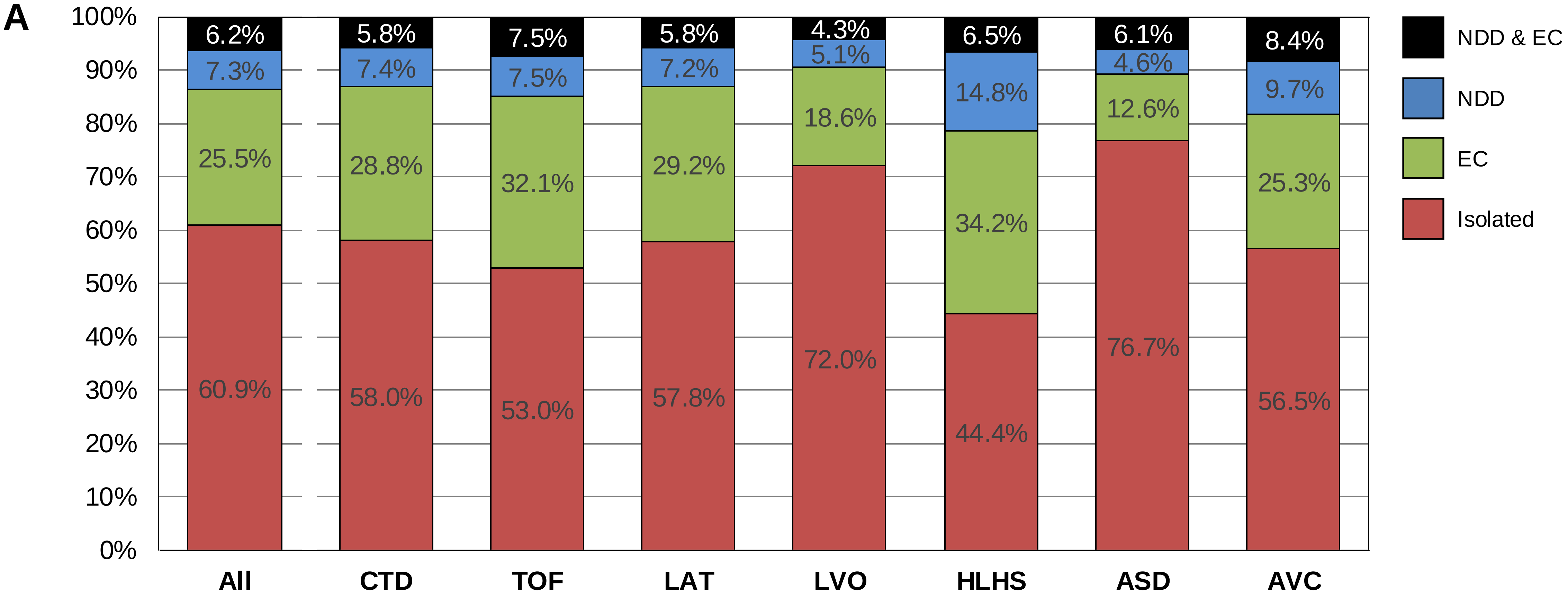

B

Pheno	Isolated	EC	NDD	NDD & EC	X^2 P-Val	X^2 P-Val
CTD	965	480	123	97		6.15E-02
TOF	597	361	84	84		2.74E-07
LAT	762	385	95	77		7.53E-02
LVO	853	220	60	51	< 2.2E-16	7.40E-18
HLHS	204	157	68	30		3.86E-15
ASD	468	77	28	37		2.80E-18
AVC	174	78	30	26		8.47E-02
**Total**	**4,023**	**1,758**	**488**	**402**	-	-

C

Pheno	Isolated	Not Isolated	X^2 P-Val	Fisher P-Val
CTD	965	700		2.56E-02
TOF	597	529		5.10E-08
LAT	762	557		3.85E-02
LVO	853	331	< 2.2E-16	1.19E-19
HLHS	204	255		8.80E-13
ASD	468	142		5.14E-18
AVC	174	134		1.80E-01
**Total**	**4,023**	**2,648**	-	-

Figure S8 | Significant variation in frequency of EC/NDD abnormalities in different CHD phenotypes

[A] Observed distribution of extracardiac phenotypes across seven pre-defined cardiac phenotypes; **[B]** Comparison of frequency of extracardiac phenotypes across each CHD subset, categories with p-values for a 7x4 chi-squared test and seven 2x2 chi-square tests for each cardiac phenotype; **[C]** simplified table of CHD phenotypes versus cases with either isolated CHD or cases with NDD and/or EC; as with B, table shows p-values for a 7x2 chi-square test and five 2x2 Fisher tests for each cardiac phenotype. No multiple testing correction was performed. Genes with $P < 0.05$ are colored in red.

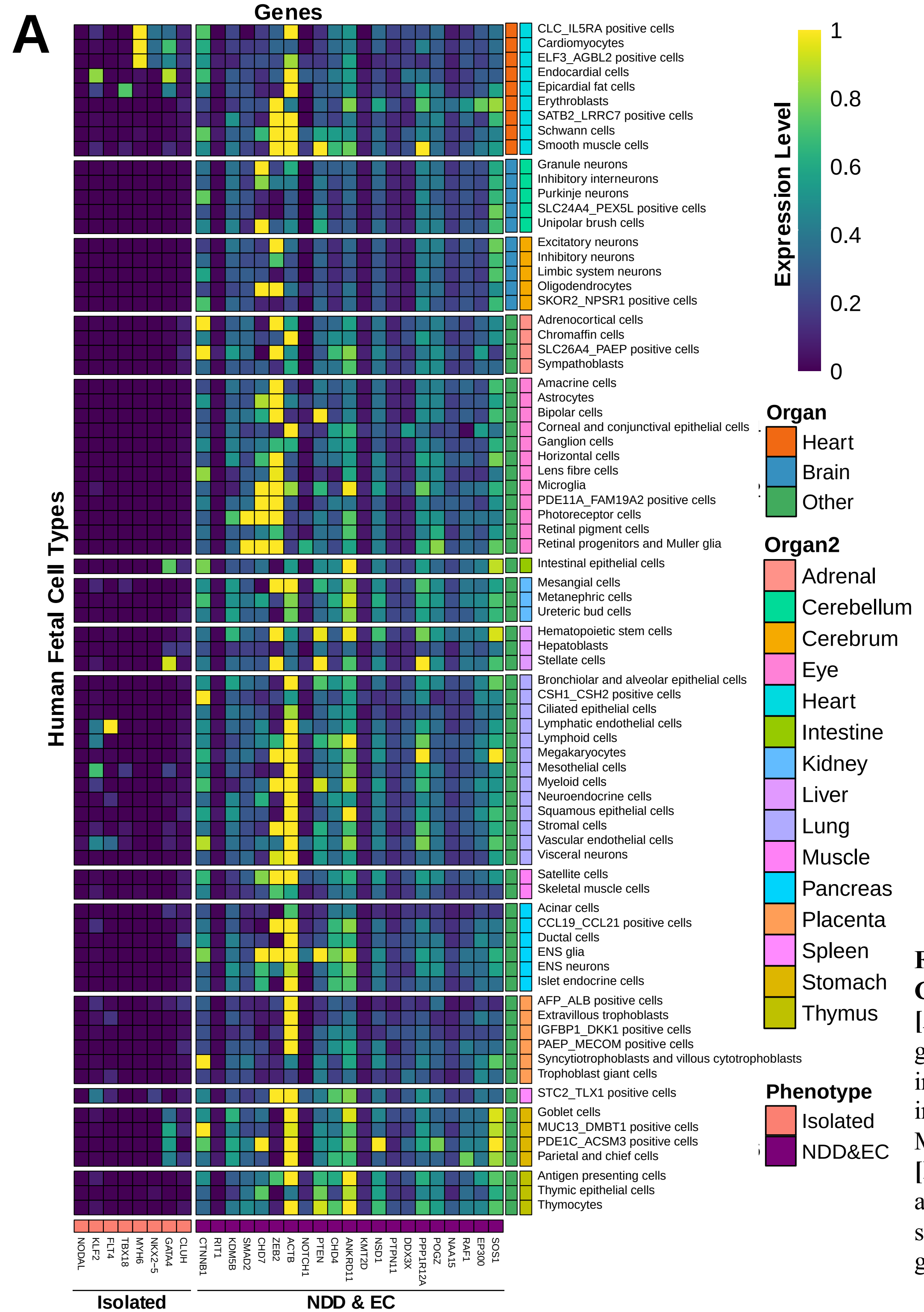

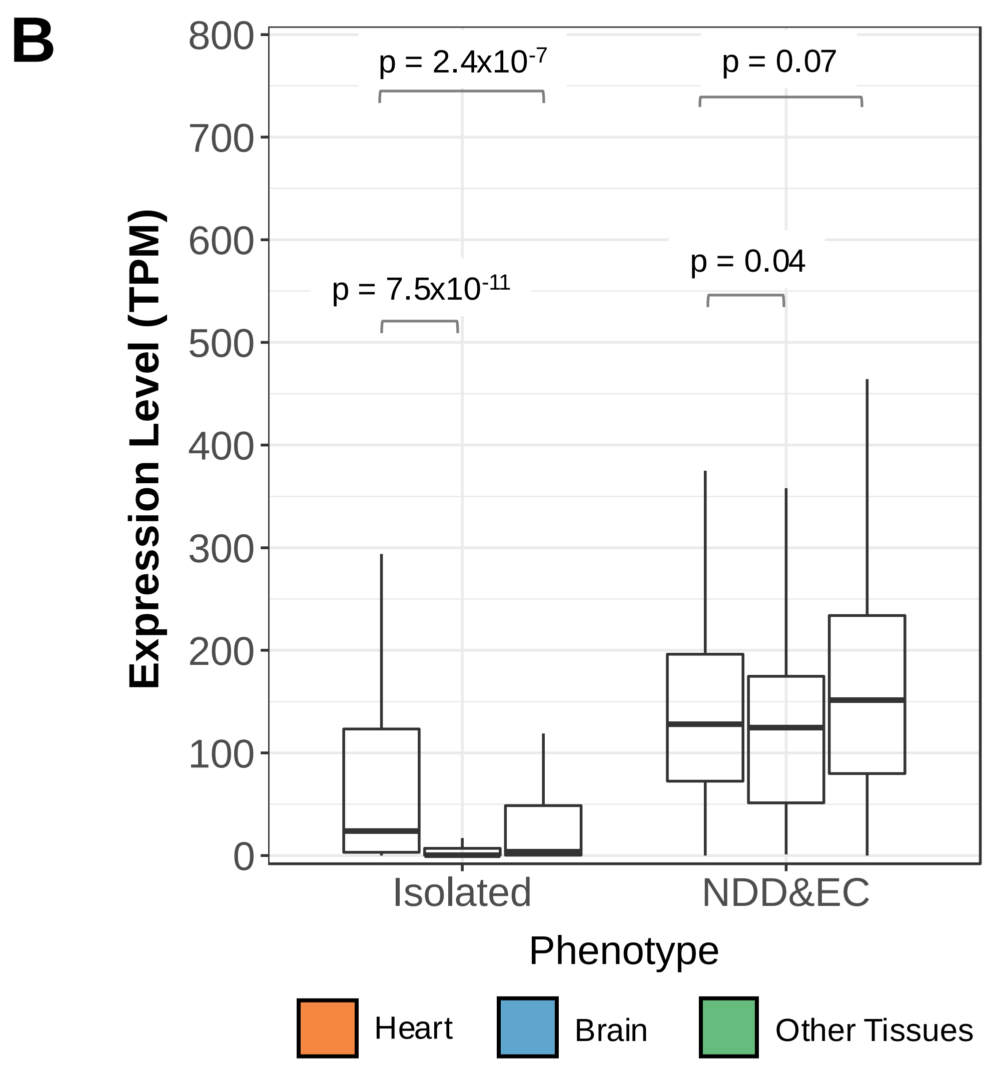

Figure S9 | Single-cell expression during human fetal development of genes enriched in probands with isolated CHD versus those with NDD and/or EC abnormalities

[A] Using a single-cell RNA-seq dataset during human fetal development from Cao *et. al.,* the expression levels of 29 genes significantly enriched in isolated CHD cases ("isolated"; in pink) or CHD cases with NDD&EC ("NDD&EC"; in purple) are shown. Cell types are grouped into heart (red), brain (blue), and all other tissues (green). 'Organ2' indicates a more specific tissue classification. Expression levels are shown scaled to the greatest TPM (Transcripts per Million Read) across all cell types reported in Cao *et al.*

[B] Quantitation of expression levels of genes associated with isolated and NDD&EC subsets. Expression levels from all cell types are grouped into heart, brain, and all other tissues categories. Medians values are shown. Wilcoxon rank-sum P-value comparing expression levels heart, brain and other tissues are shown. For isolated and NDD & EC groups.

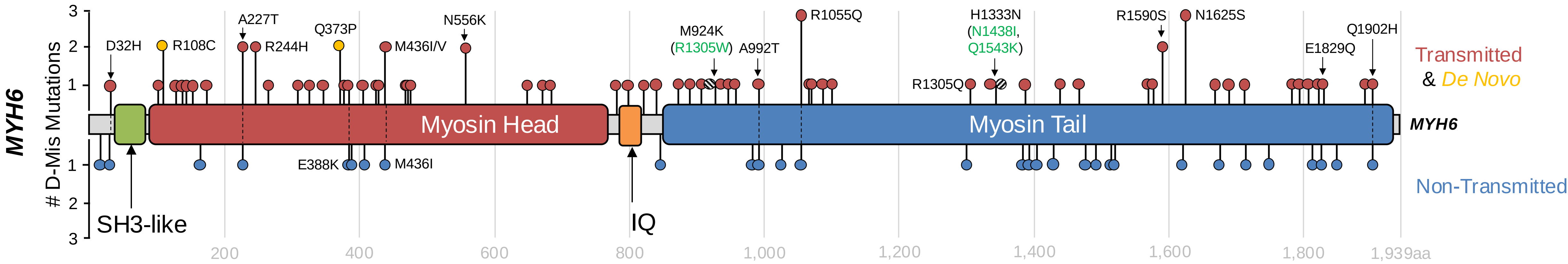

Figure S10 | Distribution of ultra-rare transmitted and non-transmitted D-mis variants in *MYH6*

Lollipop plots of ultra-rare transmitted and non-transmitted and *de novo* D-Mis mutations in *MYH6* from 3,887 CHD trios. A diagram of MYH6 is shown. Ultra-rare heterozygous missense mutations that were found in parents and transmitted to probands (n = 71) are shown in red above the diagram of the protein, while parental variants non-transmitted to probands (n = 30) are shown in blue below the diagram, and *de novo* mutations are shown in gold above the diagram (n=4). Two probands had >1 variant on the same allele; these are shown using a hashed pattern. One allele has variants M924K and R1305W; the other has H1333N, N148I, and Q1543K. Recurrent variants that were both transmitted and non-transmitted are connected by a dashed line. Recurrent variants are labeled with amino acid change. Amino acid position is shown along x-axis. Functional domains are annotated using PFAM database. Height of lollipop corresponds to number of each mutation observed.

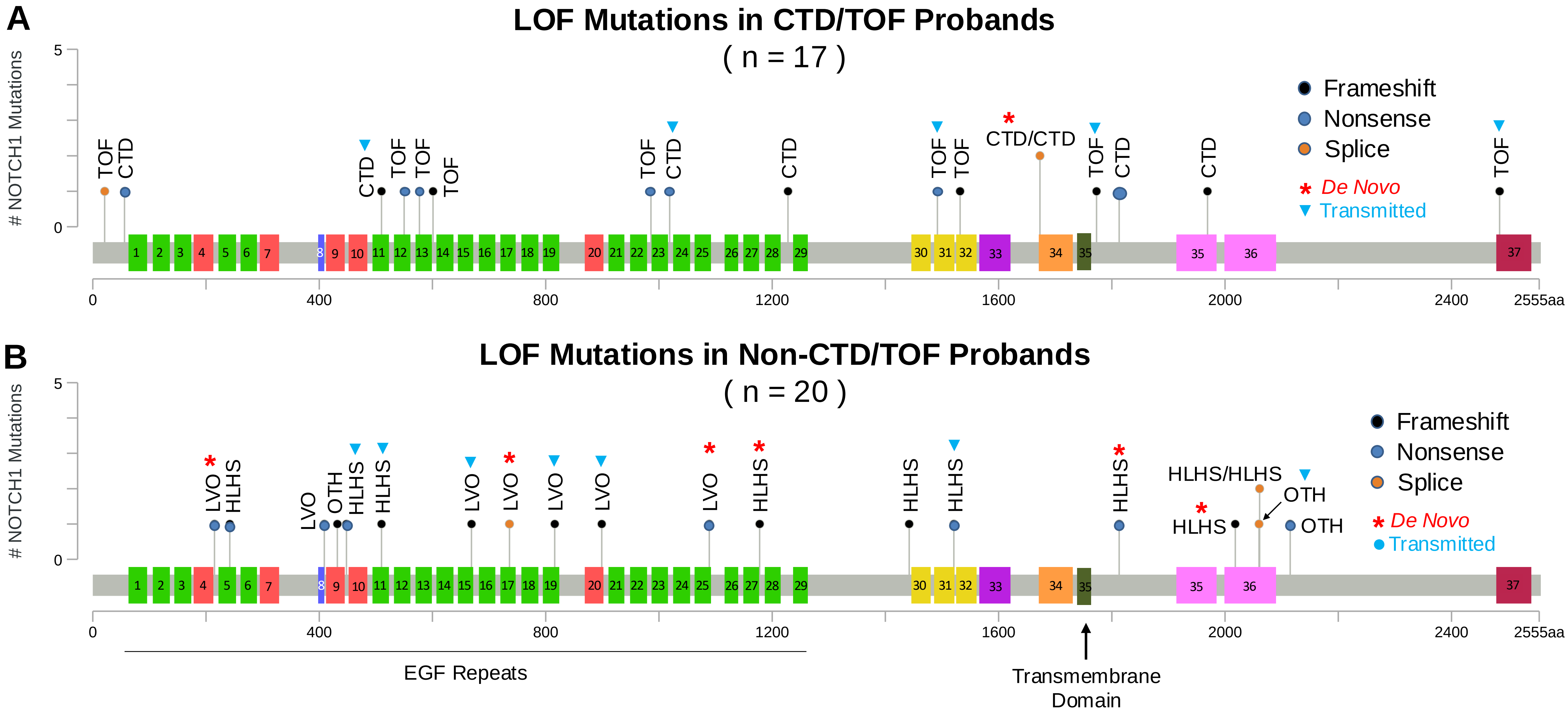

Figure S11 | Distribution of NOTCH1 LOF mutations in CTD/TOF and Non-CTD/TOF probands

Lollipop plot of *de novo*, transmitted, and unphased LOF mutations in *NOTCH1* from **[A]** CTD/TOF probands or **[B]** Non-CTD/TOF probands. Height of lollipops corresponds to number of mutations (labeled on y-axis). EGF-like domains are in green and numbered : “EGF-like calcium-binding” domains are in red, “human growth factor-like EGF” domains are in blue, “Notch LNR” domains in yellow, “Notch NOD” domains in purple, “Notch NODP” domains in orange, ”Ankyrin repeat-containing” domains in pink, and a domain of unknown function in maroon.

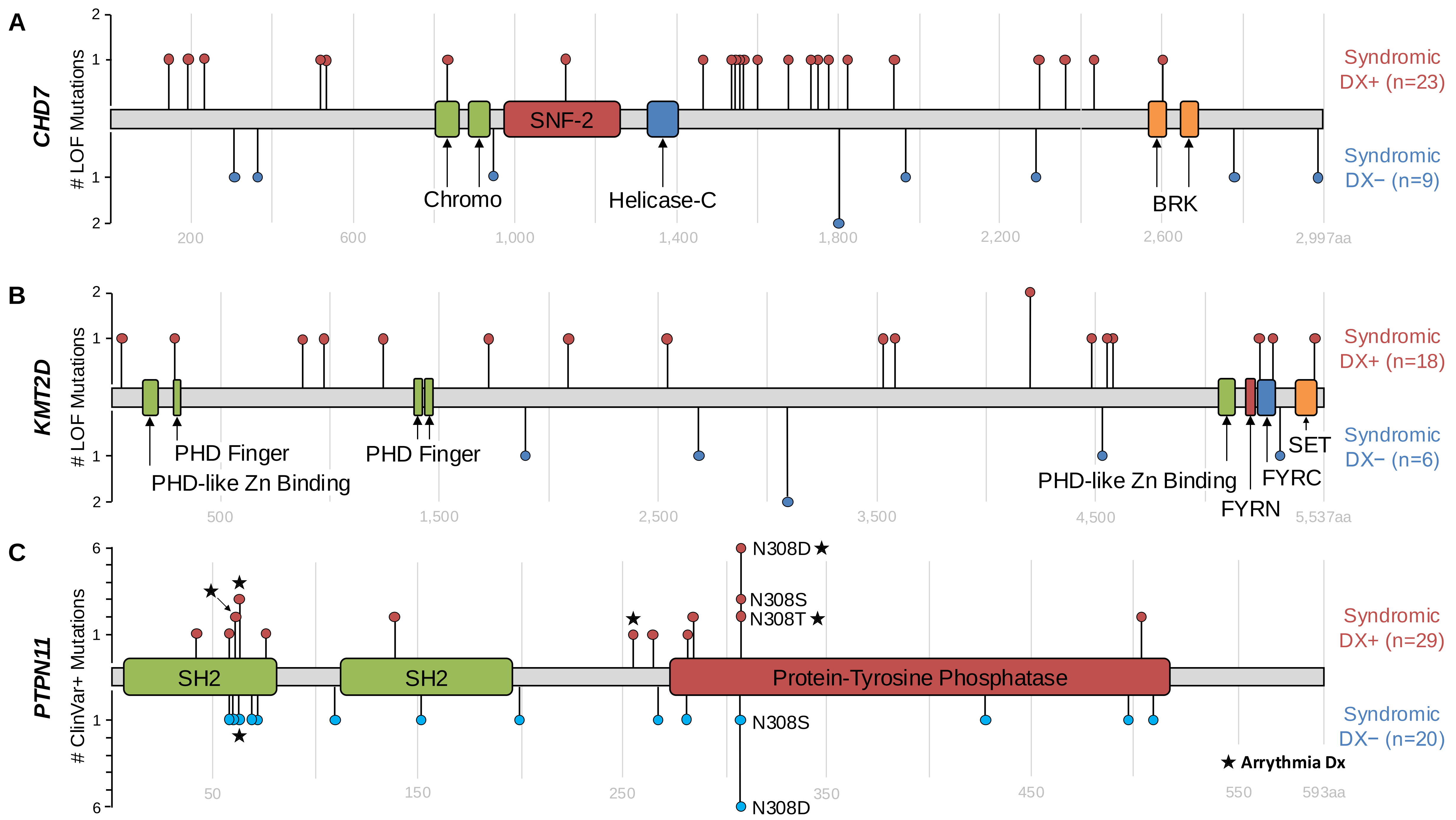

Figure S12 | Distribution of presumed pathogenic mutations and clinical diagnosis for selected genes. [A] Lollipop plot of 32 LOF mutations in *CHD7* from 11,555 CHD probands stratified by those with (Dx+) or without (Dx-) a clinical diagnosis of CHARGE syndrome. **[B]** Plot of 24 LOF mutations in *KMT2D* from 11,555 CHD probands with or without a clinical diagnosis of Kabuki syndrome. **[C]** Plot of 49 ClinVar Pathogenic missense mutations in *PTPN11* from 11,555 CHD with or without a clinical diagnosis of RASopathy or Noonan syndrome. Different mutations at the same amino acid are independently labeled. Amino acid position is shown along x-axis. Functional domains are annotated using PFAM database. Stars identify each proband with arrhythmia.

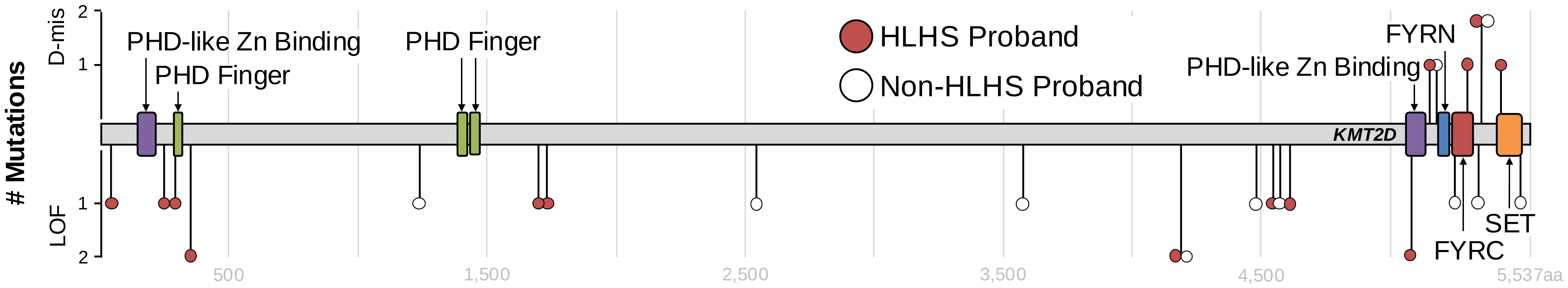

Figure S13 | *De novo KMT2D* D-Mis mutations all cluster in the C-terminus and are enriched for HLHS
The locations of *KMT2D de novo* mutations from 3,887 CHD probands are shown along the encoded protein, with D-mis mutations shown above and LOF mutations below the protein depiction. Red lollipops denote mutations in HLHS probands and white denotes non-HLHS probands. Unphased mutations are not shown. Functional domains are annotated using PFAM database.

Number of nodes	60
Number of edges	209
Avg. node degree	6.97
Avg. local clustering coefficient	0.52
Expected number of edges	77
PPI Enrichment P-Value	**< 1.0x10^{-16}**

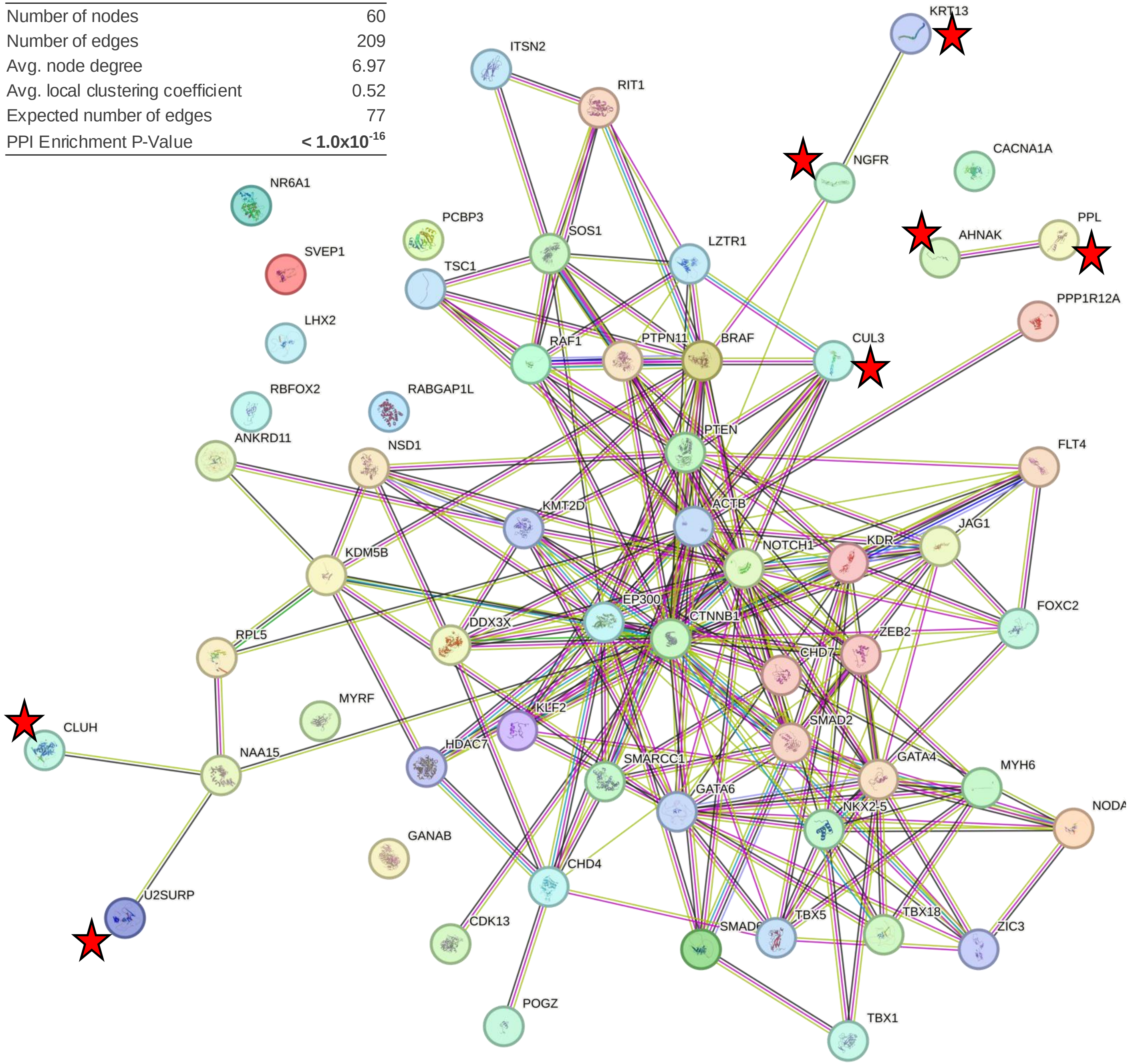

Figure S14 | STRING analysis of 60 significant Genes in CHD

Genes not previously implicated in human CHD that have at least one edge are denoted by red star. Metrics for the network calculated by STRING are provided as the inset table.

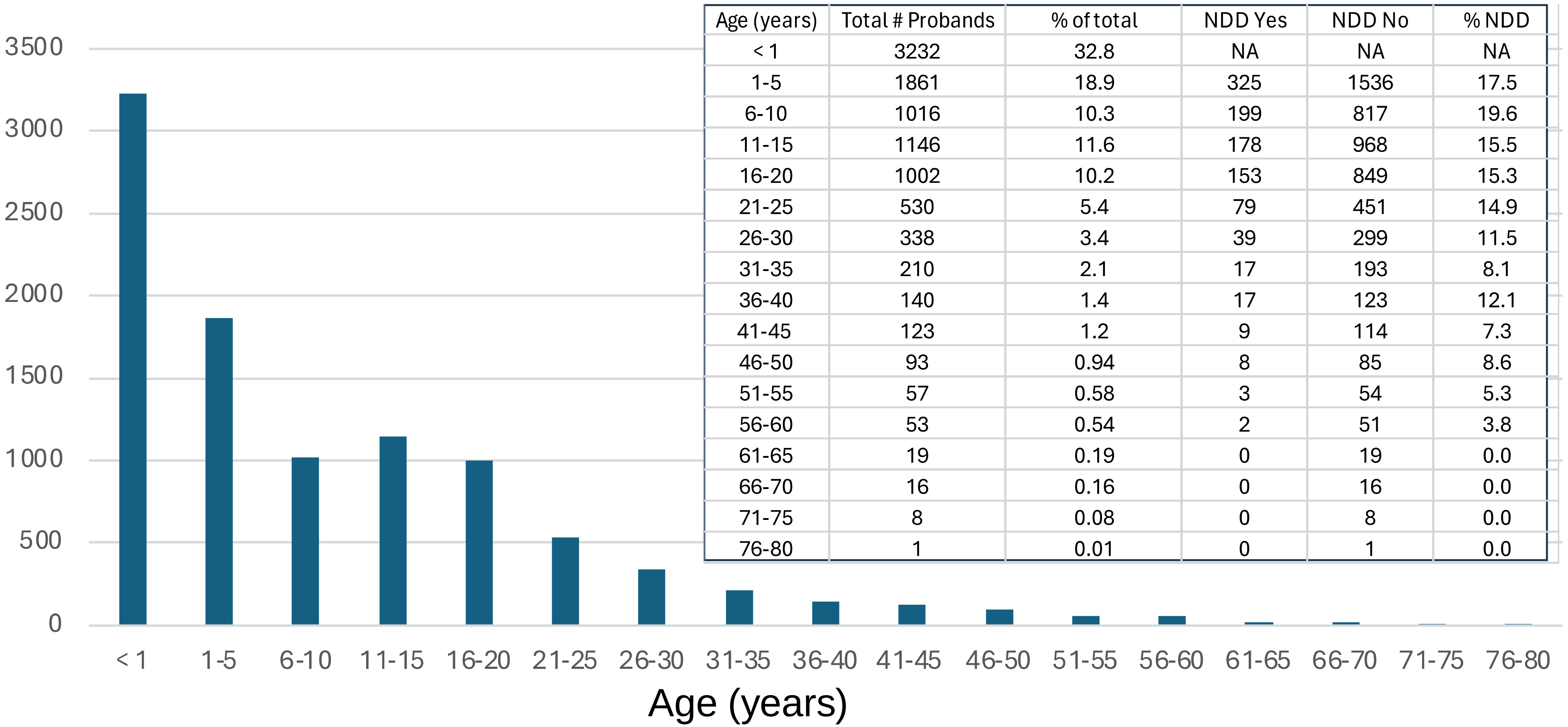

Age (years)	Total # Probands	% of total	NDD Yes	NDD No	% NDD
< 1	3232	32.8	NA	NA	NA
1-5	1861	18.9	325	1536	17.5
6-10	1016	10.3	199	817	19.6
11-15	1146	11.6	178	968	15.5
16-20	1002	10.2	153	849	15.3
21-25	530	5.4	79	451	14.9
26-30	338	3.4	39	299	11.5
31-35	210	2.1	17	193	8.1
36-40	140	1.4	17	123	12.1
41-45	123	1.2	9	114	7.3
46-50	93	0.94	8	85	8.6
51-55	57	0.58	3	54	5.3
56-60	53	0.54	2	51	3.8
61-65	19	0.19	0	19	0.0
66-70	16	0.16	0	16	0.0
71-75	8	0.08	0	8	0.0
76-80	1	0.01	0	1	0.0

Figure S15 | Age at enrollment of PCGC probands. Bar graph shows the # of probands in indicated age groups in the PCGC probands studied. The inset shows the number of probands in each age range, the number with and without NDD and the percentage with NDD. NDD status in all PCGC probands who were at least one year of age at the time of enrollment was based on questionnaire obtained at the time of enrollment. Probands were categorized as having NDD if probands' parents (or adult probands themselves) answered "Yes" to having been told by a physician that the proband had NDD. The prevalence of NDD varied between 14.9% and 19.6% in 5 year windows under age 21, then declines progressively at older ages.

Table S1 | Sanger sequencing validates 100% of 59 tested loss of function mutations identified by MIPseq pipeline

A

Sample	Chr	Pos	GT	Ref	Alt	DP	VAF	Functional	Gene	Sanger
1-08118	2	128317316	0/1	G	T	35	66%	Splicing	MYO7B	Confirmed
1-03823	2	128339517	0/1	C	T	55	65%	Stopgain	MYO7B	Confirmed
1-08223	2	128389238	0/1	AC	A	168	48%	FS_Del	MYO7B	Confirmed
1-15737	3	148750100	0/1	TTC	T	330	53%	FS_Del	HLTF	Confirmed
1-00901	3	148750100	0/1	TTC	T	475	56%	FS_Del	HLTF	Confirmed
1-13821	3	148764039	0/1	G	A	153	44%	Stopgain	HLTF	Confirmed
1-03566	3	148789061	0/1	AT	A	505	43%	FS_Del	HLTF	Confirmed
1-05530	3	148789134	0/1	G	A	374	52%	Stopgain	HLTF	Confirmed
1-08626	5	180030206	0/1	CTGTG	C	425	30%	FS_Del	FLT4	Confirmed
1-05285	5	180030323	0/1	GC	G	49	39%	FS_Del	FLT4	Confirmed
1-02971	5	180036028	0/1	A	G	506	48%	D-Mis	FLT4	Confirmed
1-09295	5	180038338	0/1	CCAGGCTGTGG	C	56	39%	FS_Del	FLT4	Confirmed
1-03349	5	180041122	0/1	T	C	40	45%	D-Mis	FLT4	Confirmed
1-05485	5	180045908	0/1	C	A	18	50%	Stopgain	FLT4	Confirmed
1-00706	5	180046110	0/1	C	T	9	56%	Splicing	FLT4	Confirmed
1-15237	5	180046282	0/1	T	C	503	44%	D-Mis	FLT4	Confirmed
1-00520	5	180046348	0/1	T	G	214	49%	D-Mis	FLT4	Confirmed
1-01663	5	180046697	0/1	C	T	16	75%	D-Mis	FLT4	Confirmed
1-06761	5	180047191	0/1	G	A	168	52%	Stopgain	FLT4	Confirmed
1-08935	5	180047215	0/1	C	T	98	46%	D-Mis	FLT4	Confirmed
1-14795	5	180047215	0/1	C	T	124	48%	D-Mis	FLT4	Confirmed
1-10190	5	180047641	0/1	G	A	110	45%	D-Mis	FLT4	Confirmed
BRZ-1775	5	180047648	0/1	GAAGAA	G	240	68%	FS_Del	FLT4	Confirmed
1-03895	5	180047888	0/1	C	T	749	44%	D-Mis	FLT4	Confirmed
1-02435	5	180048147	0/1	A	G	198	47%	D-Mis	FLT4	Confirmed
1-06610	5	180048862	0/1	TC	T	11	45%	FS_Del	FLT4	Confirmed
1-15059	5	180049754	0/1	C	T	26	46%	D-Mis	FLT4	Confirmed
1-07755	5	180049782	0/1	C	T	155	57%	D-Mis	FLT4	Confirmed
1-10190	5	180050951	0/1	A	G	35	37%	D-Mis	FLT4	Confirmed
1-00947	5	180051062	0/1	C	A	203	63%	Splicing	FLT4	Confirmed
BRZ-2811	5	180052893	0/1	C	G	251	44%	D-Mis	FLT4	Confirmed
BRZ-3067	5	180052945	0/1	C	T	111	46%	D-Mis	FLT4	Confirmed
1-16082	5	180052965	0/1	G	A	282	54%	D-Mis	FLT4	Confirmed
1-15926	5	180052971	0/1	C	T	225	58%	D-Mis	FLT4	Confirmed
1-02100	5	180053185	0/1	C	T	37	41%	D-Mis	FLT4	Confirmed
1-00631	5	180056340	0/1	G	A	119	40%	Stopgain	FLT4	Confirmed

Sample	Chr	Pos	GT	Ref	Alt	DP	VAF	Functional	Gene	Sanger
1-15471	6	157454342	0/1	G	A	209	47%	Splicing	ARID1B	Confirmed
1-07092	8	61654425	0/1	G	A	37	46%	Stopgain	CHD7	Confirmed
1-07890	8	61655543	0/1	C	T	119	52%	Stopgain	CHD7	Confirmed
1-03501	8	61734586	0/1	C	T	248	35%	Stopgain	CHD7	Confirmed
1-06220	8	61777837	0/1	CA	C	414	53%	FS_Del	CHD7	Confirmed
1-06829	9	139397784	0/1	T	C	499	54%	Splicing	NOTCH1	Confirmed
1-09990	9	139402743	0/1	C	T	254	46%	Stopgain	NOTCH1	Confirmed
1-06816	9	140638544	0/1	T	C	26	58%	Splicing	EHMT1	Confirmed
1-08657	12	6702648	0/1	GATGA	G	27	63%	FS_Del	CHD4	Confirmed
1-04372	12	49415825	0/1	C	A	284	52%	Splicing	KMT2D	Confirmed
1-15501	12	49418395	0/1	G	A	41	51%	Stopgain	KMT2D	Confirmed
1-08230	12	49427999	0/1	G	A	112	43%	Stopgain	KMT2D	Confirmed
1-14957	12	49443635	0/1	TAG	T	88	69%	FS_Del	KMT2D	Confirmed
1-03907	14	23866027	0/1	C	A	40	65%	Splicing	MYH6	Confirmed
1-07682	14	23869929	0/1	C	A	11	45%	Stopgain	MYH6	Confirmed
1-12407	18	19751617	0/1	ACT	A	37	46%	FS_Del	GATA6	Confirmed
1-13425	19	13563745	0/1	CT	C	88	48%	FS_Del	CACNA1A	Confirmed
1-07732	20	40161867	0/1	C	A	212	42%	Stopgain	CHD6	Confirmed
1-15334	22	21337352	0/1	C	CA	34	62%	FS_Ins	LZTR1	Confirmed
1-06785	22	21337379	0/1	G	A	150	49%	Splicing	LZTR1	Confirmed
1-14931	22	21350307	0/1	TC	T	230	47%	FS_Del	LZTR1	Confirmed
1-15624	22	24509694	0/1	CA	C	22	50%	FS_Del	CABIN1	Confirmed
1-17087	22	41568624	0/1	AAG	A	413	53%	FS_Del	EP300	Confirmed

B

Type	Sanger Validation			
	Pass	Fail	Total	%
SNV	29	0	29	100
Indel	30	0	30	100
**Total**	**59**	**0**	**59**	**100**

Sanger sequencing validation results on loss of function mutations identified by MIPseq. **[A]** Table of individual mutations sorted by genomic position. The Sanger column indicates if a mutation was confirmed by Sanger sequencing. **[B]** Table shows the number of mutations that pass or fail Sanger validation stratified by single nucleotide variant (SNV) versus insertion or deletion (indel) variant. GT is "genotype", Ref is "reference allele (hg19)", Alt is "variant allele", DP is "depth of coverage at locus", VAF is "fraction of variant-supporting divided by total reads", Functional is "predicted variant impact on encoded protein", and Sanger denotes if the variant was validated by Sanger sequencing.

Table S2 | General characteristics of CHD probands and controls

Characteristic	MIPseq Probands (n = 5,929)		WES Probands (n = 5,626)		CHD Probands (n = 11,555)		gnomAD Controls (n = 135,743)	
**Family Structure**	#	(%)	#	(%)	#	(%)	#	(%)
Singleton	5,929	(100.0)	1,739	(30.9)	7,668	(66.4)	135,743	(100.0)
Trio	0	(0.0)	3,887	(69.1)	3,887	(33.6)	0	(0.0)
**Sex**	#	(%)	#	(%)	#	(%)	#	(%)
Male	3,151	(53.1)	3,268	(58.1)	6,419	(55.6)	73,782	(54.4)
Female	2,778	(46.9)	2,358	(41.9)	5,136	(44.4)	61,961	(45.6)
**Ethnicity**	#	(%)	#	(%)	#	(%)	#	(%)
African	454	(7.7)	348	(6.2)	802	(6.9)	10,291	(7.6)
Asian	400	(6.7)	331	(5.9)	731	(6.3)	25,264	(18.6)
European	3,105	(52.4)	4,004	(71.2)	7,109	(61.5)	79,016	(58.2)
Hispanic	1,438	(24.3)	557	(9.9)	1,995	(17.3)	17,634	(13.0)
Unknown	532	(9.0)	386	(6.9)	918	(7.9)	3,538	(2.6)

Controls from the gnomAD database include samples with both WES and WGS, but exclude samples from the TOPmed database. Ethnicities were based on categories provided by gnomAD consortium. WES ethnicity was determined by PCA using HapMap samples as controls. MIPseq ethnicity was derived from self-report when necessary. Percentages sum to 100% within each category.

Table S3 | Cardiac and extracardiac phenotypes in 11,555 CHD probands

A

Cardiac Lesion Classification		# Trios		# Singletons		All Cases	
Conotruncal Defects†	(CTD)	631	(16.2%)	1,811	(23.6%)	2,442	(21.1%)
Tetralogy of Fallot	(TOF)	754	(19.4%)	1,044	(13.6%)	1,798	(15.6%)
Laterality Defects*	(LAT)	646	(16.6%)	1,322	(17.2%)	1,968	(17.0%)
Left-Ventricular Obstruction††	(LVO)	504	(13.0%)	989	(12.9%)	1,493	(12.9%)
Hypoplastic Left Heart Syndrome	(HLHS)	560	(14.4%)	516	(6.7%)	1,076	(9.3%)
Atrial Septal Defect	(ASD)	216	(5.6%)	494	(6.4%)	710	(6.1%)
Atrioventricular Canal Defect	(AVC)	160	(4.1%)	303	(4.0%)	463	(4.0%)
Other **	(OTH)	404	(10.4%)	1,059	(13.8%)	1,463	(12.7%)
Unknown	(UNK)	12	(0.3%)	130	(1.7%)	142	(1.2%)
**Total**		**3,887**	**(100.0%)**	**7,668**	**(100.0%)**	**11,555**	**(100.0%)**

B

EC and NDD Classification		# Trios		# Singletons		All Cases	
Isolated CHD	(Isolated)	1,238	(31.8%)	3,379	(44.1%)	4,617	(40.0%)
CHD + Extracardiac Abnormality	(EC)	1,014	(26.1%)	918	(12.0%)	1,932	(16.7%)
CHD + Neurodevelopmental Deficit	(NDD)	232	(6.0%)	324	(4.2%)	556	(4.8%)
CHD + Both NDD & EC	(NDD & EC)	262	(6.7%)	211	(2.8%)	473	(4.1%)
No EC & NDD Data	(Unknown)	1,141	(29.4%)	2,836	(37.0%)	3,977	(34.4%)
**Total**		**3,887**	**(100.0%)**	**7,668**	**(100.0%)**	**11,555**	**(100.0%)**

Number of proportion of 11,555 CHD probands stratified by **[A]** Cardiac lesions and **[B]** extracardiac (EC) or neurodevelopmental deficit (NDD) status. In **[A]**, CHD probands are identified by the major cardiac lesion present as described in Methods. In **[B]**, extracardiac abnormalities (EC) and neurodevelopmental deficits (NDD) were based on assessment by PCGC clinicians as described in Methods. Those assessed with neither were designated "isolated CHD". Those assessed to have EC, but not NDD, were designated "EC". Those assessed to have NDD, but not EC, were designated "NDD". Those assessed to have both EC and NDD, were designated "EC & NDD". Those that were not assessed or were too young for accurate NDD assessment were designated "Unknown". †CTD without TOF; ††LVO without HLHS; *LAT include probands with Heterotaxy and D-Transposition of the Great Arteries; **Other (OTH) includes probands with defects that do not fall into the above categories. See Methods for additional details.

Table S4 | Enrichment of damaging *de novo* mutations in 3,887 WES Trios

A

Functional Class	All Exome-wide Genes (n = 19,000)						
	**Obs**	**Freq**	**Exp**	**Freq**	**Enrich**	**P-Val**	**% Explained**
Synonymous	1,064	2.7E-01	1,219	3.1E-01	0.87	1.00E+00	NS
Tolerated Mis	2,155	5.5E-01	2,233	5.7E-01	0.97	9.51E-01	NS
Damaging Mis	650	1.7E-01	481	1.2E-01	1.35	**1.24E-13**	4.36%
Loss of Function	558	1.4E-01	374	9.6E-02	1.49	**4.63E-19**	4.73%
Protein-Damaging	1,208	3.1E-01	855	2.2E-01	1.41	**3.14E-30**	9.09%

B

Functional Class	MIPseq Panel Genes (n = 248)						
	**Obs**	**Freq**	**Exp**	**Freq**	**Enrich**	**P-Val**	**% Explained**
Synonymous	30	7.7E-03	32.2	8.3E-03	0.93	6.74E-01	NS
Tolerated Mis	86	2.2E-02	51.3	1.3E-02	1.68	**6.10E-06**	0.89%
Damaging Mis	134	3.4E-02	21.9	5.6E-03	6.12	**7.83E-59**	2.88%
Loss of Function	154	4.0E-02	10.8	2.8E-03	14.27	**8.56E-118**	3.68%
Protein-Damaging	288	7.4E-02	32.7	8.4E-03	8.81	**1.43E-163**	6.57%

Tables show burden of DNMs from Poisson test in 3,887 WES trios sorted by variant functional consequence using **[A]** all 19,000 exome-wide genes, and **[B]** 248 targeted genes. Abbreviations: 'Obs' is the number of observed DNMs; 'Exp' is the expected number of DNMs based on gene mutability; 'Freq' denotes the frequency (fraction) of observed or expected DNM in each mutation class. 'Enrich' denotes enrichment, ratio of the observed:expected frequency of DNMs in each variant class; 'P-Val' is the Poisson p-value; '% Explained' is the excess percentage of probands with mutations in each variant class that significantly exceeds expectation. P-values < 0.05 are colored in red.

Table S5 | Enrichment of damaging DNMs in 2,686 WES trios after removal of 1,213 previously studied probands

A

Functional Class	All Genes (n ≈ 19,000)						
	Obs	Freq	Exp	Freq	Enrich	P-Val	% Explained
Synonymous	775	2.9E-01	842	3.1E-01	0.92	9.91E-01	NS
Tolerated Mis	1,524	5.7E-01	1,543	5.7E-01	0.99	6.87E-01	NS
Damaging Mis	439	1.6E-01	332	1.2E-01	1.32	**1.25E-08**	3.98%
Loss of Function	408	1.5E-01	258	9.6E-02	1.58	**6.05E-18**	5.57%
Protein-Damaging	847	3.2E-01	591	2.2E-01	1.43	**2.29E-23**	9.55%

B

Functional Class	MIPSeq Panel Genes (n = 248)						
	Obs	Freq	Exp	Freq	Enrich	P-Val	% Explained
Synonymous	27	1.0E-02	22	8.3E-03	1.21	1.81E-01	0.18%
Tolerated Mis	54	2.0E-02	35	1.3E-02	1.52	**2.24E-03**	0.69%
Damaging Mis	85	3.2E-02	15	5.6E-03	5.62	**2.30E-35**	2.60%
Loss of Function	108	4.0E-02	7	2.8E-03	14.49	**7.87E-84**	3.74%
Protein-Damaging	193	7.2E-02	23	8.4E-03	8.54	**5.20E-108**	6.34%

Tables show burden of DNMs from Poisson test in 2,686 WES trios, removing 1,213 probands present in cohort used to select panel genes, stratified by variant functional consequence using: **[A]** all 19,000 exome-wide genes, and **[B]** 248 panel genes. Abbreviations as defined previously. 'P-Val' is the Poisson p-value. P-values < 0.05 are colored in red.

Table S6 | Enrichment of damaging ultra-rare transmitted or unphased variants in 11,555 CHD probands versus 135,743 gnomAD controls

A

Functional	CHD Probands		gnomAD		Fisher's Exact Test	
**All 248 Panel Genes**	Obs	Freq	Obs	Freq	Enrich	P-Val
Synonymous	8,961	3.89E-01	104,303	3.84E-01	1.01	7.12E-02
Tolerated Missense	10,807	4.69E-01	136,759	5.04E-01	0.93	1.00E+00
Damaging Missense	3,603	1.57E-01	38,152	1.41E-01	1.11	**2.64E-11**
Loss of Function	737	3.20E-02	5,875	2.17E-02	1.48	**3.21E-22**
Protein-Damaging	4,340	1.89E-01	44,027	1.62E-01	1.16	**1.51E-24**

B

Functional	CHD Proband		gnomAD		Fisher's Exact Test	
**28 LOF-Tolerant & Not Previously Implicated in CHD**	Obs	Freq	Obs	Freq	Enrich	P-Val
Synonymous	998	4.35E-02	11,783	4.34E-02	1.00	4.84E-01
Tolerated Missense	1,702	7.42E-02	21,909	8.07E-02	0.92	1.00E+00
Damaging Missense	425	1.85E-02	5,088	1.87E-02	0.99	6.02E-01
Loss of Function	172	7.50E-03	2,128	7.85E-03	0.96	7.30E-01
Protein-Damaging	597	2.60E-02	7,216	2.66E-02	0.98	7.03E-01

C

Functional	CHD Probands		gnomAD		Fisher's Exact Test	
**220 Known or Candidate CHD Genes**	Obs	Freq	Obs	Freq	Enrich	P-Val
Synonymous	7,963	3.46E-01	92,520	3.41E-01	1.01	6.36E-02
Tolerated Missense	9,105	3.95E-01	114,850	4.23E-01	0.93	1.00E+00
Damaging Missense	3,178	1.38E-01	33,064	1.22E-01	1.13	**7.64E-13**
Loss of Function	565	2.45E-02	3,747	1.38E-02	1.78	**2.95E-33**
Protein-Damaging	3,743	1.63E-01	36,811	1.36E-01	1.20	**4.01E-29**

Enrichment of ultra-rare (MAF < 10^{-5}) TUVs in 11,555 CHD probands vs gnomAD controls for **[A]** all 248 panel genes; **[B]** set of 28 panel genes with pLI < 0.9, Missense Z-Score < 0.2, and no prior evidence for CHD pathogenesis; **[C]** 220 MIPS panel genes that are known or candidates for CHD pathogenicity. 'P-Val' is the p-value from a two-sided Fisher's Exact test. P-values < 0.05 are colored in red.

Table S7 | Sixty genes significantly associated with CHD pathogenesis in meta-analysis of Damaging DNMs and ultra-rare TUVs

A

		Annotation			Poisson Test of DNMs in CHD Probands							Case-Control of Ultra-Rare TUVs											Meta-Analysis	
		Gene	Intolerance									CHD Probands				gnomAD Controls				Fisher's Exact				
			pLI	MisZ	LOF	D-mis	Dmg	Exp	Enrich	P-Val	FDR	LOF	D-mis	Dmg	Freq	LOF	D-mis	Dmg	Freq	Enrich	P-Val	FDR	P-Val	FDR
□	1	KMT2D	1.00	3.7	21	6	27	0.61	44.5	7.2E-35	1.79E-32	12	96	108	5.0E-03	35	796	831	3.3E-03	1.5	7.4E-05	2.30E-03	4.54E-38	1.09E-32
□	2	PTPN11	1.00	3.1	0	12	12	0.11	109.2	5.8E-21	4.81E-19	0	36	36	1.6E-03	2	110	112	4.1E-04	3.9	4.1E-10	2.55E-08	1.72E-29	9.76E-26
□	3	CHD7	1.00	3.2	16	4	20	0.37	54.7	5.2E-28	6.47E-26	18	53	71	3.1E-03	9	594	603	2.3E-03	1.3	1.4E-02	1.19E-01	5.45E-28	1.41E-24
□	4	NOTCH1	1.00	3.4	7	3	10	0.46	21.9	7.3E-11	4.51E-09	30	113	143	6.5E-03	9	885	894	3.5E-03	1.8	2.2E-10	1.81E-08	3.29E-19	8.65E-17
□	5	MYH6	0.00	0.9	1	4	5	0.46	10.9	1.2E-04	1.26E-03	15	167	182	8.8E-03	105	1,120	1,225	4.5E-03	2.0	7.0E-15	1.73E-12	4.95E-16	5.44E-14
	6	JAG1	1.00	3.2	5	1	6	0.23	26.4	1.6E-07	6.45E-06	20	39	59	2.6E-03	8	277	285	1.1E-03	2.4	1.5E-08	7.54E-07	4.54E-14	2.81E-12
	7	TBX5	1.00	1.2	4	1	5	0.07	74.5	1.1E-08	5.31E-07	4	18	22	1.0E-03	3	100	103	3.8E-04	2.7	1.3E-04	3.69E-03	9.72E-12	3.10E-10
	8	NODAL	0.97	1.0	0	0	0	0.04	0.0	1.0E+00	1.00E+00	20	9	29	1.4E-03	7	36	43	1.8E-04	7.6	9.9E-14	1.22E-11	7.57E-10	1.40E-08
▲	9	SMAD2	1.00	3.7	1	3	4	0.09	42.6	3.0E-06	8.90E-05	3	14	17	7.4E-04	4	61	65	2.4E-04	3.1	1.9E-04	4.59E-03	3.87E-09	6.54E-08
□	10	FLT4	1.00	2.8	2	2	4	0.18	21.9	4.0E-05	5.48E-04	22	27	49	2.2E-03	13	267	280	1.1E-03	2.1	1.2E-05	4.19E-04	5.06E-09	1.19E-07
□	11	RBFOX2	1.00	2.6	3	0	3	0.03	90.6	5.9E-06	1.22E-04	4	4	8	3.7E-04	2	18	20	8.1E-05	4.6	1.3E-03	1.77E-02	4.47E-08	4.98E-07
	12	GATA4	0.49	0.7	4	0	4	0.16	24.6	2.6E-05	3.98E-04	3	41	44	2.2E-03	7	253	260	1.3E-03	1.7	1.1E-03	1.62E-02	1.67E-07	1.62E-06
▲	13	MYRF	1.00	3.3	1	2	3	0.05	59.7	2.0E-05	3.36E-04	6	1	7	3.4E-04	21	3	24	1.0E-04	3.4	8.9E-03	8.83E-02	9.15E-07	6.35E-06
▲	14	ACTB	0.99	5.0	1	2	3	0.12	24.4	2.8E-04	2.79E-03	1	7	8	3.6E-04	1	15	16	6.5E-05	5.5	4.9E-04	9.44E-03	9.78E-07	1.07E-05
	15	KDM5B	0.00	1.8	4	1	5	0.22	23.2	3.2E-06	8.90E-05	16	24	40	1.8E-03	84	325	409	1.5E-03	1.1	2.3E-01	6.81E-01	3.76E-06	2.47E-05
	16	NSD1	1.00	3.4	4	2	6	0.37	16.2	2.6E-06	8.90E-05	0	56	56	2.5E-03	16	614	630	2.3E-03	1.1	3.0E-01	8.09E-01	4.12E-06	3.82E-05
	17	GATA6	1.00	1.3	4	1	5	0.23	21.7	4.4E-06	9.99E-05	3	33	36	1.8E-03	1	328	329	1.7E-03	1.1	3.5E-01	8.69E-01	8.11E-06	6.16E-05
	18	CDK13	0.91	2.3	1	1	2	0.07	26.7	2.7E-03	1.89E-02	5	8	13	6.3E-04	16	25	41	1.9E-04	3.3	6.2E-04	1.10E-02	1.25E-05	9.35E-05
	19	RAF1	0.85	2.5	0	4	4	0.10	40.5	3.7E-06	9.13E-05	0	8	8	3.7E-04	12	137	149	5.5E-04	0.7	9.1E-01	1.00E+00	1.94E-05	1.38E-04
	20	ZEB2	1.00	3.9	2	1	3	0.07	41.0	6.2E-05	7.69E-04	1	9	10	4.5E-04	1	69	70	2.6E-04	1.7	8.3E-02	4.24E-01	2.26E-05	1.85E-04
	21	SOS1	1.00	3.0	1	3	4	0.14	29.5	1.3E-05	2.40E-04	1	13	14	6.2E-04	11	139	150	5.6E-04	1.1	3.8E-01	8.87E-01	2.32E-05	2.28E-04
	22	LZTR1	0.00	0.6	0	3	3	0.07	43.2	5.3E-05	6.93E-04	18	17	35	1.6E-03	225	110	335	1.3E-03	1.2	1.3E-01	5.26E-01	2.90E-05	2.78E-04
▲	23	NAA15	1.00	3.8	3	0	3	0.05	64.5	1.6E-05	2.87E-04	1	2	3	1.3E-04	12	10	22	9.5E-05	1.4	4.0E-01	8.98E-01	3.06E-05	3.27E-04
	24	RIT1	0.13	2.1	0	2	2	0.04	55.9	6.2E-04	5.83E-03	2	7	9	4.0E-04	15	26	41	1.5E-04	2.6	1.3E-02	1.17E-01	3.95E-05	3.86E-04
	25	ANKRD11	1.00	-0.6	2	0	2	0.09	21.6	4.0E-03	2.44E-02	8	0	8	3.9E-04	7	15	22	9.4E-05	4.2	1.9E-03	2.50E-02	4.98E-05	4.56E-04
△	26	CTNNB1	1.00	3.8	3	0	3	0.06	47.8	3.9E-05	5.48E-04	0	5	5	2.2E-04	1	50	51	1.9E-04	1.2	4.5E-01	9.44E-01	7.84E-05	5.60E-04
△	27	KLF2	0.56	2.2	1	1	2	0.01	157.8	8.0E-05	8.97E-04	2	0	2	1.2E-04	5	2	7	6.5E-05	1.9	3.3E-01	8.42E-01	1.12E-04	7.00E-04
▲	28	POGZ	1.00	3.5	2	1	3	0.08	38.5	7.4E-05	8.77E-04	2	4	6	2.7E-04	6	51	57	2.1E-04	1.2	3.7E-01	8.84E-01	1.16E-04	8.33E-04
▲	29	SMARCC1	1.00	2.4	0	0	0	0.07	0.0	1.0E+00	1.00E+00	12	4	16	7.3E-04	15	22	37	1.6E-04	4.6	5.4E-06	2.23E-04	2.33E-04	1.08E-03
★	30	CACNA1A	1.00	5.8	1	3	4	0.66	6.1	4.6E-03	2.70E-02	3	80	83	3.9E-03	32	648	680	3.0E-03	1.3	1.3E-02	1.17E-01	2.98E-04	1.38E-03

★ Novel   △ Novel in human   ▲ 1st statistical support   □ Previously significant in PCGC   GWS   FDR < 0.05

Table S7 [Continued] | Sixty genes significantly associated with CHD pathogenesis in meta-analysis of Damaging DNMs and ultra-rare TUVs

B

		Annotation			Poisson Test of DNMs in CHD Probands							Case-Control of Ultra-Rare TUVs											Meta-Analysis	
		Gene	Intolerance									CHD Probands				gnomAD Controls				Fisher's Exact				
			pLI	MisZ	LOF	D-mis	Dmg	Exp	Enrich	P-Val	FDR	LOF	D-mis	Dmg	Freq	LOF	D-mis	Dmg	Freq	Enrich	P-Val	FDR	P-Val	FDR
★	31	NR6A1	0.98	1.8	1	2	3	0.11	27.1	2.1E-04	**2.15E-03**	1	14	15	6.7E-04	4	194	198	7.9E-04	0.9	7.6E-01	1.00E+00	6.17E-04	**1.93E-03**
★	32	AHNAK	0.90	-3.0	1	1	2	0.06	31.6	1.9E-03	**1.59E-02**	10	2	12	5.3E-04	84	3	87	3.3E-04	1.6	9.1E-02	4.24E-01	6.85E-04	**2.50E-03**
★	33	PCBP3	0.84	2.0	1	0	1	0.03	39.9	2.5E-02	1.04E-01	4	2	6	3.2E-04	8	10	18	7.8E-05	4.1	7.2E-03	7.75E-02	1.01E-03	**3.27E-03**
□	34	SMAD6	0.00	-0.6	1	0	1	0.13	7.7	1.2E-01	3.00E-01	29	29	58	3.3E-03	58	271	329	2.0E-03	1.6	9.5E-04	**1.57E-02**	1.06E-03	**4.03E-03**
▲	35	GANAB	1.00	2.2	1	1	2	0.19	10.4	1.6E-02	8.08E-02	2	37	39	1.7E-03	24	291	315	1.2E-03	1.5	1.4E-02	1.19E-01	1.15E-03	**4.80E-03**
▲	36	PPP1R12A	1.00	2.6	1	1	2	0.06	31.7	1.9E-03	**1.59E-02**	2	2	4	1.8E-04	7	13	20	9.0E-05	2.0	1.7E-01	5.99E-01	1.21E-03	**5.57E-03**
▲	37	RPL5	1.00	1.9	2	0	2	0.04	55.4	6.4E-04	**5.83E-03**	1	1	2	9.1E-05	0	26	26	9.6E-05	1.0	6.3E-01	1.00E+00	1.45E-03	**6.43E-03**
★	38	CLUH	0.99	1.8	0	3	3	0.25	11.9	2.2E-03	**1.66E-02**	0	42	42	1.9E-03	31	393	424	1.7E-03	1.1	2.6E-01	7.54E-01	2.06E-03	**7.57E-03**
	39	CHD4	1.00	6.3	0	4	4	0.42	9.6	9.1E-04	**8.06E-03**	3	31	34	1.5E-03	23	421	444	1.7E-03	0.9	7.3E-01	1.00E+00	2.30E-03	**8.77E-03**
▲	40	TBX18	1.00	0.1	1	0	1	0.09	11.6	8.3E-02	2.42E-01	3	19	22	1.2E-03	5	154	159	6.2E-04	2.0	4.5E-03	5.03E-02	2.43E-03	**9.97E-03**
★	41	U2SURP	1.00	4.1	1	1	2	0.07	29.4	2.2E-03	**1.66E-02**	1	2	3	1.3E-04	3	19	22	9.7E-05	1.4	3.9E-01	8.91E-01	3.00E-03	**1.14E-02**
★	42	LHX2	0.99	2.3	2	0	2	0.07	28.6	2.3E-03	**1.71E-02**	0	5	5	2.4E-04	2	51	53	2.1E-04	1.1	4.7E-01	9.56E-01	3.74E-03	**1.30E-02**
▲	43	PTEN	0.26	3.5	2	0	2	0.08	23.9	3.3E-03	**2.23E-02**	2	5	7	3.1E-04	16	50	66	2.5E-04	1.3	3.4E-01	8.43E-01	3.86E-03	**1.46E-02**
★	44	RABGAP1L	0.00	1.1	1	1	2	0.07	30.6	2.1E-03	**1.64E-02**	6	1	7	3.3E-04	46	38	84	3.5E-04	0.9	6.4E-01	1.00E+00	4.34E-03	**1.63E-02**
	45	DDX3X	1.00	4.3	0	1	1	0.04	25.1	3.9E-02	1.47E-01	2	0	2	1.2E-04	1	0	1	6.2E-06	19.8	2.4E-02	1.62E-01	4.40E-03	**1.79E-02**
△	46	HDAC7	1.00	2.8	1	0	1	0.07	13.8	7.0E-02	2.12E-01	6	5	11	5.3E-04	22	33	55	2.3E-04	2.4	1.3E-02	1.17E-01	4.95E-03	**1.96E-02**
	47	BRAF	1.00	3.7	0	2	2	0.09	23.0	3.6E-03	**2.27E-02**	0	6	6	2.7E-04	8	47	55	2.3E-04	1.2	4.3E-01	9.32E-01	5.12E-03	**2.14E-02**
▲	48	ZIC3	0.92	2.5	0	0	0	0.03	0.0	1.0E+00	1.00E+00	5	4	9	6.4E-04	6	14	20	1.3E-04	4.9	4.3E-04	**9.44E-03**	5.53E-03	**2.31E-02**
★	49	KRT13	0.00	-0.5	0	2	2	0.08	26.4	2.7E-03	**1.89E-02**	3	18	21	9.3E-04	25	235	260	9.8E-04	1.0	6.2E-01	1.00E+00	5.57E-03	**2.49E-02**
	50	NKX2-5	0.95	0.2	0	0	0	0.08	0.0	1.0E+00	1.00E+00	11	13	24	1.2E-03	25	98	123	5.2E-04	2.3	4.6E-04	**9.44E-03**	5.79E-03	**2.66E-02**
▲	51	ITSN2	0.00	1.1	0	2	2	0.13	15.4	7.8E-03	**4.19E-02**	6	21	27	1.2E-03	127	135	262	9.9E-04	1.2	2.1E-01	6.64E-01	5.84E-03	**2.82E-02**
	52	TBX1	0.84	0.7	1	1	2	0.09	23.3	3.5E-03	**2.27E-02**	4	10	14	8.1E-04	35	127	162	8.3E-04	1.0	5.8E-01	1.00E+00	6.57E-03	**3.00E-02**
★	53	SVEP1	1.00	1.9	1	0	1	0.31	3.2	2.7E-01	5.88E-01	9	64	73	3.2E-03	61	518	579	2.2E-03	1.4	3.3E-03	**3.88E-02**	6.65E-03	**3.17E-02**
★	54	NGFR	0.10	1.4	0	2	2	0.09	21.9	3.9E-03	**2.44E-02**	1	11	12	5.9E-04	10	139	149	5.9E-04	1.0	5.4E-01	9.84E-01	6.96E-03	**3.35E-02**
▲	55	TSC1	1.00	2.3	1	1	2	0.17	11.6	1.3E-02	6.82E-02	0	36	36	1.6E-03	9	353	362	1.3E-03	1.2	1.8E-01	6.04E-01	8.21E-03	**3.55E-02**
★	56	CUL3	1.00	4.8	1	0	1	0.08	11.8	8.1E-02	2.40E-01	2	9	11	4.9E-04	11	49	60	2.3E-04	2.1	2.4E-02	1.62E-01	9.57E-03	**3.97E-02**
▲	57	KDR	1.00	1.0	0	0	0	0.13	0.0	1.0E+00	1.00E+00	10	21	31	1.7E-03	26	210	236	8.7E-04	1.9	1.1E-03	**1.62E-02**	1.11E-02	**4.20E-02**
▲	58	FOXC2	0.13	-0.3	1	1	2	0.11	18.7	5.3E-03	**3.01E-02**	0	17	17	8.9E-04	6	187	193	1.0E-03	0.9	7.5E-01	1.00E+00	1.22E-02	**4.44E-02**
★	59	PPL	0.00	-3.7	1	1	2	0.10	19.1	5.1E-03	**2.94E-02**	17	15	32	1.5E-03	230	245	475	1.8E-03	0.8	8.6E-01	1.00E+00	1.30E-02	**4.69E-02**
▲	60	EP300	1.00	2.0	2	1	3	0.36	8.3	6.0E-03	**3.30E-02**	2	38	40	1.8E-03	13	547	560	2.1E-03	0.9	8.1E-01	1.00E+00	1.45E-02	**4.95E-02**

★ Novel △ Novel in human ▲ 1st statistical support □ PCGC Previously Established GWS FDR < 0.05

Table S8 | Independent contribution of *de novo* and transmitted LOF and D-Mis mutations to the sixty significantly enriched genes

A

	Annotation			Loss of Function											D-Mis										
		Intolerance		Poisson of DNMs in Trios				Case-Control of Ultra-Rare TUVs						Meta-Analysis	Poisson of DNMs in Trios				Case-Control of Ultra-Rare TUVs						Meta-Analysis
								CHD		gnomAD		Fisher's Exact							CHD		gnomAD		Fisher's Exact		
	Gene	pLI	MisZ	Obs	Exp	Enrich	FDR	Obs	AF	Obs	AF	Enrich	FDR	FDR	Obs	Exp	Enrich	FDR	Obs	AF	Obs	AF	Enrich	FDR	FDR
□	KMT2D	1.00	3.7	21	0.17	126.8	**1.65E-34**	12	5.5E-04	35	1.5E-04	3.6	**1.24E-02**	**2.70E-34**	6	0.44	13.6	**5.84E-04**	96	4.4E-03	796	3.1E-03	1.4	5.53E-02	**5.04E-06**
□	PTPN11	1.00	3.1	0	0.02	0.0	1.00E+00	0	0.0E+00	2	8.3E-06	0.0	1.00E+00	8.03E-01	12	0.09	139.1	**8.16E-20**	36	1.6E-03	110	4.1E-04	3.9	**3.40E-08**	**1.10E-25**
□	CHD7	1.00	3.2	16	0.10	152.5	**1.16E-27**	18	7.9E-04	9	3.9E-05	20.5	**1.29E-11**	**7.82E-37**	4	0.26	15.4	**5.52E-03**	53	2.3E-03	594	2.3E-03	1.0	1.00E+00	**6.41E-03**
□	NOTCH1	1.00	3.4	7	0.07	102.0	**1.11E-10**	30	1.4E-03	9	4.0E-05	34.3	**6.40E-22**	**4.36E-31**	3	0.39	7.7	7.90E-02	113	5.1E-03	885	3.5E-03	1.5	**1.29E-02**	**2.80E-04**
□	MYH6	0.00	0.9	1	0.05	18.3	2.17E-01	15	7.2E-04	105	3.9E-04	1.9	2.22E-01	9.88E-02	4	0.41	9.9	**1.83E-02**	167	8.1E-03	1,120	4.1E-03	2.0	**1.64E-11**	**4.06E-11**
	JAG1	1.00	3.2	5	0.04	123.9	**5.34E-08**	20	8.8E-04	8	3.3E-05	26.3	**7.39E-14**	**9.65E-21**	1	0.19	5.4	6.59E-01	39	1.7E-03	277	1.0E-03	1.6	8.56E-02	**4.02E-02**
	TBX5	1.00	1.2	4	0.01	297.4	**5.58E-08**	4	1.8E-04	3	1.2E-05	15.0	**2.27E-02**	**1.12E-09**	1	0.05	18.6	2.94E-01	18	8.3E-04	100	3.7E-04	2.2	7.65E-02	**1.61E-02**
	NODAL	0.97	1.0	0	0.01	0.0	1.00E+00	20	9.7E-04	7	3.0E-05	32.1	**1.13E-14**	**1.25E-12**	0	0.03	0.0	1.00E+00	9	4.3E-04	36	1.5E-04	2.8	1.55E-01	2.69E-01
▲	SMAD2	1.00	3.7	1	0.02	44.8	1.45E-01	3	1.3E-04	4	1.6E-05	8.0	1.84E-01	**3.56E-02**	3	0.07	41.9	**2.57E-03**	14	6.1E-04	61	2.3E-04	2.7	6.00E-02	**3.10E-05**
□	FLT4	1.00	2.8	2	0.05	43.2	**1.52E-02**	22	9.8E-04	13	5.5E-05	18.0	**1.09E-13**	**1.53E-14**	2	0.14	14.7	8.39E-02	27	1.2E-03	267	1.0E-03	1.2	8.18E-01	**4.78E-02**
□	RBFOX2	1.00	2.6	3	0.02	149.0	**4.15E-05**	4	1.9E-04	2	9.1E-06	20.3	**1.71E-02**	**4.07E-07**	0	0.01	0.0	1.00E+00	4	1.9E-04	18	7.3E-05	2.5	5.45E-01	4.84E-01
	GATA4	0.49	0.7	4	0.01	313.7	**5.41E-08**	3	1.5E-04	7	3.9E-05	3.9	4.50E-01	**3.50E-08**	0	0.15	0.0	1.00E+00	41	2.1E-03	253	1.3E-03	1.7	7.65E-02	1.92E-01
▲	MYRF	1.00	3.3	1	0.03	32.0	1.74E-01	6	2.9E-04	21	8.7E-05	3.4	1.84E-01	**4.21E-02**	2	0.02	105.4	**5.52E-03**	1	4.9E-05	3	1.4E-05	3.6	9.33E-01	**4.36E-03**
▲	ACTB	0.99	5.0	1	0.01	130.2	8.25E-02	1	4.5E-05	1	4.1E-06	11.0	8.06E-01	8.28E-02	2	0.12	17.4	6.91E-02	7	3.2E-04	15	6.1E-05	5.1	5.53E-02	**1.21E-03**
	KDM5B	0.00	1.8	4	0.08	47.6	**5.16E-05**	16	7.1E-04	84	3.2E-04	2.2	7.22E-02	**2.02E-06**	1	0.13	7.6	5.26E-01	24	1.1E-03	325	1.2E-03	0.9	1.00E+00	4.29E-01
	NSD1	1.00	3.4	4	0.09	46.8	**5.16E-05**	0	0.0E+00	16	6.5E-05	0.0	1.00E+00	**2.19E-04**	2	0.28	7.0	2.12E-01	56	2.5E-03	614	2.3E-03	1.1	8.59E-01	1.70E-01
	GATA6	1.00	1.3	4	0.02	259.9	**8.18E-08**	3	1.5E-04	1	6.1E-06	24.8	7.22E-02	**6.28E-09**	1	0.21	4.7	6.94E-01	33	1.7E-03	328	1.7E-03	1.0	1.00E+00	4.47E-01
	CDK13	0.91	2.3	1	0.05	19.1	2.16E-01	5	2.4E-04	16	8.2E-05	2.9	3.37E-01	1.19E-01	1	0.02	44.5	1.49E-01	8	3.9E-04	25	1.2E-04	3.3	1.33E-01	**1.13E-02**
	RAF1	0.85	2.5	0	0.02	0.0	1.00E+00	0	0.0E+00	12	4.9E-05	0.0	1.00E+00	8.03E-01	4	0.07	54.1	**1.45E-04**	8	3.7E-04	137	5.0E-04	0.7	1.00E+00	**6.76E-04**
	ZEB2	1.00	3.9	2	0.03	60.8	**8.20E-03**	1	4.5E-05	1	4.1E-06	11.0	8.06E-01	**7.11E-03**	1	0.04	24.8	2.40E-01	9	4.1E-04	69	2.6E-04	1.6	6.55E-01	1.34E-01
	SOS1	1.00	3.0	1	0.06	16.1	2.20E-01	1	4.4E-05	11	4.5E-05	1.0	1.00E+00	4.69E-01	3	0.07	40.9	**2.57E-03**	13	5.8E-04	139	5.1E-04	1.1	1.00E+00	**2.82E-03**
	LZTR1	0.00	0.6	0	0.03	0.0	1.00E+00	18	8.2E-04	225	8.6E-04	1.0	1.00E+00	6.75E-01	3	0.04	70.4	**7.75E-04**	17	7.8E-04	110	4.2E-04	1.8	1.95E-01	**6.68E-05**
▲	NAA15	1.00	3.8	3	0.03	106.9	**7.46E-05**	1	4.4E-05	12	5.2E-05	0.8	1.00E+00	**3.61E-04**	0	0.02	0.0	1.00E+00	2	8.7E-05	10	4.3E-05	2.0	9.33E-01	5.71E-01
	RIT1	0.13	2.1	0	0.01	0.0	1.00E+00	2	8.9E-05	15	6.1E-05	1.4	1.00E+00	6.24E-01	2	0.02	88.1	**7.00E-03**	7	3.1E-04	26	9.6E-05	3.2	1.55E-01	**4.70E-04**
	ANKRD11	1.00	-0.6	2	0.07	28.2	**3.12E-02**	8	3.9E-04	7	3.0E-05	13.2	**1.93E-04**	**4.66E-06**	0	0.02	0.0	1.00E+00	0	0.0E+00	15	6.4E-05	0.0	1.00E+00	7.48E-01
△	CTNNB1	1.00	3.8	3	0.03	106.9	**7.46E-05**	0	0.0E+00	1	4.1E-06	0.0	1.00E+00	**5.20E-04**	0	0.03	0.0	1.00E+00	5	2.2E-04	50	1.8E-04	1.2	1.00E+00	6.12E-01
△	KLF2	0.56	2.2	1	0.01	105.4	9.36E-02	2	1.2E-04	5	4.6E-05	2.7	8.76E-01	1.09E-01	1	0.00	313.7	**4.15E-02**	0	0.0E+00	2	1.9E-05	0.0	1.00E+00	1.13E-01
▲	POGZ	1.00	3.5	2	0.06	35.4	**2.12E-02**	2	8.9E-05	6	2.5E-05	3.6	8.00E-01	**2.08E-02**	1	0.02	46.8	1.49E-01	4	1.8E-04	51	1.9E-04	0.9	1.00E+00	2.08E-01
▲	SMARCC1	1.00	2.4	0	0.05	0.0	1.00E+00	12	5.5E-04	15	6.4E-05	8.6	**2.70E-05**	**9.72E-05**	0	0.02	0.0	1.00E+00	4	1.8E-04	22	9.3E-05	1.9	7.21E-01	5.34E-01
★	CACNA1A	1.00	5.8	1	0.10	10.5	3.10E-01	3	1.4E-04	32	1.5E-04	0.9	1.00E+00	5.07E-01	3	0.56	5.4	1.49E-01	80	3.8E-03	648	2.8E-03	1.3	1.55E-01	**1.85E-02**

★ Novel △ Novel in human ▲ 1st statistical support □ PCGC Previously Established ■ FDR < 0.05

Table shows 60 significant genes from the LOF (left) or D-Mis (right) meta-analysis of the burden of *de novo* mutations in CHD trios and case-control comparison of the frequency of damaging transmitted and unphased variants in 11,555 CHD probands compared to gnomAD controls. FDRs are calculating using the BH method for the DNMs and Case-Control results and the JL-FDR method for the Meta-Analysis results. Blue shading denotes a FDR < 0.05. Genes are sorted based on the Damaging FDR (not shown). All other abbreviations are as previously defined.

Table S8 [Continued] | Independent contribution of *de novo* and transmitted LOF and D-Mis mutations to the sixty significantly enriched genes

B

	Annotation			Loss of Function											D-Mis										
	Gene	Intolerance		Poisson of DNMs in Trios				Case-Control of Ultra-Rare TUVs						Meta-Analysis	Poisson of DNMs in Trios				Case-Control of Ultra-Rare TUVs						Meta-Analysis
								CHD		gnomAD		Fisher's Exact							CHD		gnomAD		Fisher's Exact		
		pLI	MisZ	Obs	Exp	Enrich	FDR	Obs	AF	Obs	AF	Enrich	FDR	FDR	Obs	Exp	Enrich	FDR	Obs	AF	Obs	AF	Enrich	FDR	FDR
★	NR6A1	0.98	1.8	1	0.02	52.9	1.36E-01	1	4.5E-05	4	1.8E-05	2.5	1.00E+00	2.28E-01	2	0.09	21.8	**4.68E-02**	14	6.3E-04	194	7.7E-04	0.8	1.00E+00	9.83E-02
★	AHNAK	0.90	-3.0	1	0.06	16.5	2.20E-01	10	4.4E-04	84	3.2E-04	1.4	8.44E-01	3.22E-01	1	0.00	382.8	**4.04E-02**	2	8.8E-05	3	1.2E-05	7.1	4.34E-01	**8.81E-03**
★	PCBP3	0.84	2.0	1	0.02	56.4	1.35E-01	4	2.2E-04	8	3.5E-05	6.2	1.17E-01	**1.68E-02**	0	0.01	0.0	1.00E+00	2	1.1E-04	10	4.3E-05	2.5	8.18E-01	5.58E-01
□	SMAD6	0.00	-0.6	1	0.02	61.5	1.29E-01	29	1.6E-03	58	4.0E-04	4.1	**7.09E-07**	**1.67E-07**	0	0.11	0.0	1.00E+00	29	1.6E-03	271	1.7E-03	1.0	1.00E+00	6.43E-01
▲	GANAB	1.00	2.2	1	0.05	21.1	2.05E-01	2	8.9E-05	24	9.8E-05	0.9	1.00E+00	4.13E-01	1	0.15	6.9	5.58E-01	37	1.7E-03	291	1.1E-03	1.5	1.55E-01	6.87E-02
▲	PPP1R12A	1.00	2.6	1	0.04	22.8	2.02E-01	2	9.0E-05	7	3.2E-05	2.9	8.44E-01	2.81E-01	1	0.02	52.3	1.49E-01	2	9.0E-05	13	5.9E-05	1.5	1.00E+00	1.63E-01
▲	RPL5	1.00	1.9	2	0.02	123.7	**2.29E-03**	1	4.6E-05	0	0.0E+00	Inf	4.88E-01	**9.88E-04**	0	0.02	0.0	1.00E+00	1	4.6E-05	26	9.6E-05	0.5	1.00E+00	6.94E-01
★	CLUH	0.99	1.8	0	0.04	0.0	1.00E+00	0	0.0E+00	31	1.4E-04	0.0	1.00E+00	8.03E-01	3	0.21	14.0	**2.59E-02**	42	1.9E-03	393	1.6E-03	1.2	6.55E-01	**1.35E-02**
	CHD4	1.00	6.3	0	0.08	0.0	1.00E+00	3	1.3E-04	23	8.6E-05	1.6	1.00E+00	6.03E-01	4	0.34	11.7	**1.07E-02**	31	1.4E-03	421	1.6E-03	0.9	1.00E+00	**2.73E-02**
▲	TBX18	1.00	0.1	1	0.02	50.2	1.40E-01	3	1.7E-04	5	2.2E-05	7.6	1.84E-01	**3.00E-02**	0	0.07	0.0	1.00E+00	19	1.0E-03	154	6.0E-04	1.7	2.03E-01	3.37E-01
★	U2SURP	1.00	4.1	1	0.05	21.8	2.02E-01	1	4.5E-05	3	1.3E-05	3.3	1.00E+00	3.30E-01	1	0.02	45.3	1.49E-01	2	8.9E-05	19	8.3E-05	1.1	1.00E+00	2.00E-01
★	LHX2	0.99	2.3	2	0.01	221.8	**7.71E-04**	0	0.0E+00	2	8.2E-05	0.0	1.00E+00	**2.16E-03**	0	0.06	0.0	1.00E+00	5	2.4E-04	51	2.0E-04	1.2	1.00E+00	6.14E-01
▲	PTEN	0.26	3.5	2	0.02	98.9	**3.34E-03**	2	8.9E-05	16	6.6E-05	1.3	1.00E+00	**4.75E-03**	0	0.06	0.0	1.00E+00	5	2.2E-04	50	1.9E-04	1.2	1.00E+00	6.10E-01
★	RABGAP1L	0.00	1.1	1	0.05	21.9	2.02E-01	6	2.8E-04	46	1.9E-04	1.4	9.48E-01	3.14E-01	1	0.02	50.8	1.49E-01	1	4.7E-05	38	1.6E-04	0.3	1.00E+00	2.40E-01
	DDX3X	1.00	4.3	0	0.02	0.0	1.00E+00	2	1.2E-04	1	6.2E-06	19.8	2.22E-01	3.74E-01	1	0.02	57.7	1.47E-01	0	0.0E+00	0	0.0E+00	0.0	1.00E+00	2.24E-01
△	HDAC7	1.00	2.8	1	0.03	32.6	1.74E-01	6	2.9E-04	22	1.0E-04	2.9	2.49E-01	6.64E-02	0	0.04	0.0	1.00E+00	5	2.4E-04	33	1.4E-04	1.8	7.21E-01	5.30E-01
	BRAF	1.00	3.7	0	0.04	0.0	1.00E+00	0	0.0E+00	8	3.4E-05	0.0	1.00E+00	8.03E-01	2	0.05	42.3	**2.23E-02**	6	2.7E-04	47	2.0E-04	1.4	9.33E-01	**2.37E-02**
▲	ZIC3	0.92	2.5	0	0.01	0.0	1.00E+00	5	3.5E-04	6	3.9E-05	9.1	**2.27E-02**	7.45E-02	0	0.02	0.0	1.00E+00	4	2.8E-04	14	9.1E-05	3.1	4.34E-01	4.34E-01
★	KRT13	0.00	-0.5	0	0.01	0.0	1.00E+00	3	1.3E-04	25	1.0E-04	1.3	1.00E+00	6.27E-01	2	0.07	30.0	**3.51E-02**	18	8.0E-04	235	8.9E-04	0.9	1.00E+00	5.48E-02
	NKX2-5	0.95	0.2	0	0.01	0.0	1.00E+00	11	5.5E-04	25	1.1E-04	4.9	**3.01E-03**	**9.88E-03**	0	0.07	0.0	1.00E+00	13	6.5E-04	98	4.1E-04	1.6	5.36E-01	4.76E-01
▲	ITSN2	0.00	1.1	0	0.08	0.0	1.00E+00	6	2.6E-04	127	5.3E-04	0.5	1.00E+00	7.38E-01	2	0.06	36.3	**2.59E-02**	21	9.2E-04	135	5.1E-04	1.8	1.55E-01	**1.81E-03**
	TBX1	0.84	0.7	1	0.02	65.3	1.26E-01	4	2.3E-04	35	1.8E-04	1.3	1.00E+00	1.76E-01	1	0.07	14.2	3.60E-01	10	5.8E-04	127	6.5E-04	0.9	1.00E+00	3.44E-01
★	SVEP1	1.00	1.9	1	0.10	10.0	3.20E-01	9	4.0E-04	61	2.6E-04	1.5	8.06E-01	3.53E-01	0	0.21	0.0	1.00E+00	64	2.8E-03	518	2.0E-03	1.4	1.55E-01	2.55E-01
★	NGFR	0.10	1.4	0	0.01	0.0	1.00E+00	1	4.9E-05	10	4.4E-05	1.1	1.00E+00	6.77E-01	2	0.08	25.5	**4.04E-02**	11	5.4E-04	139	5.5E-04	1.0	1.00E+00	6.14E-02
▲	TSC1	1.00	2.3	1	0.03	28.6	1.85E-01	0	0.0E+00	9	3.7E-05	0.0	1.00E+00	4.01E-01	1	0.14	7.3	5.38E-01	36	1.6E-03	353	1.3E-03	1.2	6.55E-01	2.32E-01
★	CUL3	1.00	4.8	1	0.03	36.8	1.61E-01	2	9.0E-05	11	4.7E-05	1.9	1.00E+00	2.73E-01	0	0.06	0.0	1.00E+00	9	4.0E-04	49	1.9E-04	2.1	3.35E-01	4.09E-01
▲	KDR	1.00	1.0	0	0.05	0.0	1.00E+00	10	5.4E-04	26	1.1E-04	5.1	**3.54E-03**	**1.30E-02**	0	0.08	0.0	1.00E+00	21	1.1E-03	210	7.7E-04	1.5	4.66E-01	4.56E-01
▲	FOXC2	0.13	-0.3	1	0.01	87.5	1.05E-01	0	0.0E+00	6	3.6E-05	0.0	1.00E+00	2.64E-01	1	0.10	10.5	4.14E-01	17	8.9E-04	187	1.0E-03	0.9	1.00E+00	3.89E-01
★	PPL	0.00	-3.7	1	0.06	17.7	2.17E-01	17	7.9E-04	230	8.6E-04	0.9	1.00E+00	4.59E-01	1	0.05	20.9	2.70E-01	15	7.0E-04	245	9.2E-04	0.8	1.00E+00	3.25E-01
▲	EP300	1.00	2.0	2	0.09	22.2	**4.32E-02**	2	9.1E-05	13	5.3E-05	1.7	1.00E+00	9.08E-02	1	0.27	3.7	8.28E-01	38	1.7E-03	547	2.0E-03	0.9	1.00E+00	5.10E-01

★ Novel △ Novel in human ▲ 1st statistical support □ PCGC Previously Established ■ FDR < 0.05

Table S9 | TDT Results for damaging variants in MIPseq panel genes from 3,887 CHD WES Trios

A

Variant Class	MIPseq Panel Genes (n=248)							
	Parental Transmission				Chi-Squared Test			Probands Explained
	Trans	Non-Trans	Total	% T	Enrich	χ^2	P-Value	
D-Mis	1,136	1,021	2,157	53%	1.1	6.1	1.33E-02	3.0%
LOF	244	132	376	65%	1.8	33.4	7.65E-09	2.9%
Damaging	1,380	1,153	2,533	54%	1.2	20.3	6.47E-06	5.8%

B

Variant Class	Significant MIPseq Panel Genes (n=60)							
	Parental Transmission				Chi-Squared Test			Probands Explained
	Trans	Non-Trans	Total	% T	Enrich	χ^2	P-Value	
D-Mis	426	300	726	59%	1.4	21.9	2.92E-06	3.2%
LOF	118	34	152	78%	3.5	46.4	9.54E-12	2.2%
Damaging	544	334	878	62%	1.6	50.2	1.37E-12	5.4%

C

Variant Class	Non-Significant MIPseq Panel Genes (n=188)							
	Parental Transmission				Chi-Squared Test			Probands Explained
	Trans	Non-Trans	Total	% T	Enrich	χ^2	P-Value	
D-Mis	710	721	1,431	50%	1.0	0.1	7.71E-01	N.S.
LOF	126	98	224	56%	1.3	3.5	6.14E-02	N.S.
Damaging	836	819	1,655	51%	1.0	0.2	6.76E-01	N.S.

D

Variant Class	Significant MIPseq Panel Genes w/o MYH6 & NOTCH1 (n=58)							
	Parental Transmission				Chi-Squared Test			Probands Explained
	Trans	Non-Trans	Total	% T	Enrich	χ^2	P-Value	
D-Mis	322	255	577	56%	1.3	7.8	5.28E-03	1.7%
LOF	101	30	131	77%	3.4	38.5	5.53E-10	1.8%
Damaging	423	285	708	60%	1.5	26.9	2.14E-07	3.6%

E

Gene			D-Mis								Loss of Function								Protein-Damaging							
			Parental Transmission				Chi-Squared Test			Probands Explained	Parental Transmission				Chi-Squared Test			Probands Explained	Parental Transmission				Chi-Squared Test			Probands Explained
Name	pLI	Mis Z	Trans	Non-Trans	Total	% T	Enrich	χ^2	P-Value		Trans	Non-Trans	Total	% T	Enrich	χ^2	P-Value		Trans	Non-Trans	Total	% T	Enrich	χ^2	P-Value	
MYH6	0.0	0.9	68	29	97	70%	2.3	15.7	7.50E-05	1.0%	6	4	10	60%	1.5	0.4	5.27E-01	N.S.	74	33	107	69%	2.2	15.7	7.50E-05	1.1%
NOTCH1	1.0	3.4	36	16	52	69%	2.2	7.7	5.54E-03	0.5%	11	0	11	100%	Inf	11.0	9.11E-04	0.3%	47	16	63	75%	2.9	15.3	9.40E-05	0.8%
SMAD6	0.0	-0.6	11	3	14	79%	3.7	4.6	3.25E-02	0.2%	12	3	15	80%	4.0	5.4	2.01E-02	0.2%	23	6	29	79%	3.8	10.0	1.59E-03	0.4%
NODAL	1.0	1.0	3	1	4	75%	3.0	1.0	3.17E-01	N.S.	7	0	7	100%	Inf	7.0	8.15E-03	0.2%	10	1	11	91%	10.0	7.4	6.66E-03	0.2%
SMARCC1	1.0	2.4	2	1	3	67%	2.0	0.3	5.64E-01	N.S.	6	0	6	100%	Inf	6.0	1.43E-02	0.2%	8	1	9	89%	8.0	5.4	1.96E-02	0.2%
SVEP1	1.0	1.9	22	11	33	67%	2.0	3.7	5.55E-02	N.S.	5	1	6	83%	5.0	2.7	1.02E-01	N.S.	27	12	39	69%	2.3	5.8	1.63E-02	0.4%
FLT4	1.0	2.8	11	5	16	69%	2.2	2.3	1.34E-01	N.S.	8	3	11	73%	2.7	2.3	1.32E-01	N.S.	19	8	27	70%	2.4	4.5	3.43E-02	0.3%
ZIC3	0.9	2.5	1	0	1	100%	Inf	1.0	3.17E-01	N.S.	3	0	3	100%	Inf	3.0	8.33E-02	N.S.	4	0	4	100%	Inf	4.0	4.55E-02	0.1%
JAG1	1.0	3.2	12	9	21	57%	1.3	0.4	5.13E-01	N.S.	4	0	4	100%	Inf	4.0	4.55E-02	0.1%	16	9	25	64%	1.8	2.0	1.62E-01	N.S.

Tables contain summary TDT results for **[A]** MIPseq panel genes (n=248), **[B]** Significant MIPseq Panel Genes (n=60), **[C]** Significant MIPseq Panel Genes after removal of *MYH6* and *NOTCH1* (n=58), and [**D**] Non-Significant MIPseq Panel Genes (n=188). In **[E]**, TDT results for individual genes that had over-transmission of D-mis, LOF, or Damaging variants are shown. Abbreviations are: 'Trans' is # of transmitted variants from parents to proband, 'Non-Trans' is # of variants not transmitted, "% T" is the percent of variants that were transmitted, 'enrich' is enrichment, and p-value is from the chi-square test. Individual genes are sorted by increasing p-value using minimum p-value from any shown test. P-values < 0.05 are colored in red.

Table S10 | Proportion of CHD trios explained by mutations in MIPseq panel genes

A

Functional Class	DNMs in Significantly Enriched Panel Genes (n = 60)						
	Obs	Freq	Exp	Freq	Enrich	P-Val	% Explained
Synonymous	8	2.1E-03	8.5	2.2E-03	0.95	6.74E-01	N.S.
Tolerated Mis	21	5.4E-03	11.8	3.0E-03	1.78	6.10E-06	0.24%
Damaging Mis	91	2.3E-02	6.5	1.7E-03	13.94	7.83E-59	2.17%
Loss of Function	118	3.0E-02	2.4	6.2E-04	48.65	4.96E-151	2.97%
Protein-Damaging	209	5.4E-02	8.9	2.3E-03	23.42	1.28E-201	5.15%

B

Functional Class	DNMs after Exclusion of Significantly Enriched Panel Genes (n = 188)						
	Obs	Freq	Exp	Freq	Enrich	P-Val	% Explained
Synonymous	22	5.7E-03	23.7	6.1E-03	0.93	6.66E-01	N.S.
Tolerated Mis	65	1.7E-02	39.5	1.0E-02	1.65	1.23E-04	0.66%
Damaging Mis	43	1.1E-02	15.4	4.0E-03	2.80	5.72E-09	0.71%
Loss of Function	36	9.3E-03	8.4	2.2E-03	4.30	1.30E-12	0.71%
Protein-Damaging	79	2.0E-02	23.8	6.1E-03	3.32	3.82E-19	1.42%

C

Functional Class	TDT in Significant Enriched Panel Genes (n=60)							% Explained
	Transmitted	Non-Transmitted	Total	% Trans	Enrich	χ^2	P-Value	
Damaging Mis	427	301	728	58.7%	1.4	22	3.0E-06	3.2%
Loss of Function	118	34	152	77.6%	3.5	46	9.5E-12	2.2%
Protein-Damaging	545	335	880	61.9%	1.6	50	1.5E-12	5.4%

D

Functional Class	TDT after Exclusion of Significantly Enriched Panel Genes (n = 188)							% Explained
	Transmitted	Non-Transmitted	Total	% Trans	Enrich	χ^2	P-Value	
Damaging Mis	710	721	1,431	49.6%	1.0	0.1	7.7E-01	N.S.
Loss of Function	126	98	224	56.3%	1.3	3.5	6.1E-02	N.S.
Protein-Damaging	836	819	1,655	50.5%	1.0	0.2	6.8E-01	N.S.

Panels [A] and **[B]** show the Poisson P-values vs. expectation and observed % excess of DNMs in indicated mutation type and gene sets. Panels **[C]** and **[D]** show Chi-square P-values and % of CHD probands explained by transmission of damaging mutations in indicated gene sets. Abbreviations are as previously described. P-values < 0.05 are colored in red.

Table S11 | Fraction of CHD cardiac and extracardiac phenotypes explained by damaging DNMs and transmitted variants

Cohort		Poisson of Damaging DNMs							TDT of Damaging Parental Variants							
		Cases		Mutability		Poisson Test			Parental Transmission				Chi-Squared Test			
Phenotype	# Sig Genes in *Any* Phenotype	Obs	Rate	Exp	Rate	Enrich	P-Val	Probands Explained	Trans	Non-Trans	Total	% T	Enrich	χ^2	P-Value	Probands Explained
All	60	209	5.4E-02	8.9	2.3E-03	23.4	**1.30E-201**	5.1%	546	335	881	62.0%	1.6	50.5	**1.2E-12**	5.4%
CTD	60	56	8.9E-02	1.4	2.3E-03	38.7	**3.50E-67**	8.6%	87	50	137	63.5%	1.7	10.0	**1.6E-03**	5.9%
TOF	60	29	3.8E-02	1.7	2.3E-03	16.8	**1.70E-25**	3.6%	114	57	171	66.7%	2.0	19.0	**1.3E-05**	7.6%
LAT	60	15	2.3E-02	1.5	2.3E-03	10.1	**7.10E-11**	2.1%	79	61	140	56.4%	1.3	2.3	1.3E-01	N.S.
LVO	60	23	4.6E-02	1.2	2.3E-03	19.9	**3.70E-22**	4.3%	74	43	117	63.2%	1.7	8.2	**4.2E-03**	6.2%
HLHS	60	42	7.5E-02	1.3	2.3E-03	32.7	**7.80E-48**	7.3%	82	44	126	65.1%	1.9	11.5	**7.1E-04**	6.8%
ASD	60	14	6.5E-02	0.5	2.3E-03	28.2	**3.90E-16**	6.3%	35	18	53	66.0%	1.9	5.5	**2.0E-02**	7.9%
AVC	60	8	5.0E-02	0.4	2.3E-03	21.8	**5.90E-09**	4.8%	24	22	46	52.2%	1.1	0.1	7.7E-01	N.S.
OTH	60	21	5.2E-02	0.9	2.3E-03	22.6	**1.70E-21**	5.0%	50	38	88	56.8%	1.3	1.6	2.0E-01	N.S.
Isolated	60	33	2.7E-02	2.8	2.3E-03	11.6	**6.90E-24**	2.4%	179	97	276	64.9%	1.8	24.4	**8.0E-07**	6.6%
EC	60	75	7.4E-02	2.3	2.3E-03	32.2	**1.40E-83**	7.2%	137	96	233	58.8%	1.4	7.2	**7.2E-03**	4.0%
NDD	60	16	6.9E-02	0.5	2.3E-03	30	**1.20E-18**	6.7%	26	18	44	59.1%	1.4	1.5	2.3E-01	N.S.
NDD & EC	60	43	1.6E-01	0.6	2.3E-03	71.5	**3.00E-63**	16.2%	28	27	55	50.9%	1.0	0.0	8.9E-01	N.S.

Percent of probands with different CHD and extracardiac phenotypes that can be explained by Damaging DNMs and transmitted variants in the 60 significantly enriched genes is shown. 'Trans' is number of transmitted variants from parents to probands. 'Non-Trans' is the number of non-transmitted variants identified in parents. '% T' is percent of variants transmitted. Other abbreviations are as previously described. P-values < 0.05 are colored in red.

Table S12 | Burden of DNMs stratified by patient extracardiac phenotype and gene set

Phenotype of CHD Trios	Func	Exome-wide Genes (n ≈ 19,000)							Significant Panel Genes (n = 60)							Significant Chromatin Panel Genes (n = 10)						
		Obs	Freq	Exp	Freq	Enrich	P-Val	% Explained	Obs	Freq	Exp	Freq	Enrich	P-Val	% Explained	Obs	Freq	Exp	Freq	Enrich	P-Val	% Explained
All Probands (n = 3,887)	Syn	1,064	2.7E-01	1,219.1	3.1E-01	0.9	1.0E+00	NS	8	2.1E-03	8.4	2.2E-03	1.0	6.0E-01	NS	1	2.6E-04	1.9	4.8E-04	0.5	8.4E-01	NS
	T-Mis	2,155	5.5E-01	2,232.6	5.7E-01	1.0	9.5E-01	NS	21	5.4E-03	11.7	3.0E-03	1.8	**8.7E-03**	0.2%	5	1.3E-03	2.8	7.2E-04	1.8	1.5E-01	NS
	D-Mis	650	1.7E-01	480.5	1.2E-01	1.4	**1.2E-13**	4.4%	91	2.3E-02	6.5	1.7E-03	14.0	**1.1E-69**	2.2%	19	4.9E-03	1.8	4.8E-04	10.3	**1.7E-13**	0.4%
	LOF	558	1.4E-01	374.0	9.6E-02	1.5	**4.6E-19**	4.7%	118	3.0E-02	2.4	6.2E-04	48.6	**5.0E-151**	3.0%	53	1.4E-02	0.8	2.0E-04	69.1	**8.5E-77**	1.3%
	Dmg	1,208	3.1E-01	854.6	2.2E-01	1.4	**3.1E-30**	9.1%	209	5.4E-02	8.9	2.3E-03	23.4	**1.3E-201**	5.1%	72	1.9E-02	2.6	6.7E-04	27.5	**1.4E-75**	1.8%
Isolated (n = 1,238)	Syn	352	2.8E-01	388.3	3.1E-01	0.9	9.7E-01	NS	1	8.1E-04	2.7	2.2E-03	0.4	9.3E-01	NS	0	0.0E+00	0.6	4.8E-04	0.0	1.0E+00	NS
	T-Mis	710	5.7E-01	711.1	5.7E-01	1.0	5.2E-01	NS	7	5.7E-03	3.7	3.0E-03	1.9	8.3E-02	NS	1	8.1E-04	0.9	7.2E-04	1.1	5.9E-01	NS
	D-Mis	184	1.5E-01	153.0	1.2E-01	1.2	**8.2E-03**	2.5%	16	1.3E-02	2.1	1.7E-03	7.7	**7.8E-10**	1.1%	2	1.6E-03	0.6	4.8E-04	3.4	1.2E-01	NS
	LOF	150	1.2E-01	119.1	9.6E-02	1.3	**3.6E-03**	2.5%	17	1.4E-02	0.8	6.2E-04	22.0	**1.7E-17**	1.3%	0	0.0E+00	0.2	2.0E-04	0.0	1.0E+00	NS
	Dmg	334	2.7E-01	272.2	2.2E-01	1.2	**1.6E-04**	5.0%	33	2.7E-02	2.8	2.3E-03	11.6	**6.9E-24**	2.4%	2	1.6E-03	0.8	6.7E-04	2.4	2.0E-01	NS
EC (n = 1,014)	Syn	280	2.8E-01	318.0	3.1E-01	0.9	9.9E-01	NS	1	9.9E-04	2.2	2.2E-03	0.5	8.9E-01	NS	0	0.0E+00	0.5	4.8E-04	0.0	1.0E+00	NS
	T-Mis	590	5.8E-01	582.4	5.7E-01	1.0	3.8E-01	NS	7	6.9E-03	3.0	3.0E-03	2.3	**3.6E-02**	0.4%	1	9.9E-04	0.7	7.2E-04	1.4	5.2E-01	NS
	D-Mis	172	1.7E-01	125.4	1.2E-01	1.4	**4.5E-05**	4.6%	30	3.0E-02	1.7	1.7E-03	17.7	**5.5E-27**	2.8%	9	8.9E-03	0.5	4.8E-04	18.7	**2.5E-09**	0.8%
	LOF	157	1.5E-01	97.6	9.6E-02	1.6	**2.0E-08**	5.9%	45	4.4E-02	0.6	6.2E-04	71.1	**5.1E-66**	4.4%	28	2.8E-02	0.2	2.0E-04	140.0	**7.3E-50**	2.7%
	Dmg	329	3.2E-01	222.9	2.2E-01	1.5	**1.9E-11**	10.5%	75	7.4E-02	2.3	2.3E-03	32.2	**1.4E-83**	7.2%	37	3.6E-02	0.7	6.7E-04	54.3	**2.6E-50**	3.6%
NDD (n = 232)	Syn	60	2.6E-01	72.8	3.1E-01	0.8	9.4E-01	NS	3	1.3E-02	0.5	2.2E-03	6.0	**1.4E-02**	1.1%	0	0.0E+00	0.1	4.8E-04	0.0	1.0E+00	NS
	T-Mis	113	4.9E-01	133.3	5.7E-01	0.8	9.7E-01	NS	4	1.7E-02	0.7	3.0E-03	5.7	**5.6E-03**	1.4%	3	1.3E-02	0.2	7.2E-04	17.9	**6.9E-04**	1.2%
	D-Mis	42	1.8E-01	28.7	1.2E-01	1.5	**1.2E-02**	5.7%	4	1.7E-02	0.4	1.7E-03	10.3	**6.9E-04**	1.6%	0	0.0E+00	0.1	4.8E-04	0.0	1.0E+00	NS
	LOF	44	1.9E-01	22.3	9.6E-02	2.0	**3.3E-05**	9.3%	12	5.2E-02	0.1	6.2E-04	82.9	**1.5E-19**	5.1%	4	1.7E-02	0.0	2.0E-04	87.4	**1.8E-07**	1.7%
	Dmg	86	3.7E-01	51.0	2.2E-01	1.7	**4.9E-06**	15.1%	16	6.9E-02	0.5	2.3E-03	30.0	**1.2E-18**	6.7%	4	1.7E-02	0.2	6.7E-04	25.6	**2.2E-05**	1.7%
NDD & EC (n = 262)	Syn	59	2.3E-01	82.2	3.1E-01	0.7	1.0E+00	NS	0	0.0E+00	0.6	2.2E-03	0.0	1.0E+00	NS	0	0.0E+00	0.1	4.8E-04	0.0	1.0E+00	NS
	T-Mis	144	5.5E-01	150.5	5.7E-01	1.0	7.1E-01	NS	0	0.0E+00	0.8	3.0E-03	0.0	1.0E+00	NS	0	0.0E+00	0.2	7.2E-04	0.0	1.0E+00	NS
	D-Mis	68	2.6E-01	32.4	1.2E-01	2.1	**3.3E-08**	13.6%	23	8.8E-02	0.4	1.7E-03	52.5	**1.4E-31**	8.6%	6	2.3E-02	0.1	4.8E-04	48.2	**4.7E-09**	2.2%
	LOF	66	2.5E-01	25.2	9.6E-02	2.6	**1.1E-11**	15.6%	20	7.6E-02	0.2	6.2E-04	122.3	**6.6E-35**	7.6%	16	6.1E-02	0.1	2.0E-04	309.6	**1.2E-34**	6.1%
	Dmg	134	5.1E-01	57.6	2.2E-01	2.3	**6.6E-18**	29.2%	43	1.6E-01	0.6	2.3E-03	71.5	**3.0E-63**	16.2%	22	8.4E-02	0.2	6.7E-04	124.9	**1.9E-38**	8.3%

Table shows burden of DNMs in CHD proband trios sorted by presence or absence of extracardiac abnormalities using all exome-wide genes, 60 significant genes in the MIPseq panel, or significant chromatin genes in the MIPseq panel. Abbreviations are as follows: 'Func' is the predicted functional effect on the protein as described previously; 'Obs' is the number of observed DNMs; 'Exp' is the expected number of DNMs based on gene mutability; 'Freq' is the expected DNMs per tested proband trio; 'Enrich' is the enrichment; 'P-Val' is the Poisson p-value; and '% Explained' is the percent of probands with disease attributable to the observed mutations. P-values < 0.05 are colored red.

Table S13 | Meta-analysis of DNMs and TUVs reveals residual pathogenic signal of Damaging variants in chromatin modifier genes

A

Chromatin Genes in MIPseq Panel	*De Novo* Mutations: CHD Probands		Expected		Poisson Test		Transmitted & Unphased Variants: CHD Probands		gnomAD Controls		Fisher's Exact Test		Meta-Analysis	% Cases Attributable to DNMs
	Obs	Rate	Exp	Rate	Enrich	P-Val	Obs	Rate	Obs	Rate	Enrich	P-Val	P-Val	
Synonymous	18	4.63E-03	21.1	5.43E-03	0.85	7.79E-01	6,160	2.68E-01	70,993	2.62E-01	1.02	2.49E-02	9.61E-02	NS
Tolerated Mis	62	1.60E-02	36.6	9.40E-03	1.70	7.86E-05	7,491	3.25E-01	95,242	3.51E-01	0.93	1.00E+00	8.21E-04	0.65%
Damaging Mis	38	9.78E-03	12.9	3.32E-03	2.95	1.13E-08	1,769	7.68E-02	20,940	7.72E-02	1.00	5.74E-01	1.28E-07	0.65%
Loss of Function	84	2.16E-02	7.6	1.96E-03	11.02	2.00E-56	298	1.29E-02	3,129	1.15E-02	1.12	3.14E-02	8.34E-56	1.96%
Protein-Damaging	122	3.14E-02	20.5	5.28E-03	5.95	1.81E-52	2,067	8.98E-02	24,069	8.87E-02	1.01	2.92E-01	6.39E-51	2.61%

B

Gene Annotation		Damaging *De Novo* Mutations: CHD Probands			Poisson Test				Damaging Ultra-Rare Transmitted & Unphased Variants: CHD Probands				gnomAD Controls				Fisher's Exact Exact			Meta-Analysis	
Name	pLI	# LOF	# D-mis	# Dmg	Exp	Enrich	P-Val	FDR	# LOF	# D-mis	# Dmg	Freq	# LOF	# D-mis	# Dmg	Freq	Enrich	P-Val	BH-FDR	P-Val	FDR
KMT2D	1.00	21	6	27	0.61	44.5	7.21E-35	1.79E-32	12	96	108	5.0E-03	35	796	831	3.3E-03	1.5	7.43E-05	2.30E-03	4.54E-38	1.09E-32
CHD7	1.00	16	4	20	0.37	54.7	5.22E-28	6.47E-26	18	53	71	3.1E-03	9	594	603	2.3E-03	1.3	1.44E-02	1.19E-01	5.45E-28	1.41E-24
KDM5B	0.00	4	1	5	0.22	23.2	3.23E-06	8.90E-05	16	24	40	1.8E-03	84	325	409	1.5E-03	1.1	2.27E-01	6.81E-01	3.76E-06	2.47E-05
NSD1	1.00	4	2	6	0.37	16.2	2.57E-06	8.90E-05	0	56	56	2.5E-03	16	614	630	2.3E-03	1.1	3.02E-01	8.09E-01	4.12E-06	3.82E-05
CTNNB1	1.00	3	0	3	0.06	47.8	3.94E-05	5.48E-04	0	5	5	2.2E-04	1	50	51	1.9E-04	1.2	4.46E-01	9.44E-01	7.84E-05	5.60E-04
POGZ	1.00	2	1	3	0.08	38.5	7.43E-05	8.77E-04	2	4	6	2.7E-04	6	51	57	2.1E-04	1.2	3.67E-01	8.84E-01	1.16E-04	8.33E-04
SMARCC1	1.00	0	0	0	0.07	0.0	1.00E+00	1.00E+00	12	4	16	7.3E-04	15	22	37	1.6E-04	4.6	5.40E-06	2.23E-04	2.33E-04	1.08E-03
CHD4	1.00	0	4	4	0.42	9.6	9.10E-04	8.06E-03	3	31	34	1.5E-03	23	421	444	1.7E-03	0.9	7.26E-01	1.00E+00	2.30E-03	8.77E-03
HDAC7	1.00	1	0	1	0.07	13.8	7.01E-02	2.12E-01	6	5	11	5.3E-04	22	33	55	2.3E-04	2.4	1.32E-02	1.17E-01	4.95E-03	1.96E-02
EP300	1.00	2	1	3	0.36	8.3	5.98E-03	3.30E-02	2	38	40	1.8E-03	13	547	560	2.1E-03	0.9	8.09E-01	1.00E+00	1.45E-02	4.95E-02

C

Individually Significant Chromatin Genes	*De Novo* Mutations: CHD Probands		Expected		Poisson Test		Transmitted & Unphased Variants: CHD Probands		gnomAD Controls		Fisher's Exact Test		Meta-Analysis	% Cases Attributable to DNMs
	Obs	Rate	Exp	Rate	Enrich	P-Val	Obs	Rate	Obs	Rate	Enrich	P-Val	P-Val	
Synonymous	1	2.57E-04	1.9	4.78E-04	0.54	8.50E-01	626	2.73E-02	7,138	2.63E-02	1.04	2.01E-01	4.73E-01	NS
Tolerated Mis	5	1.29E-03	2.8	7.22E-04	1.78	1.52E-01	727	3.16E-02	9,158	3.38E-02	0.94	9.58E-01	4.27E-01	NS
Damaging Mis	19	4.89E-03	1.8	4.75E-04	10.28	1.06E-13	316	1.38E-02	3,453	1.27E-02	1.08	9.71E-02	3.41E-13	0.44%
Loss of Function	53	1.36E-02	0.8	1.97E-04	69.12	7.80E-76	71	3.09E-03	224	8.36E-04	3.70	1.80E-17	2.98E-90	1.34%
Protein-Damaging	72	1.85E-02	2.6	6.73E-04	27.54	9.50E-76	387	1.68E-02	3,677	1.36E-02	1.24	3.65E-05	6.38E-78	1.79%

D

Not Individually Significant Chromatin Genes	*De Novo* Mutations: CHD Probands		Expected		Poisson Test		Transmitted & Unphased Variants: CHD Probands		gnomAD Controls		Fisher's Exact Test		Meta-Analysis	% Cases Attributable to DNMs
	Obs	Rate	Exp	Rate	Enrich	P-Val	Obs	Rate	Obs	Rate	Enrich	P-Val	P-Val	
Synonymous	17	4.37E-03	19.2	4.95E-03	0.88	7.26E-01	5,534	2.40E-01	63,855	2.35E-01	1.02	4.20E-02	1.37E-01	NS
Tolerated Mis	57	1.47E-02	33.7	8.68E-03	1.69	1.63E-04	6,764	2.94E-01	86,084	3.17E-01	0.93	1.00E+00	1.59E-03	0.60%
Damaging Mis	19	4.89E-03	11.1	2.84E-03	1.72	1.84E-02	1,453	6.31E-02	17,487	6.44E-02	0.98	7.88E-01	7.60E-02	0.20%
Loss of Function	31	7.98E-03	6.9	1.76E-03	4.52	1.34E-11	227	9.86E-03	2,905	1.07E-02	0.92	8.93E-01	3.13E-10	0.62%
Protein-Damaging	50	1.29E-02	17.9	4.61E-03	2.79	3.76E-10	1,680	7.30E-02	20,392	7.51E-02	0.97	8.88E-01	7.61E-09	0.83%

$P_{GWS} < 2 \times 10^{-6}$ FDR < 0.05

Meta-analysis combining results of analysis of DNMs in 3,887 CHD proband trios, and of transmitted or unphased variants in 11,555 CHD probands for chromatin genes sets: **[A]** Results for all 165 chromatin genes in the 248 gene panel; **[C]** 10 chromatin panel genes that are significantly enriched in meta-analysis; **[D]** 155 chromatin panel genes after removal of genes that are significantly enriched in the Damaging variant meta-analysis. **[B]** For each of the 10 individually significant chromatin genes, the table shows the Damaging meta-analysis using Poisson test of DNMs and case-control test of TUVs. For all tables, P-values < GWS (2.6×10^{-6}) are shaded in red and FDR < 0.05 in blue. When meta-analysis P-value is < 0.05, the '% cases attributable to DNMs' is calculated by using the observed rate – expected rate. Other abbreviations are as described in prior tables.

Table S14 | Significant enrichment of heterozygous D-Mis *MYH6* variants in CHD probands with LVO, HLHS, ASD, and Isolated CHD

A

CHD Cohort		Case-Control of D-Mis Heterozygous Variants							
		CHD			gnomAD			Fisher's Exact	
Phenotype	Probands	Obs	Total	AF	Obs	Total	AF	Enrich	P-Val
All	11,555	155	20,706	7.49E-03	1,112	270,828	4.11E-03	1.82	**4.57E-11**
CTD	2,442	25	4,376	5.71E-03	1,112	270,828	4.11E-03	1.39	6.89E-02
TOF	1,798	18	3,222	5.59E-03	1,112	270,828	4.11E-03	1.36	1.24E-01
LAT	1,968	17	3,527	4.82E-03	1,112	270,828	4.11E-03	1.17	2.89E-01
LVO	1,493	26	2,675	9.72E-03	1,112	270,828	4.11E-03	2.37	**8.60E-05**
HLHS	1,076	28	1,928	1.45E-02	1,112	270,828	4.11E-03	3.54	**2.58E-08**
ASD	710	20	1,272	1.57E-02	1,112	270,828	4.11E-03	3.83	**6.86E-07**
AVC	463	8	830	9.64E-03	1,112	270,828	4.11E-03	2.35	2.34E-02
OTH	1,463	12	2,622	4.58E-03	1,112	270,828	4.11E-03	1.11	3.94E-01
Isolated	4,537	70	8,130	8.61E-03	1,112	270,828	4.11E-03	2.10	**3.72E-08**
EC	2,066	17	3,702	4.59E-03	1,112	270,828	4.11E-03	1.12	3.57E-01
NDD	529	5	948	5.27E-03	1,112	270,828	4.11E-03	1.28	3.51E-01
NDD&EC	500	3	896	3.35E-03	1,112	270,828	4.11E-03	0.82	7.12E-01

$P_{Bonferroni} < 4.17 \times 10^{-3}$

B

Cohort		Parental Transmission				Chi-Square Test		Genotypic	Probands
Phenotype	# Trios	Trans	Non-Trans	Total	% T	χ^2	P-Value	Risk Ratio	Explained
Total	1,280	36	6	42	85.7%	24.2	**3.70E-06**	6.0	0.8%
LVO	504	16	2	18	88.9%	10.9	**9.60E-04**	8.0	2.8%
HLHS	560	13	3	16	81.3%	6.25	**1.20E-02**	4.3	1.8%
ASD	216	7	1	8	87.5%	4.5	**3.40E-02**	7.0	2.8%

Cohort		Parental Transmission				Chi-Square Test		Genotypic	Probands
Phenotype	# Trios	Trans	Non-Trans	Total	% T	χ^2	P-Value	Risk Ratio	Explained
All	2,191	32	23	55	58.2%	1.47	2.2E-01	1.4	NS
CTD	631	7	4	11	63.6%	0.82	3.7E-01	1.8	NS
LAT	646	6	8	14	42.9%	0.29	5.9E-01	0.8	NS
TOF	754	13	6	19	68.4%	2.58	1.1E-01	2.2	NS
AVC + OTH	160	6	5	11	54.5%	0.09	7.6E-01	1.2	NS

[A] Case-control enrichment of D-Mis *MYH6* variants in LVO, HLHS, ASD and isolated CHD after removal of homozygous genotypes from both cases and controls. Additionally, in cases only, compound heterozygotes were removed and variants in *cis* were counted once. P-Values that surpass Bonferroni correction for the number of cardiac and NDD/EC phenotypes ($0.05/12 = 4.17 \times 10^{-3}$) are highlighted in green. **[B]** Enrichment of D-Mis *MYH6* variants by TDT in LVO, HLHS, and ASD; but not other groups. Abbreviations are: 'Trans' is # of transmitted variants from parents to proband, 'Non-Trans' is # of variants not transmitted, "% T" is the percent of variants that were transmitted, 'enrich' is enrichment, and p-value is from the chi-square test. P-Values less than 0.05 are bolded in red.

Table S15 | LOF and D-Mis mutations in *NOTCH1* associated with multiple CHD subtypes and extracardiac phenotypes

A

Cohort				Poisson Test of LOF DNMs						Case-Control Test of LOF TUVs						Meta-Analysis
				CHD Cases		Expected		Poisson Test		CHD Cases		Controls		Fisher's Exact		
Pheno	Total	Trios	Singletons	#	Freq	#	Freq	Enrich	P-Val	#	Freq	#	Freq	Enrich	P-Val	P-Val
All	11,413	3,875	7,538	7	1.81E-03	0.07	1.77E-05	102.0	1.34E-12	30	1.4E-03	9	4.0E-05	34.3	2.58E-24	2.86E-34
CTD	2,442	631	1,811	1	1.58E-03	0.01	1.77E-05	89.7	1.11E-02	7	1.5E-03	9	4.0E-05	37.8	1.32E-08	3.46E-09
TOF	1,798	754	1,044	0	0.00E+00	0.01	1.77E-05	0.0	1.00E+00	9	2.6E-03	9	4.0E-05	66.1	1.57E-12	4.42E-11
LAT	1,968	646	1,322	0	0.00E+00	0.01	1.77E-05	0.0	1.00E+00	0	0.0E+00	9	4.0E-05	0.0	1.00E+00	1.00E+00
LVO	1,493	504	989	3	5.95E-03	0.01	1.77E-05	337.1	1.17E-07	4	1.4E-03	9	4.0E-05	35.4	1.55E-05	5.10E-11
HLHS	1,076	560	516	3	5.36E-03	0.01	1.77E-05	303.3	1.60E-07	7	3.4E-03	9	4.0E-05	86.0	4.99E-11	3.23E-16
ASD	710	216	494	0	0.00E+00	0.00	1.77E-05	0.0	1.00E+00	0	0.0E+00	9	4.0E-05	0.0	1.00E+00	1.00E+00
AVC	463	160	303	0	0.00E+00	0.00	1.77E-05	0.0	1.00E+00	0	0.0E+00	9	4.0E-05	0.0	1.00E+00	1.00E+00
OTH	1,463	404	1,059	0	0.00E+00	0.01	1.77E-05	0.0	1.00E+00	3	1.1E-03	9	4.0E-05	27.1	3.66E-04	3.26E-03
Isolated	4,617	1,238	3,379	2	1.62E-03	0.02	1.77E-05	91.5	2.36E-04	10	1.1E-03	9	4.0E-05	28.6	3.66E-10	2.68E-12
EC	1,932	1,014	918	0	0.00E+00	0.02	1.77E-05	0.0	1.00E+00	6	1.6E-03	9	4.0E-05	41.0	7.45E-08	1.30E-06
NDD	556	232	324	2	8.62E-03	0.00	1.77E-05	488.1	8.37E-06	2	1.9E-03	9	4.0E-05	47.6	1.16E-03	1.89E-07
NDD&EC	473	262	211	1	3.82E-03	0.00	1.77E-05	216.1	4.62E-03	2	2.2E-03	9	4.0E-05	55.8	8.45E-04	5.25E-05

B

Cohort				Poisson Test of D-Mis DNMs						Case-Control Test of D-Mis TUVs						Meta-Analysis
				CHD Cases		Expected		Poisson Test		CHD Cases		Controls		Fisher's Exact		
Pheno	Total	Trios	Singletons	#	Freq	#	Freq	Enrich	P-Val	#	Freq	#	Freq	Enrich	P-Val	P-Val
All	11,413	3,875	7,538	3	7.74E-04	0.39	1.00E-04	7.7	7.33E-03	113	5.1E-03	885	3.5E-03	1.5	1.55E-04	1.67E-05
CTD	2,442	631	1,811	0	0.00E+00	0.06	1.00E-04	0.0	1.00E+00	32	6.9E-03	885	3.5E-03	2.0	4.31E-04	3.77E-03
TOF	1,798	754	1,044	1	1.33E-03	0.08	1.00E-04	13.3	7.26E-02	36	1.0E-02	885	3.5E-03	3.0	2.26E-08	3.48E-08
LAT	1,968	646	1,322	0	0.00E+00	0.06	1.00E-04	0.0	1.00E+00	15	4.0E-03	885	3.5E-03	1.1	3.42E-01	7.08E-01
LVO	1,493	504	989	1	1.98E-03	0.05	1.00E-04	19.8	4.92E-02	7	2.5E-03	885	3.5E-03	0.7	8.68E-01	1.77E-01
HLHS	1,076	560	516	1	1.79E-03	0.06	1.00E-04	17.9	5.45E-02	4	1.9E-03	885	3.5E-03	0.6	9.27E-01	2.01E-01
ASD	710	216	494	0	0.00E+00	0.02	1.00E-04	0.0	1.00E+00	7	5.2E-03	885	3.5E-03	1.5	2.02E-01	5.25E-01
AVC	463	160	303	0	0.00E+00	0.02	1.00E-04	0.0	1.00E+00	5	5.7E-03	885	3.5E-03	1.6	2.01E-01	5.23E-01
OTH	1,463	404	1,059	0	0.00E+00	0.04	1.00E-04	0.0	1.00E+00	7	2.5E-03	885	3.5E-03	0.7	8.54E-01	9.89E-01
Isolated	4,617	1,238	3,379	0	0.00E+00	0.12	1.00E-04	0.0	1.00E+00	47	5.3E-03	885	3.5E-03	1.5	4.67E-03	2.97E-02
EC	1,932	1,014	918	2	1.97E-03	0.10	1.00E-04	19.7	4.81E-03	19	5.1E-03	885	3.5E-03	1.5	6.76E-02	2.94E-03
NDD	556	232	324	0	0.00E+00	0.02	1.00E-04	0.0	1.00E+00	3	2.8E-03	885	3.5E-03	0.8	7.17E-01	9.56E-01
NDD&EC	473	262	211	1	3.82E-03	0.03	1.00E-04	38.2	2.59E-02	3	3.3E-03	885	3.5E-03	1.0	6.12E-01	8.15E-02

(green) $P_{Bonferroni} < 4.17 \times 10^{-3}$

Meta-Analysis results for *NOTCH1* using 3,887 CHD proband trios and 11,555 CHD probands stratified by cardiac and extracardiac phenotypes for [**A**] LOFs and [**B**] D-mis variants. Results include Poisson test of DNMs, case-control test of TUVs, and meta-analysis of both. P-values that surpass Bonferroni correction for all 12 tested phenotypes ($0.05/12 = 4.17\times10^{-3}$) are shaded in green. Columns and abbreviations are as previously described.

Table S16 | Ultra-rare D-mis TUVs in *NOTCH1* EGF #5 in CHD probands

Proband	CHD	EC	NDD	Pos	Ref	Alt	Genotype	Inheritance	MetaSVM	CADD	Bravo	ΔAA
1-14685	CTD	F	Unk	139417500	A	G	Het	Unphased	D	25.3	0.0E+00	C182R
1-02997	CTD	F	F	139417476	G	A	Het	Unphased	D	30.0	0.0E+00	R190C
1-15176	CTD	F	F	139417437	G	A	Het	Unphased	D	32.0	0.0E+00	R203C
1-03313	TOF	F	Unk	139417476	G	A	Het	Unphased	D	30.0	0.0E+00	R190C
1-02040	TOF	F	F	139417476	G	A	Het	Unphased	D	30.0	0.0E+00	R190C
1-09580	TOF	F	Unk	139417439	T	C	Het	Transmitted	D	24.2	0.0E+00	Y202C
BRZ-3730	TOF	Unk	Unk	139417437	G	A	Het	Transmitted	D	32.0	0.0E+00	R203C
1-00918	TOF	F	F	139417425	G	A	Het	Unphased	D	31.0	0.0E+00	R207C
1-14762	CTD	F	Unk	139417470	C	T	Het	Unphased	D	30.0	7.96E-06	G192R
1-06107	CTD	F	Unk	139417454	T	C	Het	Unphased	D	24.6	0.0E+00	N197S
9504000280-0	LAT	Unk	Unk	139417442	G	A	Het	Transmitted	D	27.2	0.0E+00	S201F
1-01453	OTH	F	Unk	139417466	C	T	Het	Unphased	D	28.6	0.0E+00	G193D

Eight mutations that introduce or replace cysteine residues are highlighted in gray versus white for non-cysteine altering mutations. The column with 'CHD' shows the specific CHD subtype. The 'EC' and 'NDD' columns show the presence or absence of EC and NDD, respectively; where "F" is false, "T" is true, and "Unk" denotes unknown for probands with unknown data or age too young for NDD phenotyping. "MetaSVM" designation D denotes damaging and T is tolerated. Allele frequency in Bravo database is shown. The " ΔAA" shows the amino acid change in NOTCH1.

Table S17 | Meta-Analysis of *CHD7* DNMs and ultra-rare TUVs in 11,555 CHD probands reveals genotype-phenotype specificity

A

Cohort				Poisson Test of LOF DNMs						Case-Control Test of LOF TUVs						Meta-Analysis
				CHD Cases		Expected		Poisson Test		CHD Cases		Controls		Fisher's Exact		
Pheno	Total	Trios	Singletons	#	Freq	#	Freq	Enrich	P-Val	#	Freq	#	Freq	Enrich	P-Val	P-Val
All	11,413	3,875	7,538	16	4.13E-03	0.10	2.71E-05	152.5	9.38E-30	18	7.9E-04	9	3.9E-05	20.5	2.61E-13	2.37E-40
CTD	2,442	631	1,811	8	1.27E-02	0.02	2.70E-05	469.6	1.73E-19	6	1.2E-03	9	3.9E-05	32.3	2.93E-07	3.00E-24
TOF	1,798	754	1,044	4	5.31E-03	0.02	2.70E-05	196.5	7.04E-09	6	1.7E-03	9	3.9E-05	43.9	5.01E-08	1.29E-14
LAT	1,968	646	1,322	0	0.00E+00	0.02	2.70E-05	0.0	1.00E+00	2	5.2E-04	9	3.9E-05	13.4	1.34E-02	7.10E-02
LVO	1,493	504	989	1	1.98E-03	0.01	2.70E-05	73.5	1.35E-02	1	3.4E-04	9	3.9E-05	8.8	1.18E-01	1.19E-02
HLHS	1,076	560	516	1	1.79E-03	0.02	2.70E-05	66.1	1.50E-02	1	4.7E-04	9	3.9E-05	12.2	8.66E-02	9.93E-03
ASD	710	216	494	0	0.00E+00	0.01	2.70E-05	0.0	1.00E+00	0	0.0E+00	9	3.9E-05	0.0	1.00E+00	1.00E+00
AVC	463	160	303	1	6.25E-03	0.00	2.70E-05	231.5	4.31E-03	2	2.2E-03	9	3.9E-05	56.8	8.17E-04	4.77E-05
OTH	1,463	404	1,059	1	2.48E-03	0.01	2.70E-05	91.7	1.08E-02	0	0.0E+00	9	3.9E-05	0.0	1.00E+00	5.97E-02
Isolated	4,617	1,238	3,379	0	0.00E+00	0.03	2.70E-05	0.0	1.00E+00	0	0.0E+00	9	3.9E-05	0.0	1.00E+00	1.00E+00
EC	1,932	1,014	918	11	1.08E-02	0.03	2.70E-05	401.8	1.58E-25	12	3.2E-03	9	3.9E-05	81.8	7.53E-17	1.13E-39
NDD	556	232	324	0	0.00E+00	0.01	2.70E-05	0.0	1.00E+00	1	9.1E-04	9	3.9E-05	23.7	4.58E-02	1.87E-01
NDD&EC	473	262	211	5	1.91E-02	0.01	2.70E-05	706.8	1.47E-13	3	3.2E-03	9	3.9E-05	83.5	1.35E-05	8.30E-17

B

Cohort				Poisson Test of D-Mis DNMs						Case-Control Test of D-Mis TUVs						Meta-Analysis
				CHD Cases		Expected		Poisson Test		CHD Cases		Controls		Fisher's Exact		
Pheno	Total	Trios	Singletons	#	Freq	#	Freq	Enrich	P-Val	#	Freq	#	Freq	Enrich	P-Val	P-Val
All	11,413	3,875	7,538	4	1.03E-03	0.26	6.72E-05	15.4	1.56E-04	53	2.3E-03	594	2.3E-03	1.0	4.83E-01	7.91E-04
CTD	2,442	631	1,811	2	3.17E-03	0.04	6.70E-05	47.3	8.69E-04	16	3.3E-03	594	2.3E-03	1.4	9.81E-02	8.84E-04
TOF	1,798	754	1,044	1	1.33E-03	0.05	6.70E-05	19.8	4.93E-02	4	1.1E-03	594	2.3E-03	0.5	9.61E-01	1.92E-01
LAT	1,968	646	1,322	0	0.00E+00	0.04	6.70E-05	0.0	1.00E+00	9	2.3E-03	594	2.3E-03	1.0	5.33E-01	8.68E-01
LVO	1,493	504	989	0	0.00E+00	0.03	6.70E-05	0.0	1.00E+00	3	1.0E-03	594	2.3E-03	0.4	9.64E-01	9.99E-01
HLHS	1,076	560	516	0	0.00E+00	0.04	6.70E-05	0.0	1.00E+00	8	3.8E-03	594	2.3E-03	1.6	1.21E-01	3.77E-01
ASD	710	216	494	1	4.63E-03	0.01	6.70E-05	69.1	1.44E-02	3	2.1E-03	594	2.3E-03	0.9	6.24E-01	5.13E-02
AVC	463	160	303	0	0.00E+00	0.01	6.70E-05	0.0	1.00E+00	6	6.6E-03	594	2.3E-03	2.9	2.05E-02	1.00E-01
OTH	1,463	404	1,059	0	0.00E+00	0.03	6.70E-05	0.0	1.00E+00	4	1.4E-03	594	2.3E-03	0.6	8.96E-01	9.94E-01
Isolated	4,617	1,238	3,379	1	8.08E-04	0.08	6.70E-05	12.1	7.96E-02	20	2.2E-03	594	2.3E-03	1.0	6.07E-01	1.95E-01
EC	1,932	1,014	918	2	1.97E-03	0.07	6.70E-05	29.4	2.21E-03	11	2.9E-03	594	2.3E-03	1.3	2.67E-01	4.97E-03
NDD	556	232	324	0	0.00E+00	0.02	6.70E-05	0.0	1.00E+00	1	9.1E-04	594	2.3E-03	0.4	9.19E-01	9.97E-01
NDD&EC	473	262	211	1	3.82E-03	0.02	6.70E-05	57.0	1.74E-02	1	1.1E-03	594	2.3E-03	0.5	8.83E-01	7.95E-02

(green) $P_{Bonferroni} < 4.17 \times 10^{-3}$

Meta-Analysis results for *CHD7* using 3,887 CHD proband trios and 11,555 CHD probands stratified by cardiac and extracardiac phenotypes for [**A**] LOFs and [**B**] D-mis variants. Results include Poisson test of DNMs, case-control test of TUVs, and meta-analysis of both. The meta-analysis FDR is calculated using the BH-FDR method. P-values that surpass Bonferroni correction for all 12 tested phenotypes ($0.05/12 = 4.17 \times 10^{-3}$) are shaded in green. Columns and abbreviations are as previously described.

Table S18 | Clinical phenotypes of probands with *CHD7* LOF mutations probands with/without a CHARGE syndrome diagnosis

Blind ID	Gene	Age	CHD	Functional	Inheritance	Δ AA	CHARGE Dx	Deceased	Cardiac Surgery	Cath Inter	NDD	Feeding Difficulty	G-tube	Coloboma	Choanal Atresia	TEF	Airway Difficulty	Trach	Hearing loss	# ECs
1-07092	CHD7	7.0	CTD	StopGain	DNM	W145X	Y	N	N	N	NA	Y	N	Y	N	Y	Y	N	Y	5
1-15698	CHD7	4.0	AVC	StopGain	Unphased	Y192X	Y	N	Y	N	Y	Y	Y	Y	Y	N	N	N	Y	6
1-00052	CHD7	12.0	AVC	StopGain	DNM	Q217X	Y	N	Y	N	Y	Y	Y	Y	N	N	Y	N	Y	6
1-07890	CHD7	8.0	CTD	StopGain	DNM	Q518X	Y	N	Y	NA	Y	Y	N	Y	N	N	N	N	Y	4
1-02125	CHD7	9.0	CTD	FS Ins	Unphased	H533Lfs	Y	N	Y	N	NA	Y	Y	Y	NA	N	N	NA	NA	3
1-09125	CHD7	5.0	CTD	FS Del	DNM	Y835Sfs	Y	N	Y	NA	Y	Y	Y	Y	Y	N	Y	Y	Y	8
1-03550	CHD7	8.0	HLHS	Splice	Unphased	3378+1G>A	Y	N	Y	Y	Y	Y	Y	N	N	N	Y	N	Y	5
1-08360	CHD7	6.0	TOF	StopGain	DNM	R1465X	Y	N	Y	N	Y	Y	Y	Y	Y	Y	Y	N	Y	8
1-14840	CHD7	4.0	CTD	Splice	Trans	4644GGTGA>G	Y	N	Y	N	Y	Y	Y	N	N	N	N	Y	N	4
1-03954	CHD7	12.0	OTH	Splice	DNM	4645-2A>G	Y	N	Y	N	Y	Y	Y	Y	N	N	N	N	Y	5
1-16291	CHD7	2.0	TOF	FS Del	Unphased	L1551Rfs	Y	N	Y	N	Y	Y	Y	Y	Y	Y	Y	N	Y	8
1-15683	CHD7	4.0	CTD	StopGain	Unphased	Q1561X	Y	N	Y	Y	Y	Y	Y	Y	N	N	N	N	Y	5
1-00534	CHD7	27.0	TOF	StopGain	DNM	Q1599X	Y	N	Y	N	Y	Y	NA	NA	N	N	N	N	Y	3
1-06883	CHD7	7.0	TOF	StopGain	DNM	R1677X	Y	N	Y	N	Y	Y	Y	Y	N	Y	Y	N	Y	7
1-01111	CHD7	10.0	CTD	Splice	DNM	5211-1G>C	Y	Y	Y	N	Y	Y	Y	N	Y	N	Y	Y	Y	7
1-13038	CHD7	6.0	TOF	FS Ins	DNM	Y1746Cfs	Y	N	Y	Y	Y	Y	Y	Y	Y	N	Y	N	Y	7
1-13560	CHD7	23.0	TOF	StopGain	Unphased	W1772X	Y	N	Y	Y	Y	Y	Y	N	N	N	N	N	Y	4
1-10974	CHD7	19.0	CTD	StopGain	DNM	R1820X	Y	N	Y	Y	Y	Y	Y	Y	N	N	N	N	Y	5
1-07451	CHD7	8.0	CTD	FS Del	DNM	T1937fs	Y	N	Y	Y	Y	Y	Y	Y	N	N	Y	N	Y	6
1-02217	CHD7	14.0	CTD	FS Del	Unphased	G2292Efs	Y	N	Y	N	Y	Y	Y	Y	N	N	N	N	Y	5
1-06762	CHD7	7.0	HLHS	StopGain	DNM	Q2359X	Y	N	Y	Y	Y	Y	Y	N	N	N	N	N	Y	4
1-07370	CHD7	23.0	CTD	StopGain	DNM	Q2431X	Y	N	Y	Y	Y	Y	N	N	N	N	N	N	Y	3
1-02038	CHD7	9.0	CTD	StopGain	DNM	Y2601X	Y	N	N	Y	N	N	N	Y	N	N	N	N	Y	2
1-01618	CHD7	9.0	LAT	FS Del	Unphased	S307Qfs	N	N	Y	N	N	N	N	N	N	N	N	N	N	0
1-05234	CHD7	8.0	CTD	StopGain	Unphased	Q365X	N	N	Y	Y	Y	Y	Y	Y	Y	N	Y	N	Y	7
1-03501	CHD7	55.0	TOF	StopGain	Unphased	R947X	N	N	Y	Y	N	N	N	N	N	N	N	N	N	0
1-04775	CHD7	8.0	LVO	Splice	DNM	5404+1G>A	N	N	N	N	Y	N	Y	N	N	Y	Y	Y	N	5
1-04701	CHD7	8.0	TOF	Splice	Trans	5404+2T>C	N	N	Y	Y	Y	Y	Y	N	Y	Y	Y	Y	Y	8
1-07407	CHD7	0.1	AVC	StopGain	DNM	W1966X	N	Y	Y	N	NA	N	N	N	N	N	Y	N	N	1
1-00607	CHD7	10.0	LVO	StopGain	Unphased	R2284X	N	N	N	Y	Y	Y	Y	NA	N	N	N	N	Y	4
1-06220	CHD7	0.5	TOF	FS Del	DNM	T2781Qfs	N	Y	Y	N	Y	Y	N	N	N	N	Y	N	Y	4
1-01068	CHD7	11.0	CTD	FS Ins	Unphased	D2985fs	N	N	N	Y	Y	Y	N	N	N	N	Y	N	N	3
1-08200	CHD7	NA	AVC	StopGain	Unphased	R1465X	NA	NA	NA	NA	NA	NA	NA	NA	NA	NA	NA	NA	NA	NA
S6X5UMRS	CHD7	NA	TOF	StopGain	Unphased	R2418X	NA	NA	NA	NA	NA	NA	NA	NA	NA	NA	NA	NA	NA	NA
1-11950	CHD7	NA	LAT	StopGain	Unphased	R2627X	NA	NA	NA	NA	NA	NA	NA	NA	NA	NA	NA	NA	NA	NA

Thirty-five CHD probands with a *de novo* or unphased *CHD7* LOF mutation are stratified by clinical diagnosis of CHARGE syndrome (Y = Yes, N = No, NA = data not available). Probands reported to exhibit the indicated phenotypes are denoted by red "Y". NA indicates probands with insufficient data to accurately diagnose. "Total EC" denotes # of EC features, including NDD.

Table S19 | Meta-Analysis of *KMT2D* DNMs and ultra-rare TUVs in 11,555 CHD probands reveals genotype-phenotype specificity

A

Cohort				Poisson Test of LOF DNMs						Case-Control Test of LOF TUVs						Meta-Analysis
				CHD Cases		Expected		Poisson Test		CHD Cases		Controls		Fisher's Exact		
Pheno	Total	Trios	Singletons	#	Freq	#	Freq	Enrich	P-Val	#	Freq	#	Freq	Enrich	P-Val	P-Val
All	11,413	3,875	7,538	21	5.42E-03	0.17	4.27E-05	126.8	6.65E-37	12	5.5E-04	35	1.5E-04	3.6	5.49E-04	3.35E-38
CTD	2,442	631	1,811	5	7.92E-03	0.03	4.26E-05	186.0	1.14E-10	2	4.3E-04	35	1.5E-04	2.8	1.66E-01	4.86E-10
TOF	1,798	754	1,044	0	0.00E+00	0.03	4.26E-05	0.0	1.00E+00	0	0.0E+00	35	1.5E-04	0.0	1.00E+00	1.00E+00
LAT	1,968	646	1,322	0	0.00E+00	0.03	4.26E-05	0.0	1.00E+00	2	5.4E-04	35	1.5E-04	3.5	1.18E-01	3.71E-01
LVO	1,493	504	989	3	5.95E-03	0.02	4.26E-05	139.7	1.62E-06	3	1.1E-03	35	1.5E-04	7.0	1.11E-02	3.39E-07
HLHS	1,076	560	516	12	2.14E-02	0.02	4.26E-05	503.0	6.94E-29	4	2.0E-03	35	1.5E-04	12.9	3.87E-04	1.98E-30
ASD	710	216	494	0	0.00E+00	0.01	4.26E-05	0.0	1.00E+00	0	0.0E+00	35	1.5E-04	0.0	1.00E+00	1.00E+00
AVC	463	160	303	0	0.00E+00	0.01	4.26E-05	0.0	1.00E+00	0	0.0E+00	35	1.5E-04	0.0	1.00E+00	1.00E+00
OTH	1,463	404	1,059	1	2.48E-03	0.02	4.26E-05	58.1	1.71E-02	0	0.0E+00	35	1.5E-04	0.0	1.00E+00	8.67E-02
Isolated	4,617	1,238	3,379	0	0.00E+00	0.05	4.26E-05	0.0	1.00E+00	1	1.1E-04	35	1.5E-04	0.7	7.40E-01	9.63E-01
EC	1,932	1,014	918	12	1.18E-02	0.04	4.26E-05	277.8	8.47E-26	2	5.5E-04	35	1.5E-04	3.6	1.15E-01	5.92E-25
NDD	556	232	324	2	8.62E-03	0.01	4.26E-05	202.4	4.85E-05	0	0.0E+00	35	1.5E-04	0.0	1.00E+00	5.30E-04
NDD&EC	473	262	211	3	1.15E-02	0.01	4.26E-05	268.8	2.30E-07	4	4.5E-03	35	1.5E-04	29.4	1.68E-05	1.06E-10

B

Cohort				Poisson Test of D-Mis DNMs						Case-Control Test of D-Mis TUVs						Meta-Analysis
				CHD Cases		Expected		Poisson Test		CHD Cases		Controls		Fisher's Exact		
Pheno	Total	Trios	Singletons	#	Freq	#	Freq	Enrich	P-Val	#	Freq	#	Freq	Enrich	P-Val	P-Val
All	11,413	3,875	7,538	6	1.55E-03	0.44	1.14E-04	13.6	7.06E-06	96	4.4E-03	796	3.1E-03	1.4	1.56E-03	2.13E-07
CTD	2,442	631	1,811	1	1.58E-03	0.07	1.14E-04	14.0	6.92E-02	30	6.5E-03	796	3.1E-03	2.1	2.71E-04	2.23E-04
TOF	1,798	754	1,044	0	0.00E+00	0.09	1.14E-04	0.0	1.00E+00	11	3.2E-03	796	3.1E-03	1.0	5.00E-01	8.46E-01
LAT	1,968	646	1,322	0	0.00E+00	0.07	1.14E-04	0.0	1.00E+00	19	5.1E-03	796	3.1E-03	1.6	3.06E-02	1.37E-01
LVO	1,493	504	989	1	1.98E-03	0.06	1.14E-04	17.5	5.56E-02	13	4.6E-03	796	3.1E-03	1.5	1.15E-01	3.88E-02
HLHS	1,076	560	516	4	7.14E-03	0.06	1.14E-04	62.9	6.49E-07	5	2.5E-03	796	3.1E-03	0.8	7.63E-01	7.68E-06
ASD	710	216	494	0	0.00E+00	0.02	1.14E-04	0.0	1.00E+00	5	3.7E-03	796	3.1E-03	1.2	4.13E-01	7.78E-01
AVC	463	160	303	0	0.00E+00	0.02	1.14E-04	0.0	1.00E+00	2	2.3E-03	796	3.1E-03	0.7	7.60E-01	9.68E-01
OTH	1,463	404	1,059	0	0.00E+00	0.05	1.14E-04	0.0	1.00E+00	11	4.0E-03	796	3.1E-03	1.3	2.58E-01	6.07E-01
Isolated	4,617	1,238	3,379	0	0.00E+00	0.14	1.14E-04	0.0	1.00E+00	37	4.3E-03	796	3.1E-03	1.4	4.87E-02	1.96E-01
EC	1,932	1,014	918	3	2.96E-03	0.12	1.14E-04	26.0	2.34E-04	18	4.9E-03	796	3.1E-03	1.6	4.57E-02	1.33E-04
NDD	556	232	324	0	0.00E+00	0.03	1.14E-04	0.0	1.00E+00	4	3.8E-03	796	3.1E-03	1.2	4.19E-01	7.84E-01
NDD&EC	473	262	211	2	7.63E-03	0.03	1.14E-04	67.2	4.34E-04	2	2.2E-03	796	3.1E-03	0.7	7.70E-01	3.01E-03

$P_{Bonferroni} < 4.17 \times 10^{-3}$

Meta-Analysis results for *KMT2D* using 3,887 CHD proband trios and 11,555 CHD probands stratified by cardiac and extracardiac phenotypes for [**A**] LOFs and [**B**] D-mis variants. Results include Poisson test of DNMs, case-control test of TUVs, and meta-analysis of both. The meta-analysis FDR is calculated using the BH-FDR method. P-values that surpass Bonferroni correction for all 12 tested phenotypes ($0.05/12 = 4.17 \times 10^{-3}$) are shaded in green. Columns and abbreviations are as previously described.

Table S20 | Clinical phenotypes on probands with an ultra-rare or *de novo* LOF mutation in *KMT2D* or *KDM6A*

Blind ID	Gene	Age	CHD	Functional	Inheritance	Δ AA	Kabuki Dx	Deceased	Cardiac Surgery	Cath Inter	NDD	Dysmorphic Facies	Infantile Hypotonia	Feeding Difficulties	G-Tube	Limb Abnormalities
1-12480	KMT2D	10.0	HLHS	StopGain	DNM	S31X	Y	N	Y	Y	Y	Y	N	N	N	Y
1-00336	KMT2D	10.0	HLHS	Splice	DNM	839+1G>-	Y	N	Y	Y	Y	Y	Y	Y	Y	Y
1-00311	KMT2D	10.0	CTD	FS Ins	Unphased	P875Qfs	Y	N	Y	N	Y	Y	Y	Y	Y	Y
1-05786	KMT2D	14.0	CTD	FS Del	Unphased	P969Lfs	Y	N	Y	N	Y	Y	N	Y	Y	Y
1-14957	KMT2D	4.0	CTD	FS Del	DNM	S1245Yfs	Y	N	Y	Y	Y	Y	Y	Y	Y	Y
1-00596	KMT2D	9.0	HLHS	FS Del	DNM	S1722fs	Y	N	Y	Y	Y	Y	Y	Y	N	Y
1-00054	KMT2D	16.0	HLHS	FS Ins	Unphased	K2089fs	Y	N	Y	Y	Y	Y	Y	Y	Y	N
1-16121	KMT2D	5.0	CTD	StopGain	DNM	Q2540X	Y	N	Y	N	N	Y	N	Y	Y	Y
1-08230	KMT2D	6.0	LVO	StopGain	DNM	Q3531X	Y	N	Y	N	Y	Y	Y	Y	Y	Y
1-07473	KMT2D	13.0	OTH	Splice	DNM	10740+2T>A	Y	N	N	N	Y	Y	N	Y	Y	Y
1-05572	KMT2D	7.0	HLHS	StopGain	DNM	R4198X	Y	N	Y	N	Y	Y	Y	Y	N	Y
1-15932	KMT2D	11.0	LVO	StopGain	DNM	R4198X	Y	N	N	NA	Y	Y	N	Y	N	N
1-16482	KMT2D	3.0	CTD	StopGain	DNM	R4484X	Y	N	Y	Y	Y	Y	N	Y	Y	Y
1-10799	KMT2D	10.0	HLHS	StopGain	DNM	R4536X	Y	N	Y	Y	Y	Y	Y	Y	Y	N
1-08173	KMT2D	6.0	CTD	StopGain	DNM	L4551	Y	N	Y	N	Y	Y	Y	Y	N	Y
1-02566	KMT2D	8.0	LVO	FS Del	DNM	K5244fs	Y	N	Y	Y	Y	Y	Y	Y	N	Y
1-00972	KMT2D	15.0	HLHS	StopGain	Unphased	L5318X	Y	N	Y	N	Y	NA	N	N	N	Y
1-04372	KMT2D	8.0	CTD	Splice	DNM	16521+1G>T	Y	N	Y	N	Y	Y	Y	N	N	Y
1-02838	KMT2D	9.0	LVO	StopGain	Unphased	Q1893X	N	N	Y	N	N	N	N	N	N	N
1-15098	KMT2D	6.0	LVO	StopGain	Unphased	R2685X	N	N	Y	Y	Y	NA	Y	Y	Y	Y
1-04883	KMT2D	30.0	LAT	FS Del	Unphased	V3089Wfs	N	N	Y	Y	N	N	N	N	N	N
1-14537	KMT2D	8.0	HLHS	FS Del	Unphased	V3089Wfs	N	N	NA	Y	N	NA	N	N	N	NA
1-00479	KMT2D	20.0	HLHS	FS Del	DNM	F4576Cfs	N	N	Y	Y	Y	Y	N	N	N	N
1-15501	KMT2D	5.0	LVO	StopGain	DNM	R5340X	N	N	N	Y	Y	NA	Y	N	N	Y
1-13106	KDM6A	8.0	HLHS	StopGain	DNM	S936X	N	N	Y	Y	Y	N	N	Y	Y	Y
GT04010993	KMT2D	NA	HLHS	FS Ins	DNM	A254Gfs	NA	NA	NA	NA	NA	NA	NA	NA	NA	NA
GT04008173	KMT2D	NA	HLHS	StopGain	DNM	C349X	NA	NA	NA	NA	NA	NA	NA	NA	NA	NA
9504-00180-0	KMT2D	NA	HLHS	StopGain	DNM	C349X	NA	NA	NA	NA	NA	NA	NA	NA	NA	NA
92120189	KMT2D	NA	UNK	FS Del	Unphased	P799fs	NA	NA	NA	NA	NA	NA	NA	NA	NA	NA
1-07570	KMT2D	NA	HLHS	StopGain	DNM	R1757X	NA	NA	NA	NA	NA	NA	NA	NA	NA	NA
1-01099	KMT2D	NA	LAT	Splice	Unphased	5782+1G>A	NA	NA	NA	NA	NA	NA	NA	NA	NA	NA
FGT07004342	KMT2D	NA	HLHS	StopGain	Unphased	R2099X	NA	NA	NA	NA	NA	NA	NA	NA	NA	NA
9504000274-0	KMT2D	NA	HLHS	FS Del	DNM	N5079fs	NA	NA	NA	NA	NA	NA	NA	NA	NA	NA
GT04016063	KMT2D	NA	HLHS	FS Del	DNM	N5079fs	NA	NA	NA	NA	NA	NA	NA	NA	NA	NA

Thirty-four probands with an LOF mutation in *KMT2D* or *KDM6A* are stratified by the presence ("Y") absence (N) or unknown (NA) clinical diagnosis of Kabuki syndrome. Probands that were reported as exhibiting the indicated phenotypes are shown in red. NA indicates probands with insufficient data to make an accurate diagnose.

Table S21 | CHD probands with *de novo* D-mis mutations in *KMT2D*

Blind ID	Phenotype			Mutation			Annotation		
	Cardiac	Extracardiac	Kabuki	Locus	Ref	Alt	ΔAA	Bravo MAF	ClinVar
GT04012081	HLHS	EC	NA	12:49420288	C	T	R5154Q	0.0E+00	Uncertain
1-08730	CTD	EC	N	12:49420213	C	T	R5179H	0.0E+00	Pathogenic
1-01925	HLHS	NDD+EC	Y	12:49418630	C	G	G5295A	0.0E+00	.
1-03948	HLHS	EC	N	12:49418361	C	T	R5351Q	0.0E+00	.
1-15748	LVO	NDD+EC	N	12:49418361	C	T	R5351Q	0.0E+00	.
1-00780	HLHS	Unknown	N	12:49416444	C	A	V5423F	0.0E+00	.

Mutation listed as "pathogenic" is pathogenic for Kabuki syndrome; dots indicate the variant has not been reported previously. Abbreviations as defined previously.

Table S22 | Case-control analysis of ClinVar pathogenic and other missense mutations in RASopathy-associated genes

Mutation Set	RASopathy-associated Genes in Panel (n=7)						*PTPN11*					
	CHD Cases		gnomAD Controls		Fisher's Exact		CHD Cases		gnomAD Controls		Fisher's Exact	
	#	Freq	#	Freq	Enrich	P-Val	#	Freq	#	Freq	Enrich	P-Val
All Missense	304	1.3E-02	2,631	9.7E-03	1.4	**1.50E-07**	59	2.6E-03	226	8.3E-04	3.2	**2.01E-12**
Damaging Missense	115	5.1E-03	623	2.3E-03	2.2	**6.77E-13**	48	2.1E-03	110	4.1E-04	5.3	**4.06E-17**
ClinVar Pathogenic Missense*	90	4.0E-03	40	1.5E-04	26.9	**1.40E-68**	57	2.5E-03	23	8.5E-05	30.0	**2.09E-45**
ClinVar Benign/NS Missense*	251	1.1E-02	9,956	3.7E-02	0.3	1.00E+00	20	8.9E-04	307	1.1E-03	0.8	8.74E-01

Case-control analyses of different categories of missense mutations in seven RASopathy genes are shown on the left, and for variants in just PTPN11 on the right. ClinVar pathogenic missense variants in both groups are much more highly enriched that all damaging missense mutations, consistent with recurrent ClinVar variants for these genes being gain of function rather than LOF. Simlarly, very ClinVar Benign/NS (Not Submitted) variants show no enrichment, Because these gain of function mutations occur at only selected sites, functional mutations are recurrent, and consequently in disease populations they have generally higher allele frequencies, but still are rare in controls. For these analyses, we had no allele frequency filters for ClinVar Pathogenic missense mutations. P- values were calculated using a one-sided Fisher's exact test with $P < 0.05$ colored in red. The asterisk denotes that the in-cohort variant filter was removed for these tests (see Methods).

Table S23 | Clinical phenotypes on probands with a ClinVar pathogenic missense mutation or a clinical RASopathy or Noonan syndrome diagnosis

A

Blind ID	Age	CHD	Gene	Inher	Δ AA	Noonan Dx	Deceased	Cardiac Surgery	STAT Score	NDD	Dysmorphic	Height (%)	Arrhythmia
1-05187	10	CTD	BRAF	Unphas	Q257R	Y	N	N	0	Y	Y	7	Y
1-01011	12	AVC	BRAF	DNM	T241M	Y	N	Y	2	Y	Y	10	N
1-05765	34	LVO	PTPN11	Unphas	T42A	Y	N	Y	2	N	NA	<3	N
1-15354	9	CTD	PTPN11	Unphas	N58H	Y	N	Y	1	N	N	<3	N
1-06605	14	CTD	PTPN11	Unphas	D61N	Y	N	Y	2	Y	NA	<3	N
1-08260	7	CTD	PTPN11	Unphas	D61N	Y	N	Y	2	N	Y	<3	Y
1-03322	12	ASD	PTPN11	Unphas	Y63C	Y	N	Y	1	NA	NA	15	NA
1-09316	6	CTD	PTPN11	Unphas	Y63C	Y	N	Y	2	Y	Y	<3	Y
1-05388	8	CTD	PTPN11	Unphas	Y63C	Y	N	Y	2	NA	Y	<3	N
1-11291	6	CTD	PTPN11	Unphas	E76D	Y	N	y	2	Y	Y	15	N
1-08094	7	CTD	PTPN11	DNM	E139D	Y	N	Y	1	Y	Y	25	N
1-11385	18	TOF	PTPN11	Unphas	E139D	Y	N	Y	2	N	N	<3	N
1-18042	24	LAT	PTPN11	Unphas	Q256R	Y	N	Y	4	N	Y	<3	Y
1-03566	8	CTD	PTPN11	Unphas	R265Q	Y	N	Y	2	NA	NA	<3	N
1-26017	2	CTD	PTPN11	Unphas	I282M	Y	N	N	0	Y	NA	10	N
1-03749	21	CTD	PTPN11	Unphas	F285I	Y	N	Y	2	N	NA	10	N
1-10028	10	LVO	PTPN11	Unphas	F285S	Y	N	Y	2	Y	NA	7	N
1-06221	21	CTD	PTPN11	Unphas	N308D	Y	N	N	0	Y	Y	<3	NA
1-04664	23	AVC	PTPN11	Trans	N308D	Y	N	Y	2	Y	NA	<3	N
1-09816	13	CTD	PTPN11	Unphas	N308D	Y	N	Y	1	N	Y	5	N
1-03389	13	CTD	PTPN11	Unphas	N308D	Y	N	Y	2	Y	Y	<3	NA
1-05209	29	ASD	PTPN11	DNM	N308D	Y	N	Y	4	N	NA	<3	Y
1-05022	29	CTD	PTPN11	DNM	N308D	Y	N	Y	1	N	NA	20	N
1-04086	38	CTD	PTPN11	Unphas	N308S	Y	N	Y	1	N	NA	<3	N
1-10465	0	CTD	PTPN11	DNM	N308T	Y	N	Y	2	N	Y	<3	Y
1-02308	20	LVO	PTPN11	Unphas	N308T	Y	N	Y	2	Y	Y	<3	N
1-00909	10	AVC	PTPN11	DNM	N308S	Y	N	Y	3	Y	Y	NA	N
1-03495	17	CTD	PTPN11	Unphas	N308S	Y	N	N	0	N	NA	50	NA
1-22600	3	ASD	PTPN11	Unphas	G503R	Y	N	Y	1	Y	Y	<3	N
1-16456	3	AVC	PTPN11	Unphas	M504V	Y	N	Y	5	Y	Y	<3	N
1-01452	14	CTD	PTPN11	Unphas	M504V	Y	N	Y	1	Y	Y	<3	N
1-00040	10	OTH	RAF1	DNM	V263G	Y	N	Y	2	Y	Y	50	Y
1-02558	15	LVO	RAF1	DNM	P261L	Y	N	Y	2	N	Y	<3	N
1-01061	29	CTD	RAF1	DNM	S257L	Y	N	Y	2	Y	Y	15	Y
1-04226	21	ASD	RAF1	DNM	S257L	Y	N	Y	2	Y	NA	<3	Y
1-09524	6	CTD	RIT1	Unphas	M90I	Y	N	Y	2	Y	Y	5	N
1-00843	19	CTD	RIT1	DNM	A57G	Y	N	Y	2	Y	Y	5	Y
1-05555	9	CTD	RIT1	DNM	A57G	Y	N	N	0	Y	NA	10	Y
1-15572	5	CTD	RIT1	Unphas	S35T	Y	N	N	0	N	N	80	N
1-05057	20	CTD	RIT1	Unphas	K23Q	Y	N	Y	2	Y	NA	25	N
1-05303	35	CTD	SHOC2	Unphas	S2G	Y	N	N	0	Y	Y	<3	Y
1-00321	11	CTD	SOS1	DNM	R552G	Y	N	Y	2	Y	Y	5	N
1-15793	38	CTD	SOS1	Unphas	R552G	Y	N	Y	2	N	N	10	N
1-01405	21	AVC	SOS1	DNM	W432R	Y	N	Y	4	N	Y	7	Y
1-17180	3	CTD	SOS1	Unphas	T378A	Y	N	Y	2	Y	Y	<3	NA
1-07441	9	CTD	SOS1	Unphas	M269T	Y	N	Y	2	Y	Y	25	N
1-02458	19	OTH	SOS1	DNM	T266K	Y	N	Y	1	Y	NA	<3	N

B

Blind ID	Age	CHD	Gene	Functional	Inher	Δ AA	Noonan Dx	Deceased	Cardiac Surgery	STAT Score	NDD	Dysmorphic	Height (%)	Arrhythmia
1-01961	14	ASD	BRAF	D-Mis	Unphas	T589A	N	N	N	0	Y	N	<3	N
1-02437	9	LAT	LZTR1	D-Mis	DNM	G248R	N	N	Y	2	N	N	NA	N
1-03497	10	CTD	LZTR1	D-Mis	Unphas	G248R	N	N	Y	2	N	NA	50	N
1-00873	35	AVC	LZTR1	D-Mis	Unphas	G248R	N	N	Y	4	N	NA	45	N
1-15228	12	CTD	LZTR1	D-Mis	Unphas	G248R	N	N	Y	2	NA	NA	7	N
1-03542	9	CTD	LZTR1	D-Mis	Unphas	R284C	N	N	Y	2	Y	NA	30	N
1-12470	6	CTD	PTPN11	D-Mis	Unphas	N58S	N	N	Y	1	N	NA	50	N
1-02601	19	CTD	PTPN11	D-Mis	Unphas	G60S	N	N	N	0	Y	N	<3	N
1-06042	64	CTD	PTPN11	D-Mis	Unphas	Y63C	N	Y	Y	2	N	NA	7	Y
1-12206	7	ASD	PTPN11	D-Mis	Unphas	E69Q	N	N	Y	1	Y	Y	10	N
1-05083	12	CTD	PTPN11	D-Mis	Unphas	A72S	N	N	N	0	N	NA	<3	N
1-17490	13	CTD	PTPN11	D-Mis	Unphas	E110K	N	N	N	0	NA	NA	<3	N
1-01429	4	LAT	PTPN11	D-Mis	Unphas	R152H	N	Y	Y	5	Y	NA	5	N
1-03683	10	CTD	PTPN11	T-Mis	DNM	N200Y	N	N	N	0	Y	NA	15	N
1-03874	12	OTH	PTPN11	D-Mis	DNM	G268C	N	N	Y	2	N	NA	7	N
1-02075	17	CTD	PTPN11	T-Mis	Unphas	I282V	N	N	Y	1	N	N	<3	N
1-16190	19	LVO	PTPN11	D-Mis	Unphas	N308D	N	N	Y	3	NA	NA	10	N
1-06749	11	CTD	PTPN11	D-Mis	Trans	N308D	N	N	Y	1	N	N	25	N
1-06223	25	LVO	PTPN11	D-Mis	Unphas	N308D	N	N	N	0	N	NA	<3	N
1-06938	8	LVO	PTPN11	D-Mis	Unphas	N308D	N	N	Y	2	NA	NA	10	N
1-13755	9	CTD	PTPN11	D-Mis	Unphas	N308D	N	N	Y	2	N	N	<3	N
1-06437	15	CTD	PTPN11	D-Mis	Unphas	N308D	N	N	Y	1	Y	N	50	N
1-02149	10	CTD	PTPN11	D-Mis	Unphas	N308S	N	N	N	0	N	Y	<3	N
1-04111	9	LVO	PTPN11	D-Mis	Unphas	V428M	N	N	Y	2	N	N	5	N
1-15770	5	OTH	PTPN11	D-Mis	Unphas	R498W	N	N	Y	2	N	N	90	N
1-15939	5	TOF	PTPN11	D-Mis	Trans	Q510R	N	N	Y	2	N	N	50	N
1-04171	Unk	OTH	RAF1	D-Mis	Unphas	S257L	N	N	Y	4	N	Y	NA	N
1-04886	25	CTD	RIT1	D-Mis	Unphas	A57G	N	N	Y	2	N	NA	<3	NA
1-06833	25	LVO	SOS1	T-Mis	Unphas	Y702C	N	N	N	0	N	N	75	Y
1-14673	54	CTD	RIT1	D-Mis	Unphas	G95A	NA	N	Y	2	N	N	5	Y
1-06224	31	CTD	SOS1	D-Mis	Unphas	F623V	NA	N	Y	2	N	N	25	NA
S64E9O2I	NA	OTH	BRAF	D-Mis	Unphas	N581D	NA	NA	NA	NA	NA	NA	NA	NA
1-06253	NA	AVC	PTPN11	D-Mis	DNM	Y62D	NA	NA	NA	NA	NA	NA	NA	NA
1-09538	NA	CTD	PTPN11	D-Mis	Unphas	Y62D	NA	NA	NA	NA	NA	NA	NA	NA
1-07624	NA	CTD	PTPN11	D-Mis	Unphas	E258D	NA	NA	NA	NA	NA	NA	NA	NA
1-00416	NA	OTH	PTPN11	D-Mis	DNM	N308D	NA	NA	NA	NA	NA	NA	NA	NA
1-13623	NA	CTD	PTPN11	D-Mis	Unphas	N308D	NA	NA	NA	NA	NA	NA	NA	NA
1-03011	NA	LVO	PTPN11	D-Mis	Unphas	N308D	NA	NA	NA	NA	NA	NA	NA	NA
1-00966	NA	CTD	PTPN11	D-Mis	DNM	S502L	NA	NA	NA	NA	NA	NA	NA	NA
1-03347	NA	ASD	PTPN11	D-Mis	DNM	G503E	NA	NA	NA	NA	NA	NA	NA	NA
FGT07004653	NA	HLHS	RAF1	D-Mis	Unphas	P261A	NA	NA	NA	NA	NA	NA	NA	NA
1-04489	NA	CTD	RIT1	D-Mis	Unphas	A57G	NA	NA	NA	NA	NA	NA	NA	NA
1-04199	NA	CTD	RIT1	D-Mis	Trans	A57G	NA	NA	NA	NA	NA	NA	NA	NA

Tables shows 90 probands with a ClinVar pathogenic missense mutation in a RASopathy-associated gene (*BRAF*, *LZTR1*, *PTPN11*, *RAF1*, *RIT1*, *SHOC2*, *SOS1*). **[A]** Forty-seven probands with a clinical RASopathy diagnosis are show, along with clinical features and surgical STAT score. Heigh is the percentile for age at last measurement. **[B]** Twenty-nine probands with a negative clinical diagnosis and fourteen probands without data regarding a RASopathy diagnosis are shown.

Table S24 | Phenotypic characteristics of Rasopathy-associated CHD probands

A

Gene	Cardiac Phenotype	Clinically Diagnosed RASopathy Probands	% CHD Probands with ClinVar+ Mutation		
			All	RASopathy DX	No RASopathy Dx
RASopathy-associated	*Total Patient #*	*(Reported)*	*(n = 78)*	*(n = 47)*	*(n = 29)*
	PS	50-60%	69.2%	70.2%	65.5%
	ASD	6-10%	41.0%	42.6%	34.5%
	HCM or Sub-AS	20%	15.4%	21.3%	6.9%
	Poly-Valvlar	< 5%	14.1%	17.0%	10.3%
	BAV or CoA	< 5%	7.7%	6.4%	10.3%
	AVC	< 5%	9.0%	12.8%	3.4%
	Laterality	NR	2.6%	0.0%	6.9%
	TOF	< 5%	2.6%	2.1%	3.4%
PTPN11	*Total Patient #*	*(Reported)*	*(n = 49)*	*(n = 29)*	*(n = 20)*
	PS	50-60%	67.3%	65.5%	70.0%
	ASD	6-10%	42.9%	44.8%	40.0%
	HCM or Sub-AS	20%	10.2%	13.8%	5.0%
	Poly-Valvar	< 5%	10.2%	10.3%	10.0%
	BAV or CoA	< 5%	10.2%	10.3%	10.0%
	AVC	< 5%	8.2%	13.8%	0.0%
	Laterality	NR	2.0%	0.0%	5.0%
	TOF	< 5%	4.1%	0.0%	10.0%

B

Patient Characteristic	% CHD Probands with ClinVar+ Mutation in RASopathy Gene			
	All	Arrhythmia Dx	No Arrhythmia Dx	P-Value
Total Patient #	*(n = 78)*	*(n = 16)*	*(n = 55)*	-
RASopathy Dx	60.0%	86.6%	52.7%	**1.9E-02**
HCM or Sub-AS	16.9%	31.2%	12.7%	0.13
PTPN11 ClinVar+	37.5%	37.5%	70.9%	**2.0E-02**
STAT Score $\bar{x}$ ± SD	1.7 ± 1.2	1.88 ± 1.36	1.66 ± 1.14	0.43
Age $\bar{x}$ ± SD	15.8 ± 11.5	22.7 ± 17.3	13.5 ± 8.7	**4.6E-02**

[A] Table compares prevalence of cardiac phenotypes in 78 probands with a ClinVar pathogenic missense mutation in a RASopathy-associated gene to those reported in the literature and between probands who were and were not clinically diagnosed with Noonan syndrome. **[B]** Patient characteristics among 78 probands that had a ClinVar pathogenic missense mutation in a RASopathy-associated gene and among those evaluated for presence of a cardiac arrhythmia, excluding those that could not be conclusively diagnosed. The average clinical STAT score and age for each patient is shown plus/minus the standard deviation and, for age, with the minimum-maximum ages. P-values for binary data and continuous variables were calculated using a two-sided Fisher's Exact Test and Wilcoxon rank-sum test, respectively. P-values < 0.05 are colored in red.

Table S25 | Frequency of clinical diagnosis in probands with ClinVar+ Mutations in RASopathy-related genes

Gene	RASopathy Diagnosis for Probands with a ClinVar+ RASopathy Mutation			
	Yes	No	Unknown	All
SHOC2	1 (100.0%)	0 (0.0%)	0 (0.0%)	1 (1.3%)
RAF1	4 (80.0%)	1 (20.0%)	0 (0.0%)	5 (6.4%)
SOS1	6 (75.0%)	1 (12.5%)	1 (12.5%)	8 (10.3%)
RIT1	5 (71.4%)	1 (14.3%)	1 (14.3%)	7 (9.0%)
BRAF	2 (66.7%)	1 (33.3%)	0 (0.0%)	3 (3.8%)
PTPN11	29 (59.2%)	20 (40.8%)	0 (0.0%)	49 (62.8%)
LZTR1	0 (0.0%)	5 (100.0%)	0 (0.0%)	5 (6.4%)
**Total**	**47 (60.3%)**	**29 (37.2%)**	**2 (2.6%)**	**78 (100%)**

Table shows number of probands with a ClinVar pathogenic mutation in RASopathy-related gene present in the MIPS panel who were and were not clinically diagnosed with Noonan/RASopathy. Column ”All” shows the contribution of mutations in each gene to the total number of RASopathy probands (n = 78).

<u>Dataset S1 (separate file)</u> Genes in the 248 gene panel

248 genes included in MIPseq panel with annotation for gene selection criteria. "Inclusion criteria" provides the primary reason for addition to the panel as represented in Figure S1. 'Damaging DNMs' is number of Damaging DNMs identified in CHD probands per gene in Homsy, et. al. Nat Gen 2015. The "e14.5 Mouse Heart Expression" uses the calculated percentile as reported in Homsy, et. al. Nat Gen 2015. ClinGen is the ClinGen Gene-Disease Validity Curations Classification (retrieved 3 June 2024). "Prior Sig" is if the gene has been reported in the literature to have a statistically significant association in unrelated probands at the time of panel gene selection.

<u>Dataset S2 (separate file)</u> MIPseq Probes for 248 Gene Panel

Table shows the MIPgen probe design file and associated relevant calculations for the MIPs included in the MIPseq 248 gene panel. "Target_GC" is the percent of G or C bases in the probe target sequence. "Pool" is sequencing pool assigned for the probe in the MIPseq protocol (see Fig S4)."Gene" is the primary gene targeted by the probe per NCBI RefSeq b37. Other columns are as described in MIPgen manual [Boyle, etl. al. Bioinformatics 2014].

<u>Dataset S3 (separate file)</u> All 11,555 MIPseq and WES Probands

Table of all CHD probands included in study. The 'Proband ID' is de-identified identifier. 'Platform' is the sequencing platform. 'Family structure' indicates if the proband is part of a complete trio that was included in the study or a singleton. 'Cardiac Summary Diagnosis' is the top-level cardiac phenotype assigned to the proband. 'Detailed Cardiac Diagnosis' is the complete clinical description of the cardiac phenotype. 'NDD/EC Summary Diagnosis' is the top-level NDD and EC phenotype assigned to the proband.

<u>Dataset S4 (separate file)</u> List of all variants included in analysis within 248 panel genes

Table of all mutations in probands included in study, including both DNMs and TUVs. The 'Proband ID' is de-identified identifier. 'Platform' is the sequencing platform. 'Family structure' indicates if the proband is part of a complete trio that was included in the study or a singleton. 'Cardiac Summary Diagnosis' is the top-level cardiac phenotype assigned to the proband. 'Detailed Cardiac Diagnosis' is the complete clinical description of the cardiac phenotype. 'NDD/EC Summary Diagnosis' is the top-level NDD and EC phenotype assigned to the proband. The 'Chr', 'Pos', 'Ref', and 'Alt' are using hg19 coordinates. 'GT' is genotype. 'Transmission' is, if known, the parental transmission status. 'Gene' is the assigned gene in hg19 and, if in the CDS of multiple genes, prioritizes the gene targetted in the panel. 'Functional' is the predicted functional protein change caused by the mutation (LOF, D-Mis, T-Mis, Syn) as previously defined and using the most deleterious result among all major isoforms. 'Δ AA' is the predicted amino acid or splice change caused by the mutation in the corresponding transcript. 'MetaSVM' is the predicted missense deleteriousness from the MetaSVM tool (see Methods). 'ESP', "BRAVO', and 'gnomAD"

columns indicate the allele frequency in the corresponding database (see Methods). The 'Included in Meta-Analysis' column indicates if the specific variant was included in the Meta-Analysis of DNMs and TUVs (passes all cutoffs; see Methods). The 'Significant Gene in Meta-Analysis' column indicates if that gene was among of the 60 with a significant JL-FDR.

Dataset S5 (separate file) List of all genome-wide DNMs included in analysis among 3,887 WES trios

Table of all DNMs genome-wide included in study among the 3,887 WES trios. The 'Proband ID' is de-identified identifier'Cardiac Summary Diagnosis' is the top-level cardiac phenotype assigned to the proband. 'Detailed Cardiac Diagnosis' is the complete clinical description of the cardiac phenotype. 'NDD/EC Summary Diagnosis' is the top-level NDD and EC phenotype assigned to the proband. The 'Chr', 'Pos', 'Ref', and 'Alt' are using hg19 coordinates. 'GT' is genotype. 'Gene' is the assigned gene by annovar (see Methods). 'Functional' is the predicted functional protein change caused by the mutation (LOF, D-Mis, T-Mis, Syn) as previously defined and using the most deleterious result among all major isoforms. 'LOF Detail' provides more specific classification for LOF variants.

Dataset S6 (separate file) Meta-Analysis results for MIPseq Panel Genes

Table shows all 248 MIPseq panel genes from meta-analysis for [left] damaging, [mid] LOF, and [right] D-Mis variants using the burden of de novo mutations in CHD trios and case-control comparison of the TUVs in 11,555 CHD probands compared to gnomAD controls. Red shading denotes p-values that surpass genome-wide significance and blue denotes an FDR < 0.05. All columns and abbreviations are as previously described.

Dataset S7 (separate file) Results for TDT on all MIPseq panel Genes

TDT results for individual genes that had over-transmission of D-mis and LOF variants. Abbreviations are: 'Sig 60' indicates if this gene is among the 60 genes significantly enriched in the primary Damaging variant meta-analysis; ‘Trans’ is # of transmitted variants from parents to proband; ‘Non-Trans’ is # of variants not transmitted; “% T” is the percent of variants that were transmitted; ‘enrich’ is enrichment; and p-value is from the chi-square test. Other abbreviations are as previously defined. P-values < 0.05 are colored in red and FDRs < 0.05 are colored in blue. Genes are sorted by alphabetical order.

Dataset S8 (separate file) List of D-Mis MYH6 variants in probands and parents used in TDT Analysis

Table of all D-Mis MYH6 mutations from probands and parents included in TDT analysis. The 'Proband ID' is de-identified identifier. The 'Chr', 'Pos', 'Ref', and 'Alt' are using hg19 coordinates. 'Δ AA' is the predicted amino acid or splice change caused by the mutation in the corresponding transcript. 'Transmission' shows the inheritance category for the variant: transmitted from mother or father to proband, not transmitted from parent, or de novo in the proband. 'CisHet' indicates if the variant is

transmitted from the same parent as another mutation in the same gene. 'Proband Diagnosis' is the top-level cardiac phenotype assigned to the proband (see Methods). The 'Structural Heart Disease' columns in maternal or paternal pedigrees.

References to Extended Methods

1. B. Gelb *et al.*, The Congenital Heart Disease Genetic Network Study: rationale, design, and early results. *Circ Res* **112**, 698-706 (2013).
2. S. C. Jin *et al.*, Contribution of rare inherited and de novo variants in 2,871 congenital heart disease probands. *Nat Genet* **49**, 1593-1601 (2017).
3. S. Purcell *et al.*, PLINK: a tool set for whole-genome association and population-based linkage analyses. *Am J Hum Genet* **81**, 559-575 (2007).
4. S. Zaidi *et al.*, De novo mutations in histone-modifying genes in congenital heart disease. *Nature* **498**, 220-223 (2013).
5. K. J. Karczewski *et al.*, The mutational constraint spectrum quantified from variation in 141,456 humans. *Nature* **581**, 434-443 (2020).
6. E. A. Boyle, B. J. O'Roak, B. K. Martin, A. Kumar, J. Shendure, MIPgen: optimized modeling and design of molecular inversion probes for targeted resequencing. *Bioinformatics* **30**, 2670-2672 (2014).
7. D. Karolchik *et al.*, The UCSC Table Browser data retrieval tool. *Nucleic Acids Res* **32**, D493-496 (2004).
8. S. Cantsilieris, H. A. Stessman, J. Shendure, E. E. Eichler, Targeted Capture and High-Throughput Sequencing Using Molecular Inversion Probes (MIPs). *Methods Mol Biol* **1492**, 95-106 (2017).
9. J. Zhang, K. Kobert, T. Flouri, A. Stamatakis, PEAR: a fast and accurate Illumina Paired-End reAd mergeR. *Bioinformatics* **30**, 614-620 (2014).
10. H. Li, Aligning sequence reads, clone sequences and assembly contigs with BWA-MEM. *bioRxiv doi:1303.3997 [q-bio.GN]*.
11. A. R. Quinlan, I. M. Hall, BEDTools: a flexible suite of utilities for comparing genomic features. *Bioinformatics* **26**, 841-842 (2010).
12. A. Tarasov, A. J. Vilella, E. Cuppen, I. J. Nijman, P. Prins, Sambamba: fast processing of NGS alignment formats. *Bioinformatics* **31**, 2032-2034 (2015).
13. G. A. Van der Auwera *et al.*, From FastQ data to high confidence variant calls: the Genome Analysis Toolkit best practices pipeline. *Curr Protoc Bioinformatics* **43**, 11 10 11-11 10 33 (2013).
14. G. M. Erik Garrison, Haplotype-based variant detection from short-read sequencing. *arXiv doi:1207.3907 [q-bio.GN]* (2012).

15. K. Wang, M. Li, H. Hakonarson, ANNOVAR: functional annotation of genetic variants from high-throughput sequencing data. *Nucleic Acids Res* **38**, e164 (2010).
16. H. Li, A statistical framework for SNP calling, mutation discovery, association mapping and population genetical parameter estimation from sequencing data. *Bioinformatics* **27**, 2987-2993 (2011).
17. J. M. Zook *et al.*, Extensive sequencing of seven human genomes to characterize benchmark reference materials. *Sci Data* **3**, 160025 (2016).
18. Q. Wei *et al.*, A Bayesian framework for de novo mutation calling in parents-offspring trios. *Bioinformatics* **31**, 1375-1381 (2015).
19. P. I. de Bakker *et al.*, Practical aspects of imputation-driven meta-analysis of genome-wide association studies. *Hum Mol Genet* **17**, R122-128 (2008).
20. S. Lee, G. R. Abecasis, M. Boehnke, X. Lin, Rare-variant association analysis: study designs and statistical tests. *Am J Hum Genet* **95**, 5-23 (2014).
21. C. Dering, C. Hemmelmann, E. Pugh, A. Ziegler, Statistical analysis of rare sequence variants: an overview of collapsing methods. *Genet Epidemiol* **35 Suppl 1**, S12-17 (2011).
22. S. Lee *et al.*, Optimal unified approach for rare-variant association testing with application to small-sample case-control whole-exome sequencing studies. *Am J Hum Genet* **91**, 224-237 (2012).
23. S. Flygare *et al.*, The VAAST Variant Prioritizer (VVP): ultrafast, easy to use whole genome variant prioritization tool. *BMC Bioinformatics* **19**, 57 (2018).
24. L. Moutsianas *et al.*, The power of gene-based rare variant methods to detect disease-associated variation and test hypotheses about complex disease. *PLoS Genet* **11**, e1005165 (2015).
25. Anonymous (The NHLBI Trans-Omics for Precision Medicine (TOPMed) Whole Genome Sequencing Program. BRAVO variant browser: University of Michigan and NHLBI; 2018. Available from: bravo.sph.umich.edu/freeze5/hg38/.
26. Anonymous (Exome Variant Server, NHLBI GO Exome Sequencing Project (ESP), Seattle, WA (URL: evs.gs.washington.edu/EVS) [Accessed: 29 October 2017].
27. M. Haeussler *et al.*, The UCSC Genome Browser database: 2019 update. *Nucleic Acids Res* **47**, D853-D858 (2019).
28. Z. He *et al.*, Rare-variant extensions of the transmission disequilibrium test: application to autism exome sequence data. *Am J Hum Genet* **94**, 33-46 (2014).
29. N. Risch, K. Merikangas, The future of genetic studies of complex human diseases. *Science* **273**, 1516-1517 (1996).

30. W. Jiang, W. Yu, Controlling the joint local false discovery rate is more powerful than meta-analysis methods in joint analysis of summary statistics from multiple genome-wide association studies. *Bioinformatics* **33**, 500-507 (2017).
31. J. Wakefield, A Bayesian measure of the probability of false discovery in genetic epidemiology studies. *Am J Hum Genet* **81**, 208-227 (2007).
